# Supplementary material for: Evaluation of hydroxychloroquine or chloroquine for the prevention of COVID-19 (COPCOV): A double-blind, randomised, placebo-controlled trial
Source: PLoS Med. 2024 Sep 12;21(9):e1004428. doi: 10.1371/journal.pmed.1004428 (PMC11392261; doi:10.1371/journal.pmed.1004428)
Supplement: S1 Protocol — (DOCX) [file pmed.1004428.s003.docx]

**Study Title:** Chloroquine/ hydroxychloroquine prevention of coronavirus disease (COVID-19) in the healthcare setting; a randomised, placebo-controlled prophylaxis study (COPCOV)

**Internal Reference Number / Short title:** COPCOV

**OxTREC Ref:** 25-20

**Protocol number**: VIR20001

**Date and Version No:** Version 6.0 dated 13 January 2021

| **Co-Principal Investigators:** | **Professor Sir Nicholas White** ([nickw@tropmedres.ac](mailto:nickw@tropmedres.ac))  Chairman Wellcome Trust Southeast Asian Tropical Medicine Programmes & Consultant Physician  Mahidol Oxford Tropical Medicine Research Unit, Thailand  **Dr William Schilling** ([william@tropmedres.ac](mailto:william@tropmedres.ac))  Research Physician & Infectious Diseases/ Microbiology Registrar  Mahidol Oxford Tropical Medicine Research Unit, Thailand |
| --- | --- |
| **Investigators:** | **Professor Nicholas Day**  Director & Consultant Physician  Mahidol Oxford Tropical Medicine Research Unit, Thailand  **Dr Walter Taylor**  Senior Clinical Researcher and Infectious Diseases Physician  Mahidol Oxford Tropical Medicine Research Unit, Thailand  **Dr Elizabeth Ashley**  Director & Consultant Clinical Microbiologist/ Infectious Diseases Physician  Lao-Oxford-Mahosot Hospital-Wellcome Trust Research Unit, Lao PDR  **Associate Professor Phaik Yeong Cheah**  Head of Bioethics & Engagement  Mahidol Oxford Tropical Medicine Research Unit, Thailand  **Professor Arjen Dondorp**  Deputy Director and Head of Malaria & Critical Illness  Mahidol Oxford Tropical Medicine Research Unit, Thailand  **Professor Amanda Adler**  Consultant Physician and Director of the Diabetes Trials Unit  The University of Oxford  **Associate Professor Mayfong Mayxay**  Head of Malaria and Field Research, Vice President of the Lao National University of Health Sciences  Lao-Oxford-Mahosot Hospital-Wellcome Trust Research Unit, Lao PDR  **Assistant Professor Weerapong Phumratanaprapin**  Dean of the Faculty of Tropical Medicine  Mahidol University, Thailand  **Professor Yoong Poovorawan**  Professor of Paediatrics,  Chulalongkorn University, Bangkok, Thailand.  **Dr James Callery**  Research Physician  Mahidol Oxford Tropical Medicine Research Unit, Thailand  **Dr Arjun Chandna**  Research Physician & Infectious Diseases/ Microbiology Registrar  Cambodia-Oxford Medical Research Unit, Cambodia  **Professor Paul Turner**  Director & Consultant Clinical Microbiologist  Cambodia-Oxford Medical Research Unit, Cambodia  **Dr Cintia Cruz**  Paediatrician and Clinical Pharmacologist  Mahidol Oxford Tropical Medicine Research Unit, Thailand  **Associate Professor Lorenz von Seidlein**  Senior Clinical Researcher and Infectious Diseases Physician  Mahidol Oxford Tropical Medicine Research Unit, Thailand  **Dr Dora Buonafrate**  Infectious Disease Physician  Centre for Tropical Diseases, Sacro Cuore Don Calabria Hospital, Italy  **Professor Piero Olliaro**  Infectious Diseases Physician  Nuffield Department of Medicine, Oxford University, UK  **Professor Martin Llewelyn**  Consultant in Infectious Diseases  Brighton and Sussex Medical School, UK  **Dr Sophie Yacoub**  Consultant in Infectious Diseases and General Medicine  Oxford University Clinical Research Unit/ Wellcome Programme, Vietnam  **Professor Guy Thwaites**  Director & Consultant Clinical Microbiologist/ Infectious Diseases Physician  Oxford University Clinical Research Unit/ Wellcome Programme, Vietnam  **Dr Mehul Dhorda**  Senior Scientist  Mahidol Oxford Tropical Medicine Research Unit, Thailand |
| **Sponsor:** | The University of Oxford |
| **Funder:**  **Signatures:** | ACT-Accelerator Therapeutics Partnership |

**Professor Sir Nicholas White** _______________________ ______________________

(Co-Principal Investigator) (Signature) (Date)

**Dr William Schilling** _______________________ ______________________

(Co-Principal Investigator) (Signature) (Date)

**Confidentiality Statement**

This document contains confidential information that must not be disclosed to anyone other than the authorised individuals from the University of Oxford, the Investigator Team and members of the Oxford Tropical Research Ethics Committee (OxTREC) and local Ethics Committee, unless authorised to do so.

**TABLE OF CONTENTS**

[1. SYNOPSIS 7](#_Toc61457958)

[2. ABBREVIATIONS 9](#_Toc61457959)

[3. BACKGROUND AND RATIONALE 10](#_Toc61457960)

[4. OBJECTIVES AND OUTCOME MEASURES 15](#_Toc61457961)

[5. STUDY DESIGN 16](#_Toc61457962)

[6. PARTICIPANT IDENTIFICATION AND RECRUITMENT 18](#_Toc61457963)

[6.1. Study Participants 18](#_Toc61457964)

[6.2. Inclusion Criteria 18](#_Toc61457965)

[6.3. Exclusion Criteria 19](#_Toc61457966)

[7. STUDY PROCEDURES 19](#_Toc61457967)

[7.1. Recruitment 19](#_Toc61457968)

[7.2. Screening and Eligibility Assessment 20](#_Toc61457969)

[7.3. Informed Consent 20](#_Toc61457970)

[7.4. Clinical examination 20](#_Toc61457971)

[7.5. Randomisation and blinding 20](#_Toc61457972)

[7.6. Baseline Assessments 21](#_Toc61457973)

[7.7. Subsequent Visits 21](#_Toc61457974)

[7.8. Sample Handling 22](#_Toc61457975)

[7.9. Discontinuation/Withdrawal of Participants from Study 23](#_Toc61457976)

[7.10. Definition of End of Study 24](#_Toc61457977)

[8. STUDY MEDICATION (CHLOROQUINE or HYDROXYCHLOROQUINE/PLACEBO) 24](#_Toc61457978)

[8.1. Study Medication Description 24](#_Toc61457979)

[8.2. Storage of Study Medication 24](#_Toc61457980)

[8.3. Compliance with Study Medication 24](#_Toc61457981)

[8.4. Accountability of the Study Medication 25](#_Toc61457982)

[8.5. Concomitant Medication 25](#_Toc61457983)

[8.6. COVID-19 Vaccination 25](#_Toc61457984)

[8.7. Post-trial Treatment 25](#_Toc61457985)

[9. SAFETY REPORTING 26](#_Toc61457986)

[9.1. Definition of Serious Adverse Events 26](#_Toc61457987)

[9.2. Definitions 26](#_Toc61457988)

[9.3. Causality 27](#_Toc61457989)

[9.4. Procedures for Recording Adverse Events 27](#_Toc61457990)

[9.5. Reporting Procedures for Serious Adverse Events 28](#_Toc61457991)

[9.6. Reporting Procedures for Pregnancy 28](#_Toc61457992)

[9.7. Data Safety and Monitoring Board 28](#_Toc61457993)

[10. STATISTICS AND ANALYSIS 29](#_Toc61457994)

[10.1. Description of Statistical Methods 29](#_Toc61457995)

[10.2. The Number of Participants 29](#_Toc61457996)

[10.3. Analysis of Outcome Measures 29](#_Toc61457997)

[11. DATA MANAGEMENT 30](#_Toc61457998)

[11.1. Access to Data 30](#_Toc61457999)

[11.2. Data Handling and Record Keeping 30](#_Toc61458000)

[12. QUALITY CONTROL AND QUALITY ASSURANCE PROCEDURES 30](#_Toc61458001)

[13. ETHICAL AND REGULATORY CONSIDERATIONS 31](#_Toc61458002)

[13.1. Declaration of Helsinki 31](#_Toc61458003)

[13.2. Guidelines for Good Clinical Practice 31](#_Toc61458004)

[13.3. Approvals 31](#_Toc61458005)

[13.4. Participant Confidentiality 31](#_Toc61458006)

[13.5. Expenses and Benefits 31](#_Toc61458007)

[13.6. Reporting 31](#_Toc61458008)

[13.7. Other Ethical Considerations 32](#_Toc61458009)

[13.8. Community and public engagement 32](#_Toc61458010)

[14. FINANCE AND INSURANCE 32](#_Toc61458011)

[14.1. Funding 32](#_Toc61458012)

[14.2. Insurance 32](#_Toc61458013)

[15. PUBLICATION POLICY 32](#_Toc61458014)

[16. REFERENCES 32](#_Toc61458015)

[17. APPENDIX A: EXAMPLE OF SEVERITY OUTCOME MEASURES 35](#_Toc61458016)

[18. APPENDIX B: SCHEDULE OF STUDY PROCEDURES 36](#_Toc61458017)

[19. APPENDIX C: EXAMPLE OF COMMUNITY AND PARTICIPANT ENGAGEMENT 37](#_Toc61458018)

[20. APPENDIX D: POTENTIAL SITES 39](#_Toc61458019)

[21. APPENDIX E: AMENDMENT HISTORY 41](#_Toc61458020)

# SYNOPSIS

| **Study Title** | Chloroquine/ hydroxychloroquine prevention of coronavirus disease (COVID-19) in the healthcare setting; a randomised, placebo-controlled prophylaxis study (COPCOV) | |
| --- | --- | --- |
| **Protocol number** | VIR20001 | |
| **Study Design** | Randomised double-blind, placebo-controlled trial | |
| **Study Participants** | Healthcare workers and other persons at risk of contracting COVID-19. Adults (exact age is dependent on local country requirements). | |
| **Planned Sample Size** | 40,000 total participants | |
| **Planned Study Period** | 12 months; individual trial duration maximum 5 months | |
|  | **Objectives** | **Outcome Measures** |
| **Primary** | To determine if chloroquine or hydroxychloroquine prophylaxis prevents symptomatic COVID-19 infection in healthcare workers and other persons at risk of contracting COVID-19. | The number of symptomatic COVID-19 infections will be compared between participants randomised to chloroquine or hydroxychloroquine, and placebo groups. |
| **Secondary** | To determine if chloroquine or hydroxychloroquine prophylaxis attenuates COVID-19 infections. | The symptoms severity and duration of COVID-19 illness, in those who become infected during the study will be compared between the two groups using a respiratory severity score. |
|  | To determine if chloroquine or hydroxychloroquine prophylaxis prevents asymptomatic COVID-19 infection. | The number of asymptomatic cases of COVID-19 will be determined by comparing serology in all participants at time of enrolment and at the end of follow up. |
|  | To determine if chloroquine or hydroxychloroquine prophylaxis prevents all-cause symptomatic acute respiratory illnesses. | The number and severity of symptomatic acute respiratory illnesses will be compared in participants randomised to chloroquine or hydroxychloroquine, and placebo groups. |
| **Tertiary** | To characterise genetic and baseline biochemical markers associated with symptomatic COVID-19, respiratory illness and disease severity. | Genetic loci and levels of biochemical components will be correlated with occurrence of and disease severity of COVID-19 or other Acute Respiratory Infections (ARIs). |
|  | To assess the impact of chloroquine or hydroxychloroquine prophylaxis on work and behaviour during the pandemic. | The days lost to work, and the relationship between the subjective assessment of well-being and the decision to self-isolate when unwell (i.e. not go to work) will be examined in relation to the infection and treatment arm. |
|  | To perform health economic analyses to assess the impact of chloroquine or hydroxychloroquine prophylaxis on costs and quality of life measures. | The trial will collect data on use of health care resources and health related quality of life (EQ-5D-3L) to determine the effects between treatment groups. |

# ABBREVIATIONS

| ARI | Acute Respiratory Infection |
| --- | --- |
| COVID-19 | Coronavirus Disease 2019. The disease caused by the virus SARS-CoV-2 |
| CPAP/ BiPAP | Continuous Positive Airway Pressure and Bilevel Positive Airway Pressure |
| CRF | Case Report Form |
| DBS | Dried Blood Spot |
| DSMB | Data Safety and Monitoring Board |
| ECMO | Extracorporeal Membrane Oxygenation |
| ePRO | Electronic Patient Reported Outcomes |
| EQ-5D-3L | EuroQol 5 Dimension 3 levels |
| GCP | Good Clinical Practice |
| ICF | Informed Consent Form |
| LTFU | Lost to follow-up |
| MORU | Mahidol Oxford Tropical Medicine Research Unit |
| OxTREC | Oxford Tropical Research Ethics Committee |
| PI | Principal Investigator |
| PIS | Participant Information Sheet |
| PPE | Personal Protective Equipment |
| RCT | Randomised controlled trial |
| RR | Respiratory Rate. Number of breaths per minute |
| SARS-CoV | Severe acute respiratory syndrome coronavirus |
| SARS-CoV-2 | Severe acute respiratory syndrome coronavirus 2. The virus responsible for COVID-19 |
| SLE | Systemic Lupus Erythematosus |
| SmPC | Summary of product characteristics |
| SOP | Standard Operating Procedure |
| THN | Tropical Health Network |
| URTI | Upper Respiratory Tract Infection (coryza, sore throat) |

# BACKGROUND AND RATIONALE

As of writing, the novel betacoronavirus SARS-CoV-2 has infected more than 80,000,000 individuals, killed more than 1,800,000 people and has spread globally^[[1]](#footnote-2)^. The main mode of transmission of COVID-19 is respiratory droplet spread but spread by direct contact also occurs; with the finding of SARS-CoV-2 in faeces, an oral faecal route of transmission is also possible (1). There is no proven effective prophylaxis and no widely available proven effective antiviral treatment. Corticosteroids are life-saving in hospitalised patients requiring supplemental oxygen (2), and although vaccines have been licensed, they are not yet widely available in some countries, and it will take time to fully vaccinate the world’s population. Chloroquine and hydroxychloroquine have antiviral activity against coronaviruses and are widely available now. Drugs with a modest benefit in preventing COVID-19 could still save many lives in this, and future pandemics. The crude mortality of symptomatic infections is currently greater than 2%. This is some ten times higher than seasonal influenza virus which infects up to 1 billion people a year and kills between 290,000 to 650,000 (3)^[[2]](#footnote-3)^. The estimated COVID-19 basic reproductive ratio (R_0_) of 1.25 to 3.0 is similar to, or slightly higher than, that of seasonal (1.3) or pandemic influenza (1.4 to 1.8) (4, 5). Recently, highly transmissible variants have been selected and spread in some countries.

We have been in a race against time to find effective treatments and preventive measures as the pandemic continues to grow. There is unequivocal evidence that hydroxychloroquine does not benefit patients already hospitalised with COVID-19. At this stage of illness inflammatory processes predominate (and dexamethasone is life-saving). Antiviral drugs are much more likely to be beneficial in prevention or early treatment. The potential role of chloroquine and hydroxychloroquine in the prevention of COVID-19 has been discussed extensively and numerous small, mainly observational, studies have been reported. Meta-analysis of the post-exposure randomised controlled trials (RCTs) are generally in favour of hydroxychloroquine but these are inconclusive. There have been no large RCTs examining the efficacy of 4-aminoquinolines in the prevention of disease, and capable of detecting small, yet clinically significant benefits. The question whether these drugs could be beneficial in preventing COVID-19 remains unanswered. In the absence of a currently widely available vaccine the answer to this question remains important. There remains a major concern that COVID-19 could devastate countries with limited capacity for testing and case isolation, and overwhelm their fragile healthcare systems. The risks to the healthcare system, as seen with SARS-CoV previously, and then in Wuhan and elsewhere with COVID-19, could be a major threat to healthcare operations overall (6).

Chloroquine, an antimalarial drug discovered in 1934 and introduced generally in 1947, is probably the drug to which humans have been most exposed. With an adult treatment dose of 1.5g for malaria, an annual global consumption of hundreds of metric tonnes for over 50 years, and an elimination half-life of approximately one month, the average person in many tropical countries once had detectable chloroquine in their blood. Chloroquine has a very large apparent volume of distribution because of extensive tissue binding and slow elimination (7-9). Plasma concentration profiles with daily dosing are determined mainly by distribution rather than elimination. The main metabolite desethyl chloroquine also has significant biological activity. Chloroquine is inexpensive and simple to administer. It remains a first-line treatment for non-falciparum malaria and is on the World Health Organization’s List of Essential Medicines^[[3]](#footnote-4)^.

Chloroquine has been used extensively as continuous chemoprophylaxis against malaria for individual periods often exceeding five years and has been the prophylactic drug of choice in pregnancy (10). It is safe in all age groups. In addition to its antimalarial use both chloroquine, and the closely related and slightly more hydrophilic hydroxychloroquine, are used in continuous daily dosing for rheumatoid arthritis, systemic and discoid lupus erythematosus and psoriatic arthritis. Chloroquine and hydroxychloroquine at doses of 2.4mg base/kg (155 mg)/day for years is used for rheumatoid arthritis and other conditions. Daily doses up to 620mg base have been given for months or years. Chloroquine given at the correct dose has an excellent safety profile. It has even been added to salt to prevent malaria by mass exposure (11).

Chloroquine has significant antiviral activity against SARS-CoV-2 in cell culture, as it does for the related SARS-CoV (12-15). A half-maximal effective concentration (EC50 or the concentration associated with a decrease in the cytopathic effect of the virus by 50%) of 1.13 µM on Vero E6 cells has been reported with a corresponding EC90 of 6.9 µM. Several other laboratory studies confirm activities in the low micromolar range for chloroquine and hydroxychloroquine (16). This effect occurred when the drug was given either before or after viral inoculation. These are relatively high concentrations by comparison with therapeutic exposures in the treatment of malaria but could be achieved with daily oral dosing. Chloroquine has complex pharmacokinetic properties, having a very large total apparent volume of distribution and a relatively small central compartment with extensive tissue binding, including in the lung. The relationship between plasma concentrations and concentrations in respiratory epithelium is not known precisely, though in rats the concentration in lung is between 124 and 748-fold that in plasma (17). Chloroquine concentrations in the human lung would be expected to exceed those required for the EC90 after an initial dose.

Hydroxychloroquine was synthesised first in 1946 and has largely replaced chloroquine for the management of autoimmune diseases as it has a slightly better adverse effect profile (higher thresholds for toxicity in experimental animals, less abdominal discomfort, higher threshold for retinal toxicity). It has very similar pharmacokinetic properties except for a smaller apparent volume of distribition, probably because of its greater hydrophilicity*. In vitro* it has approximately twice the activity of chloroquine against the SARS-CoV-2 virus in some studies (16). Hydroxychloroquine may cause less itching than chloroquine in dark-skinned patients. The pharmacology appendix contains more information on the pharmacology of these two 4-aminoquinoline drugs.

We hypothesise that chloroquine and hydroxychloroquine might both slow viral replication in exposed participants, attenuating or preventing the infection even if they are shown not to work in treatment or in post-exposure prophylaxis. It is a basic principle of infectious diseases that preventing an infection developing (i.e., preventing pathogen multiplication) requires less drug activity (I.e. lower doses or a less active drug) than treatment. In COVID-19 illness the total viral burden is highest at the time of initial illness.. Viral burdens are often reducing by the time of hospitalisation in COVID-19 so the window of opportunity for antiviral medicines is at the earliest stages of infection. In addition, *in vitro* studies show the greatest activity of chloroquine and hydroxychloroquine at the initial time of cellular infection and decreasing *in vitro* antiviral effects if the drug is exposed at later time points. We believe these drugs may have their greatest utility in preventing COVID-19 in pre-exposure prophylaxis (9, 18-20). Given the enormous experience of use in chemoprophylaxis, excellent safety and tolerability profile and its very low cost, if it proved effective then it would be a readily deployable and affordable preventative measure.

**Main research questions:**

The primary objectives are is to determine if prophylactic chloroquine or hydroxychloroquine prevents symptomatic COVID-19 illness.

The secondary objectives include:

- Attenuation of the clinical severity of COVID-19 infections.
- The prevention of asymptomatic COVID-19.
- The prevention of symptomatic all-cause acute respiratory infections (ARI).

Overview of primary endpoint ascertainment:

| **During the study** | **RT-PCR for SARS-CoV-2** | **Initial serology** | **End serology** | **Diagnosis** |
| --- | --- | --- | --- | --- |
| Illness | Positive | Negative | Negative or Positive | **COVID-19** |
| Illness | Positive | Positive | Negative or Positive | **COVID-19** |
| Illness | Negative | Negative | Positive | **COVID-19**^2^ |
| Illness | Negative | Positive^1^ | Negative or Positive | Indeterminate |
| Illness | Negative | Negative | Negative | ARI not COVID-19 |
| No Illness | - | Negative | Positive | Asymptomatic case |
| No Illness | - | Positive^1^ | Positive | Indeterminate |
| No Illness | - | Negative | Negative | Not infected |

^1^Participants will be enrolled only if they have not had a previous confirmed diagnosis of COVID-19. It is anticipated some participants may have had asymptomatic infections and so are found during subsequent analysis to have antibody present at study enrolment.

^2^Unless the PCR from a nose/ throat swab taken during the febrile illness by the hospital is positive for influenza virus. Other viruses isolated will not change the primary end-point determination. Although later studies will look for other viruses in the study swab – for the primary endpoint this will only be a SARS-CoV-2 PCR.

Brief description of the intervention:

The study is a double-blind, randomised, placebo-controlled trial that will be conducted in facilities involved in COVID-19 case management. After obtaining fully informed consent, we will recruit healthcare workers and other persons at risk of contracting COVID-19, who can be followed up reliably for up to 5 months.

A loading dose of 10 mg base/kg (four 155mg tablets for a 60kg participant), followed by 155 mg daily (250mg chloroquine phosphate salt or 200mg of or hydroxychloroquine sulphate) will be taken by all participants for 3 months. Subsequent episodes of symptomatic respiratory illness, including symptomatic COVID-19, clinical outcomes, and asymptomatic infection with the virus causing COVID-19, will be recorded during the follow-up period. If participants are diagnosed with COVID-19 during the period of prophylaxis, they will continue their prophylaxis unless advised to discontinue by their healthcare professional or they become hospitalised as a result of COVID-19 (not for quarantine reasons), in which case they will be asked to stop. If they are hospitalised prior to the diagnosis of COVID-19, they will continue prophylaxis until the diagnosis of COVID-19 is confirmed unless advised to discontinue by their healthcare professional.

If participants become unwell during the study period due to COVID-19 or other ARI, they will continue to be followed up until 28 days from the beginning of illness. If complete recovery does not occur within 28 days, follow-up will be extended for up to a maximum of 60 days from the beginning of illness. For participants who become unwell on Day 90, follow-up may therefore continue until Day 150.

**Investigation of a suspected case:**

The procedures for identifying a case and the subsequent isolation and management will follow local and national guidelines; this study will not interfere in the usual local investigation and management of COVID-19 cases. Study diagnoses will be made at the end of the study, where possible participants will be informed if they had or did not have COVID-19 infections as defined above. Chloroquine and hydroxychloroquine have very few drug-drug interactions and should not interfere with the management of pneumonia.

**Summary of findings of previous studies:**

No large randomised controlled studies have published results on chloroquine or hydroxychloroquine for the pre-exposure prophylaxis of COVID-19 in humans and there remains clinical equipoise as to whether the drug will have a clinically significant benefit in this indication. Studies of these drugs in late treatment suggest they are not life-saving, but no antiviral has proven so, and the benefit of an anti-inflammatory drug, dexamethasone, at this late stage suggests that at this point it is too late for an antiviral, when inflammation predominates (21, 22). The drugs are more likely to be beneficial if given earlier, when the virus is replicating and the available, pooled RCT data are suggestive of a modest benefit at this earlier stage (23).

However, chloroquine has been used widely and a wealth of experience and data testify to its safety both for mass drug administration (MDA) for malaria, as routinely prescribed antimalarial prophylaxis, and for rheumatological conditions for which people may be take the drug daily at doses comparable to those in this study for decades with few ill-effects. Hydroxychloroquine has been used widely for over 50 years in the treatment of rheumatoid arthritis, SLE and other similar conditions.

The risks of chloroquine or hydroxychloroquine chemoprophylaxis are minimal compared with the risks of COVID-19 and there are currently no other proven chemoprophylactic agents, widely available effective treatments, other than steroids in those requiring supplemental oxygen, or a widely-available vaccine in all countries. Assumptions of the study include that the *in vitro* effects of chloroquine or hydroxychloroquine against SARS-CoV-2 will translate to an *in vivo* effect and a benefit in human participants. As described, chloroquine or hydroxychloroquine should reach levels in human tissues, including the lungs, which were shown to have a viral suppressive effect *in vitro*. However, the exact distribution of chloroquine or hydroxychloroquine within the respiratory tract, and whether these *in vitro* findings will translate into clinical benefit, is unknown.

**Summary of known and potential risks and benefits of the study:**

**Risks**

Risks related to chloroquine phosphate/ sulphate/ hydrochloride and hydroxychloroquine sulphate are very low, unless the drug is taken in overdose. These are very safe and generally well-tolerated medications but adverse reactions relating to the cardiovascular system, the central nervous system, the skin, hypoglycaemia, hypersensitivity, gastrointestinal, and retinal toxicity have all been described though usually after high doses or protracted exposures. Headache and gastrointestinal side-effects (e.g. nausea, diarrhoea) are the most common adverse effects (24). Another adverse effect is itching, in particular with chloroquine, in dark-skinned individuals; Africans are much more commonly affected compared to Asians. These risks will be mitigated by excluding participation if people have had a previous serious adverse reaction to chloroquine, or hydroxychloroquine, 4-aminoquinoline compounds, any components of the tablet or retinal or visual field changes of any aetiology.

Despite an excellent track record of safety with over 70 years of use of these drugs, during COVID-19 there has been much written about the potential cardiotoxicity of these drugs. Many of these reports did not consider dose (COPCOV uses the lowest range of the daily dose used safely for long periods of time without need for ECG monitoring or concerns), the cardiotoxicity seen with chronic use (as opposed to the short period of the COPCOV study), do not distinguish use of the drugs alone (safe) from the potentially cardiotoxic combination with azithromycin, and ignore the wealth of clinical and epidemiological data suggesting the excellent safety of these drugs. The mild QT prolonging effect of hydroxychloroquine and chloroquine has been well-known and has not been associated with a significant risk of ventricular tachyarrhythmias such as torsade de pointes. The WHO pharmacovigilance database (VigiBase) contains reports of 83 episodes of TdP or other forms of ventricular tachycardia which were associated with hydroxychloroquine over a 52 year period, of which 7 were fatal. This experience does not distinguish acute from chronic use and most pertains to diseases with an increased risk of cardiac disease. This should be viewed in the context of approximately one million people using hydroxychloroquine continuously worldwide (based on manufacturing outputs).

Despite the opinion pieces, case reports and uncontrolled observational studies about safety concerns of these drugs, the high-quality randomised controlled trial evidence from COVID-19 is reassuring. The RECOVERY and SOLIDARITY studies, despite using much higher doses in a sick population (approximately 4 times higher than the COPCOV study) showed no excess of cardiac arrhythmias (21, 22). In addition, data from outpatient RCTs using lower doses also support the safety of these drugs. In contrast to reported observational data these RCTs do control adequately for confounders of the disease and its treatment. Of 2795 participants recruited into 3 RCTs using HCQ in the outpatient setting, 1633 received HCQ and the study concluded that “gastrointestinal side effects were common but mild with the use of hydroxychloroquine, while serious side effects were rare. No deaths occurred related to hydroxychloroquine” (24). Together these data suggest that the safety and tolerability profile of chloroquine and hydroxychloroquine in COVID-19 is similar to that in rheumatological conditions. Finally, high-quality, controlled observational data of participants taking hydroxychloroquine for rheumatological conditions confirm the safety and suggest the drugs may be antiarrhythmic in the short-term. In a recent retrospective observational review of 956,374 rheumatoid arthritis patients starting treatment with hydroxychloroquine, there was a lower risk of arrhythmia in the first 30 days of treatment (calibrated hazard ratio (CalHR) 0.89 (95% confidence interval 0.77 to 1.04) compared with sulphasalazine recipients (n = 310; 350) (25). The levels of drug in the blood have been modelled based on available data, and data from deliberate overdose, and are well below the threshold for toxicity (26, 27).

In conclusion, the evidence from clinical trials in COVID-19 support the well-established safety record of the 4-aminoquinolines (chloroquine and hydroxychloroquine) at the correct dosage.

A full description for each product is provided in the relevant summary of product characteristics (SmPC). A physician’s guidance document is also available for reference.

**Benefits**

- Access to a drug which may potentially prevent or ameliorate COVID-19 infection. No other proven preventive medication or widely-available vaccine around the globe currently exists. The main potential benefit is to the participant in the chloroquine or hydroxychloroquine arm (direct protection) but individuals in the placebo arm may benefit from indirect protection through decreased ability of the infection to spread.
- Awareness that their participation may lead to an intervention which may save many lives around the world or, alternatively, may show chloroquine or hydroxychloroquine prophylaxis is ineffective so trials can move on to evaluate other possible interventions with a minimum of delay, and the prophylactic use of these drugs around the world can stop.

**Description of the population to be studied and the population to whom the results of the study may be generalisable:**

The population to be studied comprises adult healthcare workers and other persons at risk of contracting COVID-19. These could include nurses, healthcare assistants (HCAs), doctors, pharmacists, physiotherapists, porters and anyone who is at risk of exposure to COVID-19.

If shown to be beneficial, this study would be generalisable to all people around the world at risk of COVID-19.

# OBJECTIVES AND OUTCOME MEASURES

| **Objectives** | **Outcome measures** | **Timepoint(s) of evaluation of this outcome measure (if applicable)** |
| --- | --- | --- |
| **Primary Objective** To determine if chloroquine or hydroxychloroquine prophylaxis prevents symptomatic COVID-19 infection in healthcare workers and other persons at risk of contracting COVID-19. | **Primary Outcome**  The number of symptomatic COVID-19 infections will be compared between participants randomised to chloroquine or hydroxychloroquine, and placebo groups. | During the trial period |
| **Secondary Objectives**  To determine if chloroquine or hydroxychloroquine prophylaxis attenuates COVID-19 infections. | **Secondary Outcomes**  The symptoms, severity and duration of COVID-19, in those who become infected during the study will be compared between the two groups using a respiratory severity score. | During the trial period |
| To determine if chloroquine or hydroxychloroquine prophylaxis prevents asymptomatic COVID-19 infection. | The number of asymptomatic cases of COVID-19 will be determined by comparing serology in all participants at time of enrolment and at the end of follow up. | During the trial period |
| To determine if chloroquine or hydroxychloroquine prophylaxis prevents all-cause symptomatic acute respiratory illnesses. | The number and severity of symptomatic acute respiratory illnesses will be compared between the chloroquine or hydroxychloroquine, and placebo groups. | During the trial period |
| **Tertiary Objectives**  To characterise genetic and baseline biochemical markers associated with symptomatic COVID-19, respiratory illness and disease severity. | **Tertiary Outcomes**  Genetic loci and levels of biochemical components will be correlated with occurrence of and disease severity of COVID-19 or other ARIs. | During and after the trial period |
| To assess the impact of chloroquine or hydroxychloroquine prophylaxis on work and behaviour during the pandemic. | The days lost to work, and the relationship between the subjective assessment of well-being and the decision to self-isolate when unwell (i.e. not go to work) will be examined in relation to infection and treatment arm. | During and after the trial period (to the limit of follow up) |
| To perform health economic analyses to assess the impact of chloroquine or hydroxychloroquine prophylaxis on costs and quality of life measures. | The trial will collect data on use of health care resources and health related quality of life (EQ-5D-3L) to determine the effects between treatment groups. | During and after the trial period |

Additional exploratory objectives will be defined prior to study analysis, e.g., pharmacokinetic (PK) analysis of study medication levels, or potential attenuation of antibody levels by study drug in vaccinated participants

# STUDY DESIGN

The study is a double-blind, randomised, placebo-controlled trial that will be conducted primarily in healthcare settings and other facilities directly involved in COVID-19 case management. We will recruit healthcare workers and other persons at risk of contracting COVID-19, who can be followed reliably for 5 months. 40,000 participants will be recruited and we predict an average of 400-800 participants per site in 50-100 sites.

Before the trial enrolment starts there will be engagement with the potential participants to inform them about the trial and possibly obtain baseline demographic information from potential participants. Eligible participants will give written informed consent. As part of the informed consent process the risks and benefits of the study will be explained to them in their language, including potential side-effects of chloroquine and hydroxychloroquine. They will also be informed that biological samples will be stored and may be processed for genetic material, biochemical tests, and other pathogens. They will also consent to having clinical information shared with the study team, although these data will remain pseudonymised and stored and processed in accordance with national and international standards and in accordance with regulating bodies. The participant will be instructed how to contact the study team and how to use the simple reporting application (app) on their mobile phone. While well, the participant will continue their normal duties and activities (e.g. in the healthcare facility). The study procedure of reporting side-effects and adverse reactions will be explained (reporting to the site local PI and if necessary, stopping the medication). The participant will also be informed what to do if they develop symptoms of an acute respiratory infection (ARI), which will be to alert the study team and follow institutional and governmental guidelines to get tested for COVID-19 (dependent on site).

The participant will be randomised to receive either chloroquine or placebo (1:1 randomisation), or to hydroxychloroquine or placebo (1:1 randomisation). A loading dose of 10mg base/kg (four 155mg tablets for a 60kg subject), followed by 155 mg daily (250mg chloroquine phosphate salt/ 200mg hydroxychloroquine sulphate) will be taken for 3 months.

If the participant is diagnosed with COVID-19, they will take continue to take the study medication until:

- 90 days after enrolment (i.e., completion of kit)
- hospitalised due to COVID-19 disease (i.e., not for quarantine purposes) in which case they will stop, or
- advised to stop by their healthcare professional for other reasons

If participants are hospitalised with symptoms consistent with COVID-19, they will continue the study medication until they are formally diagnosed with COVID-19 or unless advised to stop by their healthcare professional. If the participant misses a dose, they can take this dose later, up until the time they would take their next daily dose. If they do not take their dose within this period of time, they will not take it and this dose will be classified as missed. They should continue to take their medication regularly. The missed dose will be reported to the study team via the mobile app and at the subsequent follow up visit at the study site.

Episodes of symptomatic respiratory illness, including symptomatic COVID-19, and clinical outcomes will be recorded in the CRF during the follow-up period.

At the initial visit participants will provide demographic and basic clinical data and have their weight and height measured. 10mls of blood will be taken, centrifuged and the serum, plasma and cell fraction stored at -80°C for future analysis. This sample will be used for baseline antibody testing, chloroquine/ hydroxychloroquine levels, biochemical tests and host genetics related to susceptibility to respiratory illness and COVID-19 infection and chloroquine/ hydroxychloroquine levels.

Participants will be given a participant ID number (a card in most settings), randomised and given 30 days of study medication and asked to see the local PI or study nurse 28-30 days later. The drug will be taken once daily in the morning (or evening for night shifts). The card will have contact numbers for the study team members whom they are to inform should they develop adverse reactions/ side-effects or symptoms. The initial weight-based loading dose will be observed by the study nurse. Participants will also be given a thermometer, and requested to record their temperature twice a day, as well as any significant exposures or symptoms.

Participants will be prompted to record twice daily temperature readings and symptoms via a mobile-based application or web interface. The data will be transferred securely to the team and merged with other study data for analysis. Participants who do not record symptoms and temperature readings at minimum daily will be contacted within 24-48 hours by the study team. Participants who report being unwell will be further assessed by the study team. Should participants be unable to access the mobile application or website, the study team will phone them and record the data on their behalf.

If symptoms consistent with COVID-19 occur, the participant will alert the study team and will arrange for nose and throat swab samples (even if a sample has been taken previously for clinical purposes) following strict adherence to personal protection. In some instances, a sputum sample may also be taken. The participant should continue his/ her chloroquine/ hydroxychloroquine or placebo, unless otherwise advised by a medical professional or the study team, or they are diagnosed with COVID-19 and hospitalised with infection (not for quarantine purposes). Prescribing medical professionals should be mindful that participants may or may not be taking chloroquine/ hydroxychloroquine and in some cases, determined per site specific guidance, this may necessitate unblinding.

If there is a subsequent significant clinical change in the participant or the participant has further episodes of ARI within the trial period, this process will be repeated. Samples will be stored at -80°C and tested for respiratory viruses at the end of the trial. The participant will self-isolate, as per local or national guidelines. If a clinical sample has been taken for local analysis and is negative for COVID-19, then self-isolation can stop according to local or national guidance.

If a diagnosis of COVID-19 from a clinical sample is confirmed then the isolation practices and contact tracing will follow the local practices and guidelines, and chloroquine/ placebo or hydroxychloroquine/ placebo will continue unless the participant is advised to stop by their healthcare professional or they become hospitalised with infection (not for quarantine purposes). The participant will continue to give an update of their clinical condition on the app, or will be called by mobile phone until recovered and followed up once more at 28 days by phone. If the participant develops an ARI within the final 60 days of the study which is not diagnosed as COVID-19, they should continue chloroquine or hydroxychloroquine/ placebo as normal (unless advised by a medical professional or the study team) but will be followed up for 28 days after the onset of infection. For all participants with an ARI, including those confirmed to have COVID-19, if the participant has not recovered by 28 days this period can be extended up to 60 days.

As well as twice daily electronic reporting, participants will be reviewed by the study team at least monthly to assess drug tolerability, well-being, respiratory and other symptoms and fever, and whether the local authorities have taken a swab for COVID-19 (in case they had not contacted the study team). This will be done in person (if the participant is not symptomatic; if symptomatic separate provisions will be arranged) and will be combined with:

- Collection of a study adverse events questionnaire;
- A dried blood spot (DBS) sample on filter paper for hydroxychloroquine/ chloroquine levels +/- COVID-19 diagnostic tests;
- Dispensation of further study drugs.

Participants will be requested to give a further 5ml clotted blood sample at the end of the trial. They will be asked not to take their trial medication on the morning of review the medication will be taken after the interview.

Participants who are offered a COVID-19 vaccine prior to D90 should inform the study team at the earliest possible opportunity. Prior to vaccination, or up to 3 days after first dose of COVID-19 vaccine, the study team will collect 5ml of clotted blood and DBS. The study team will advise the participant to stop taking the study medication but will confirm the participant will continue in the study for remote follow up through D90. Vaccinated participants will then be asked to give a further venous blood sample of 5mls at 28 days (+/- 3 days) after first dose of vaccine, in order to determine whether antibody responses differ between those who are on active drug or placebo.

For those who develop symptomatic COVID-19 illness or ARI, a continuous severity score will be used to assess severity, and these will be captured longitudinally over time. In order to discriminate between severity at the lower end of the spectrum we will use a logarithmic scale and a Wilcoxon test can then be used to compare ranks between the two groups.

Participants will remain enrolled until one of the following events occur:

- The trial ends
- They choose to withdraw consent or no-longer wish to participate in the trial
- An adverse event warrants removal from the study
- Discontinued by the investigator

Participants who discontinue study medication early will be encouraged to complete all other study assessments through Day 90.

# PARTICIPANT IDENTIFICATION AND RECRUITMENT

## Study Participants

The study population is adult healthcare workers and other persons defined by the site investigator at risk of contracting COVID-19.

## Inclusion Criteria

1. Participant is willing and able to give informed consent for participation in the study and agrees with the study and its conduct
2. Agrees not to self-medicate with chloroquine, hydroxychloroquine or other potential antivirals
3. Adults (exact age is dependent on countries) less than 70 years old at the time of consent
4. Not previously diagnosed with COVID-19
5. Not currently symptomatic with an ARI
6. Participant is a healthcare worker or is a person at risk of contracting COVID-19.
7. Possesses an internet-enabled smartphone (Android or iOS)

## Exclusion Criteria

The participant may not enter the study if ANY of the following apply:

1. Hypersensitivity reaction to chloroquine, hydroxychloroquine or 4-aminoquinolines
2. Contraindication to taking chloroquine as prophylaxis e.g. known epileptic, known creatinine clearance < 10 ml/min
3. Already taking chloroquine, hydroxychloroquine or 4-aminoquinolines, or history of these medications within the previous 7 days
4. Taking a concomitant medication described in Section 8.5
5. Known retinal disease
6. Inability to be followed up for the trial period
7. Known prolonged QT syndrome (however ECG is not required at baseline)
8. Known pregnancy or women who are actively trying to become pregnant
9. Prior diagnosis of porphyria
10. Previously received any dose of COVID-19 vaccine

The investigator may consult the physician’s guidance documents for any further questions regarding eligibility of potential participants.

# STUDY PROCEDURES

## Recruitment

Study sites will be initially pre-selected on the following criteria if ALL of the following are met:

- There is local agreement that the study can be conducted in the facility
- Local or national ethical/ IRB approval can be put in place rapidly
- It is a facility where there are cases of either proven or suspected COVID-19 and/or the site investigator defines other potential participants at risk of COVID-19 infection
- There are adequately trained personnel able to conduct the study procedures described in the protocol and appropriate equipment
- Each site would be able to recruit a projected 400 participants during the trial period (200 participants per site may be possible on discussion)

Study sites *may* then be selected if ANY of the following criteria are met:

• Confirmed nosocomial spread of COVID-19 in the healthcare facility, or neighbouring facilities

• Confirmed cases of COVID-19 in the healthcare facility, or neighbouring facilities

• Confirmed person-to-person transmission of COVID-19 in the local area

Recruitment of individuals into the study once sites are confirmed and local or national ethical/ IRB approval is in place:

Facilities will contact their staff or other persons locally defined at risk of contracting COVID-19 to inform them of the study through usual means. In addition, with the local ethics committee approval and institution’s consent, the site study PI may advertise the study with posters, social networking and through word of mouth. Recruitment into the study will occur in person either in, or nearby, the facility.

## Screening and Eligibility Assessment

Eligibility assessment will occur at the point of screening. If, based on the inclusion and exclusion criteria, the participant is eligible, they will be randomised to receive chloroquine / hydroxychloroquine or placebo.

## Informed Consent

The participant must personally sign and date the latest approved version of the Informed Consent form before any study specific procedures are performed.

Written and verbal versions of the Participant Information and Informed Consent will be presented to the participants detailing no less than: the exact nature of the study; what it will involve for the participant; the implications and constraints of the protocol; the known side effects and any risks involved in taking part; their samples being stored and being processed for host genetic material and other pathogens; and for any clinical and other personal data during the trial, being shared with the study team.

It will be clearly stated that the participant is free to withdraw from the study at any time for any reason without prejudice to future care, and with no obligation to give the reason for withdrawal.

The participant will be allowed as much time as wished to consider the information, and the opportunity to question the Investigator or other independent parties to decide whether they will participate in the study. Written Informed Consent will then be obtained by means of participant dated signature and dated signature of the person who presented and obtained the Informed Consent. The person who obtained the consent must be suitably qualified and experienced, and have been authorised to do so by one of the Investigators. A copy of the signed Informed Consent will be given to the participant. The original signed form will be retained at the study site.

## Clinical examination

There will be no physical clinical examination. Basic demographic information, and details of past medical history, concomitant medications, allergies, smoking and other drug intake will be noted. The height, weight, and temperature will be recorded.

## Randomisation and blinding

Chloroquine phosphate tablets containing 155mg base equivalent and identical placebo pills will be packed in opaque blister packs containing 10 tablets. Hydroxychloroquine sulphate tablets containing 155mg base equivalent and identical placebo pills will also be packed in opaque blister packs containing 10 tablets. Each participant will receive a study box containing up to 100 tablets. The initial dose to be taken from the starter blister pack is 10mg/kg, which will be between three and five tablets depending on the weight of the participant.

A randomisation list will be prepared by a statistician using block randomisation in a 1:1 ratio for the chloroquine/ hydroxychloroquine arm versus the placebo and stratified by site. The randomisation will be computer-generated and programmed in Stata 15. An appropriate computer seed will be used to allow reproducibility of the randomisation list. The list will be provided directly to the pharmaceutical company by the trial statistician to allocate a drug kit containing 10 blister packs with 10 tablets in each blister to participants based on the computer pre-generated randomisation list. The packaging of study drug kits will be performed by independent staff at the pharmaceutical company and will follow the computer-generated randomisation list provided by the statistician. Should the company not have capacity to pack treatment, the packaging of study drug kits will be performed by independent staff at MORU. At enrolment the participant’s kit number/ ID will be written on the study drug kit and on each blister pack (only if possible), and the starter blister pack and first 30 tablets dispensed to the participant. The kit number/ ID will be linked to the treatment that has been allocated to each participant. All study team members will be blinded to the actual treatment and only the trial statistician and the backup statistician will have access to the randomisation code. The unblinded randomisation list and the randomisation programs will be securely kept by the statistician and backed-up. The study drug kits will be kept securely by the study team. Subsequent 30 tablets (3 blister packs) will be dispensed at each monthly check with the study team. Individual unblinding will be done only on consultation with the study PI. Only the study statistician will have the drug allocation list.

A separate procedure will be provided to study teams to describe details of randomisation and study medication management.

## Baseline Assessments

At Day 0 (D0), participants will be given a card with a participant ID number, have an app installed on their mobile phone, be randomised and given 30 days of study medication, and asked to see the local PI 28-30 days later. The study medication will be taken in the morning (or evening for night shifts). The card will have contact numbers for the study team members whom they are to inform should they develop adverse reactions, side-effects or symptoms.

Participants will also be given a thermometer, will be requested to record their temperature twice a day, as well as any significant exposures or symptoms on an app (phone-based) reporting software application. The mobile app will be set up on the participant’s phone and they will be instructed in its use in the presence of the study team at D0, as well as instructing them on how to report symptoms and use the thermometer. Those reporting to be unwell or those who do not respond on the app will be contacted by the study team.

At the initial visit participants will provide demographic and basic clinical data, including co-morbidities and concomitant medications, information on well-being, and have their weight and height measured. 10mls of blood will be taken and two blood spots will be collected on filter paper for baseline chloroquine/ hydroxychloroquine levels. The serum, plasma and cell fraction will be stored at minus 80°C for future analysis.

The participant will be observed taking the first (weight-based dose) dose of study drug by the study team.

## Subsequent Visits

At Day 30, if the participant is asymptomatic they will present in person to collect a further 30 days of study medication. This process will occur every 30 days for a total 3 months. If the participant does not present, they will be contacted and the appointment will be arranged, and provision made for the participant to collect the study product.

The expected schedule for follow up visits is every 28-30 days based from Day 0, a visit window of 27-31 days is allowed to accommodate participant scheduling during study conduct. The final follow up visit (D90) has a visit window from D87-D97 to ensure data for important study endpoints are collected.

At each visit (D30, D60 and D90):

- Participant identification will be confirmed. Use of the mobile telephone number given at the initial assessment will suffice as long as the study team has no reason to suspect the participant is not the same person.
- Adherence will be reviewed via question and pill count. The used blister pack will be returned, checked and stored. The time of the last dose will be noted.
- Well-being, adverse reactions or side-effects will be assessed.
- Symptoms compatible with COVID-19, testing for and results of testing for the infection.
- A finger prick for 2 blood spots will be performed on a filter paper.
- At the final visit (D90), 5ml of venous blood will be taken in a clotted bottle and 2 blood spots will be collected on a filter paper.

Additionally, during this period the study participant will be asked to record entries twice daily via a mobile phone app. They will be reminded to take their tablet. These data will be transferred securely to the team and analysed. Those reporting to be unwell or those who do not respond on the mobile app will be contacted by the study team.

If via the app or by phone, the participant reports to feel unwell with an ARI (potential COVID-19 symptoms) or potential drug side-effects, they will be contacted by the study team. The study team will ask the participant further questions and if required, within 48 hours a nose and throat swab will be organised according to study SOP. Further follow-up of initial unwellness by the study team would be a the discretion of the assessing physician, however participants should continue to report data via the app. If a significant clinical deterioration should occur, or further ARIs occur in the study period, the nose and throat swab will be repeated. If the participant is producing sputum, a sample will be collected in a pot, or a sputum pot will be left with the participant, for later collection.

The participant will be advised to inform their healthcare professional that they are in the study along with the study medications they may be taking. If they are hospitalised with confirmed COVID-19 as a result of the infection (not for quarantine purposes), the study medication should be stopped. If the participant’s healthcare professional starts a treatment which is known to prolong the QT interval, while the participant is enrolled in the study, then an ECG should be performed by this professional and checked for QT prolongation.

If the participant is offered a COVID-19 vaccine prior to D90, the study team will:

- Advise immediate discontinuation and return of study medication.
- If possible to obtain prior to vaccination or up to 3 days after first vaccine dose, collect 5ml of venous blood in a clotted bottle and 2 blood spots via a filter paper.
- Collect a further 5ml of clotted blood 28 days (+/-3 days) after first vaccination.
- Confirm the participant will agree to continue other data entry and monitoring through D90 or 28 days after first vaccination, whichever comes later.
- The team will continue to follow up reports of potential ARIs or other illnesses, including collection of a swab where indicated.

## Sample Handling

On D0, 10mls of venous blood will be taken [EDTA (4mL) and clotted bottle (6mL)]. Both samples will be centrifuged at 1500g for 10 minutes. Three aliquots of serum from the clotted bottle and three aliquots of plasma from the EDTA tube will then be stored at minus 80°C until further notice. Additionally, a single aliquot of the cell fraction from the EDTA tube will also be aliquoted and stored at minus 80°C. Two spots (50 µL each) of blood will be collected on a filter paper and stored in an individual small plastic bag with a desiccant sachet.

At D30 and D60 of the trial, when participants attend for review, finger prick will be performed to collect 2 blood spots on the filter paper.

On the last day of the trial 5mL of venous blood will be collected in a clotted bottle, which will subsequently be centrifuged and aliquoted to 3 cryovials as described above. Two blood spots will also be collected on a filter paper.

Nose and throat swabs will be taken in accordance with the provided study SOP and frozen immediately at minus 80°C in their collection tube, or handled per local procedure if agreed with MORU team. Sputum will either be frozen in the container in which it is collected (if amenable to freezing) at minus 80°C or the sample will be transferred to a viral swab and stored at minus 80°C.

Samples will be transferred to MORU Tropical Health Network laboratories or designated testing facilities in other regions, where they will undergo testing in accordance with best practice laboratory measures and safety procedures.

Validated antibody tests for SARS-CoV-2 are currently being developed rapidly. The plasma and serum aliquot samples will be stored until a time that validated assays for these have been developed or we have completed our own in-house serological tests and validations. The criteria for a positive test are thus yet to be determined, but as with other serological tests, a four-fold increase in titre of SARS-CoV-2 antibodies between the initial and final sample will likely be used to determine exposure to the virus. Additionally, we will be able to determine if exposure to SARS-CoV-2 has occurred prior to enrolment in the trial if the initial antibody titre is above a predetermined and validated level. Serological tests for other circulating coronaviruses may also be performed to determine the interaction of these with COVID-19, as well as other pathogens which may be of clinical significance.

Testing of the serum samples for other biological parameters which may impact susceptibility to infection, such as ACE2, zinc and vitamin D levels, may also be considered at a later date.

Nose and throat swabs will be processed using validated multiplex RT-PCR to detect SARS-CoV-2 as well as other respiratory viruses including some or all of the following: influenza A, influenza B, respiratory syncytial virus, rhinovirus, other coronaviruses (OC43, NL63, 229E and HKU1), metapneumovirus, parainfluenza 1-4, adenovirus and bocavirus. The cycle threshold (CT) value of positive results will be recorded.

The cell fraction aliquot will be processed to assess for host genetic markers of respiratory disease susceptibility. These tests may be done in Thailand or elsewhere in Asia, the UK or Europe, once material transfer agreements are in place.

DBS samples will be used for measurement for chloroquine/ hydroxychloroquine levels and may be used for diagnostic tests for COVID-19 (antibody, antigen or PCR).

The samples will be retained per Oxford and local site regulations. Consenting participants may rescind their consent at a later date and refuse the use of their samples (which will be destroyed) or data at any time up until the completion of the study.

## Discontinuation/Withdrawal of Participants from Study

Each participant has the right to withdraw from the study at any time. In addition, the Investigator may discontinue a participant from the study at any time if the Investigator considers it necessary for any reason including:

- Ineligibility (either arising during the study or retrospectively having been overlooked at screening)
- Significant protocol deviation
- Significant non-compliance with treatment regimen or study requirements
- Withdrawal of Consent
- Loss to follow up (LTFU)

Participants who discontinue study medication early, e.g. due to COVID-19 vaccination or pregnancy, will be asked to remain in the study to complete all other defined study procedures.

The reason for withdrawal will be recorded in the Case Report Form.

Participants who withdraw or are removed from the study will not be replaced.

## Definition of End of Study

The end of the study will be the date of the last visit of the last participant, the last dose of the study drug or up to 60 days after the diagnosis of COVID-19/ ARI of the last participant enrolled in the study, whichever comes last.

# STUDY MEDICATION (CHLOROQUINE or HYDROXYCHLOROQUINE/PLACEBO)

## Study Medication Description

The trial intervention is the administration of the study product. This will either be chloroquine or placebo, or hydroxychloroquine or placebo. It is expected that chloroquine will be used in Asian sites and hydroxychloroquine in Europe, specific drug allocation will be determined by country prior to activation based upon factors such as inventory availability and importation requirements.

Characteristics of each product are described in the SmPC.

Chloroquine and hydroxychloroquine will be in the dose of 155mg chloroquine base (250mg of chloroquine phosphate or 200mg of hydroxychloroquine sulphate). On D0 the participant will be supervised taking 10mg base/kg by the study team (usually 3-5 tablets depending on weight; where the dose is split then only the initial part of this dose will be observed) and they will be given a further 30 tablets of 155mg base to be taken once daily. The placebo will comprise identical tablets and the regimen will be the same with 1 tablet/ 15kg at D0 and a further 30 tablets to be taken once daily. Neither the participant, nor those conducting the study will know if the participant is receiving chloroquine/ hydroxychloroquine or placebo.

## Storage of Study Medication

The medication will be stored securely per manufacturer instructions in the institution’s pharmacy or other secure location. The medication will only be accessible to the designated study team members.

## Compliance with Study Medication

Adherence will be assessed by direct questioning of the participant. Participants will receive reminders to take the medication from the app. The monthly pre-dose capillary blood chloroquine or hydroxychloroquine measurement will be an independent measure of exposure. Given that the study will be conducted on healthcare workers or other participants potentially at higher risk of infection, and the current concern relating to COVID-19 is so great, we do not anticipate poor adherence. In the event of lost medication or more than 3 consecutive missed doses the participant should contact the study team, if they have not already been contacted by the study team.

## Accountability of the Study Medication

The medication and placebo supplies will be supervised at all times by study teams. Medication counts will occur to ensure that no tablets are missing. Dispensation and return of study drugs will be recorded in the Study Drug Accountability Log.

## Concomitant Medication

Chloroquine or hydroxychloroquine must be avoided if the participant is taking the following medications:

**Antiarrhythmic medications**: digoxin, amiodarone, sotalol, flecainide

**Antiparasitic/malarial agents:** mefloquine, halofantrine, praziquantel

**Antibiotics:** levofloxacin, moxifloxacin, ciprofloxacin, azithromycin, clarithromycin, erythromycin

**Antifungal drugs:** fluconazole, ketoconazole, itraconazole, terfenadine

**Psychoactive drugs**: lithium, quetiapine, chlorpromazine, thioridazine, ziprasidone, haloperidol, droperidol, methadone

**Migraine treatment**: sumatriptan

**Antihistamines**: astemizole

**Antiemetics**: prochlorperazine, metoclopramide

**Cancer treatments**: abiraterone, dabrafenib, dacomitinib, enzalutamide, idelalisib, mitotane

**Other specific drugs:** ciclosporin, conivaptan, agalsidase alfa or beta, mifepristone, stiripentol

PIs will also be directed to **crediblemeds.org** to check other agents that may prolong QT interval

## COVID-19 Vaccination

The study team have carefully considered how the development and planned global roll out of COVID-19 vaccines may impact upon the primary study outcomes. Consequently, anyone who receives a COVID-19 vaccination will not be eligible for enrolment in the COPCOV study and those participants who are vaccinated during the study period will stop the study medication but will remain in the study and be followed through D90, or 28 days after the 1^st^ dose of vaccine, whichever comes later. The study will not interfere with participants being vaccinated. The participant will be asked to provide a venous blood and DBS sample (as would normally be obtained at D90), for ascertainment of the study’s endpoints. In addition, participants vaccinated during the study will have a further venous blood test drawn at 28 days (+/-3 days) after the first vaccine dose to determine if the study medication has any effect on the antibody response. We foresee that we may collect additional AEs and SAEs not related to the study medication, but caused by the vaccine; however we consider it is important to determine if there are any interactions between the study drugs and the COVID-19 vaccines.

It is hoped that all participants in the COPCOV study will eventually receive one of the COVID-19 vaccines. It is important therefore to ensure that there are no untoward effects in hydroxychloroquine or chloroquine recipients should they receive vaccine during the study. Drug administration will stop and the efficacy endpoint censored at the time of vaccination, but adverse effects will continue to be monitored through final assessment. . Blood samples will be drawn at the time of and 28 days post-vaccination, to evaluate that the drugs do not attenuate antibody responses.

## Post-trial Treatment

We are currently not planning to provide the chloroquine or hydroxychloroquine post-trial. They are readily available and affordable.

# SAFETY REPORTING

## Definition of Serious Adverse Events

A serious adverse event is any untoward medical occurrence that:

- results in death
- is life-threatening^[[4]](#footnote-5)^
- requires inpatient hospitalisation^[[5]](#footnote-6)^ or prolongation of existing hospitalisation
- results in persistent or significant disability/ incapacity
- consists of a congenital anomaly or birth defect

Other ‘important medical events’ may also be considered serious if they jeopardise the participant or require an intervention to prevent one of the above consequences.

## Definitions

| Adverse Event (AE) | Any untoward medical occurrence in a participant to whom a medicinal product has been administered, including occurrences which are not necessarily caused by or related to that product. |
| --- | --- |
| Adverse Reaction (AR) | An untoward and unintended response in a participant to an investigational medicinal product which is related to any dose administered to that participant.  The phrase "response to an investigational medicinal product" means that a causal relationship between a trial medication and an AE is at least a reasonable possibility, i.e. the relationship cannot be ruled out i.e. the relationship is definitely, probably, possibly or unlikely to be related (see below).  All cases judged by either the reporting medically qualified professional or the Sponsor as having a reasonable suspected causal relationship to the trial medication qualify as adverse reactions. |
| Serious Adverse Event (SAE) | A serious adverse event is any untoward medical occurrence that meets below definition and per Section 9.1:   - results in death - is life-threatening^4^ - requires inpatient hospitalisation^5^ or prolongation of existing hospitalisation - results in persistent or significant disability/incapacity - consists of a congenital anomaly or birth defect - Other ‘important medical event’. |
| Serious Adverse Reaction (SAR) | This is an adverse event that is both serious and is considered a drug reaction. |
| Suspected Unexpected Serious Adverse Reaction (SUSAR) | A SUSAR is a SAR that is:   - not listed in the summary of product characteristics (SmPC) for that product or - has not been described in the published literature before |
| Expectedness | An expected AR or SAR is a drug reaction that is listed in the SmPC and or has been described in the published literature before. |

NB: to avoid confusion or misunderstanding of the difference between the terms “serious” and “severe”, the following note of clarification is provided: “Severe” is often used to describe intensity of a specific event, which may be of relatively minor medical significance. “Seriousness” is the regulatory definition supplied above.

## Causality

The relationship of each adverse event to the trial medication must be determined by a medically qualified individual according to the following definitions:

Definitely related: There is clear evidence to suggest a causal relationship and other possible contributing factors can be ruled out.

Probably related: There is evidence to suggest a causal relationship and the influence of other factors is unlikely.

Possibly related: There is some evidence to suggest a causal relationship (e.g. because the event occurs within a reasonable time after administration of the trial medication). However, the influence of other factors may have contributed to the event (e.g. the patient’s clinical condition, other concomitant treatments).

Unlikely to be related: There is little evidence to suggest there is a causal relationship (e.g. the event did not occur within a reasonable time after administration of the trial medication), or there is another reasonable explanation for the event (e.g. the patient’s clinical condition, other concomitant treatment).

Not related: There is no evidence of any causal relationship.

## Procedures for Recording Adverse Events

The severity of adverse events will be assessed following the Common Terminology Criteria for Adverse Events (CTCAE) v5.0:

1 = mild, 2 = moderate, 3 = severe, 4 = life-threatening, 5 = fatal.

AEs occurring in participants from enrolment and during trial participation (up until Day 150 for a sub-set of participants with extended follow up) that are observed by the Investigator or reported by the participant with severity grade of 2 (moderate) or higher will be recorded on the CRF, whether or not attributed to trial medication.

The following information will be recorded: description, date of onset and end date, severity, assessment of relatedness to trial medication, other suspect drug or device and action taken. Follow-up information should be provided as necessary.

AEs considered related to the trial medication as judged by a medically qualified investigator will be followed either until resolution, or the event is considered stable.

It will be left to the Investigator’s clinical judgment to decide whether or not an AE is of sufficient severity to require the participant’s removal from treatment. A participant may also voluntarily withdraw from treatment due to what he or she perceives as an intolerable adverse event. If either of these occurs, the participant must undergo an end of trial assessment and be given appropriate care under medical supervision until symptoms cease, or the condition becomes stable.

## Reporting Procedures for Serious Adverse Events

General reporting procedures for all SAEs are to be managed by the site PI via local / national ethics committee and regulatory requirements.

In addition, the COPCOV safety team will monitor events and communicate with the study Data Safety and Monitoring Board (DSMB, see Section 9.7). The safety team can be contacted via [COPCOV-Safety@tropmedres.ac](mailto:COPCOV-Safety@tropmedres.ac)

SAEs relating to acquisition of COVID-19, and morbidity and mortality associated with this, do not need to be reported to the COPCOV safety team immediately, but should be reported no less than monthly in order to be included in scheduled Safety Monitoring Committee meetings.

All other SAEs detected by the site investigator should be reported to the COPCOV safety team within 24 hours of site awareness. The safety team and the local PI will gather any additional relevant information. The COPCOV safety team will inform the DSMB within 10 days of initial notification of the SAE and keep the DSMB updated as needed.

Treatment codes will be unblinded for specific participants after discussion with the study co-PI.

## Reporting Procedures for Pregnancy

If a female participant becomes pregnant after enrolment she should be instructed to discontinue study drug. The site study team is to notify the COPCOV safety team within 24 hours of site awareness through completion of the pregnancy notification form and submission to: [COPCOV-Safety@tropmedres.ac](mailto:COPCOV-Safety@tropmedres.ac)

Pregnant participants will be asked to return unused study medication at their next visit and will continue remaining follow up visits and procedures per protocol.

## Data Safety and Monitoring Board

An independent Data Safety and Monitoring Board (DSMB) will be set up consisting of qualified volunteers with the necessary knowledge of clinical trials. The DSMB will receive summary reports, prior to each meeting.

The DSMB will consider a formal interim analysis/analyses if the study exceeds certain time periods. The safety and the statistical considerations in the interim analyses such as the stopping rules for trial efficacy including the type 1 error probability, and futility for no treatment benefit have been clearly detailed in the DSMB charter and the statistical analysis plan.

All data reviewed by the DSMB will be in the strictest confidence. A DSMB charter will outline its responsibilities, number of interim reports and how it will operate. Interim reports will be prepared by the Trial Statistician.

All DSMB recommendations will be communicated to site PIs. The site PI will be responsible for submitting the written DSMB summary reports with recommendations as applicable to local/ national ethics committees and other applicable groups.

# STATISTICS AND ANALYSIS

## Description of Statistical Methods

All participant data will be included in the Intention-To-Treat (ITT) analysis according to the arm they were randomised to, irrespective of the actual study drug that they took. This ITT analysis will be the main strategy for the primary outcome and will be followed by a per protocol (PP) analysis. A per protocol (PP) analysis will be conducted to adjust for non-compliance to study protocols. Under an assumption of no post-randomisation confounding, this is a form of sensitivity analysis of the intention to treat analysis. In the PP analysis, participants who did not take their pills, or those who took extra chloroquine/ hydroxychloroquine (in both cases as determined by PK analyses), no final outcome assessed and losses to follow-up prior to the assessment of the final outcome, and any major protocol violations will be excluded. A detailed Analysis plan will be written by the trial statistician.

## The Number of Participants

A large and definitive study is needed to characterise the benefit of prophylaxis with chloroquine or hydroxychloroquine in protecting heath care workers (and other persons defined at risk of contracting COVID-19) from COVID-19 illness. Power calculations are based on an assumption of 3% incidence of symptomatic COVID-19 during the trial period (1% per month). This is a conservative estimate and although some sites may have more or less cases, due to the unpredictability of numbers of cases in a site and a country, this assumption remains valid. Expert opinion considers that if chloroquine or hydroxychloroquine is effective, it may decrease symptomatic COVID-19 by 23%, and therefore, the chloroquine arm or hydroxychloroquine would have a 2.31% COVID-19 diagnosis. A 95% confidence interval with 80% power would indicate 8,520 participants randomised to each arm. We will aim to enrol 10,000 participants in each arm in the two trials which allows for at least a 10% LTFU, withdrawal rate, protocol deviation and non-adherence. Thus 20,000 would be randomised to chloroquine/ placebo and 20,000 to hydroxychloroquine/ placebo.

## Analysis of Outcome Measures

A mixed effects Negative Binomial model will be used to model the incidence of symptomatic COVID-19 infection to obtain incidence rate ratios comparing the chloroquine arm with the placebo. Repeated measures and hospital clustering effect will be taken to account in the mixed effects model. Incidence rate ratios and the corresponding 95% confidence intervals will be obtained and reported. As much as possible graphical methods will be used to show trends in the incidence of symptomatic COVID-19 over time and by arm. In the event that the Negative Binomial models fail to converge, as is the characteristic of these models when the outcome is rare, a Binomial regression model will be considered to model the risk/ odds of symptomatic COVID-19 infection to obtain risk differences/ odds ratios as appropriate comparing the chloroquine/ hydroxychloroquine arm with placebo. Survival methods will be used to estimate the time to resolution and also as a method of handling missing data in case of dropouts. In this approach, participants without outcomes will be censored at their longest observed time.

A continuous severity score (see Appendix A for example) will be used to assess severity of symptomatic COVID-19 and ARIs of those who acquire these, and these will be captured longitudinally over time. A rank-based mixed model approach will be used to analyse these scores, to compare the two groups in order to discriminate between severities at the lower end of the spectrum.

Normally distributed continuous baseline characteristics will be summarised using means and standard deviations while skewed continuous baseline characteristics will be summarised medians and interquartile ranges. Categorical data will be summarised using counts and percentages. A Fisher’s exact test will be used to compare binary outcome data between groups. Statistical significance will be determined at 5% significance level.

# DATA MANAGEMENT

## Access to Data

Direct access will be granted to authorised representatives from the University of Oxford, local ethics committees and regulatory authorities, and any host institution for monitoring and/or audit of the study to ensure compliance with regulations.

## Data Handling and Record Keeping

Clinical study data will be recorded on CRFs and entered on to a password-protected database by the local study PI, a research nurse or designee. The study database will be built in a clinical data management system that is compliant with ICH GCP and FDA 21 CFR Part 11 and will be hosted in a secure, access-restricted server. A system for recording electronic patient reported outcomes (ePRO) will be built and integrated with the study database. The study database and ePRO system will include internal quality checks to identify data that appear inconsistent, incomplete, or inaccurate.

Measures will be taken to ensure non-disclosure of information that is potentially harmful to participants. Paper records (for example, patient identifiable information for the purposes of follow-up, the screening logs and signed ICFs) will be kept in locked cabinets; electronic data will only be accessible to staff with user accounts and passwords. The database contains an audit trail that keeps record of changes to data and user activity within the database. All electronic data will be stored on secure servers that are backed up daily, with weekly off-site storage.

Participant records at site will, taking into account the ability of the sites, be stored in binders in the secured access-limited room or scanned and stored electronically. The records will be retained for five years following completion of the study, or according to local site regulation. The study database will be retained indefinitely.

With participant’s consent, clinical data and results from blood analyses stored in the database may be shared according to the terms defined in the MORU data sharing policy with other researchers to use in the future.

Data generated from this study will adhere to the 2016 “[Statement on data sharing in public health emergencies](https://wellcome.ac.uk/what-we-do/our-work/statement-data-sharing-public-health-emergencies)”(<https://wellcome.ac.uk/press-release/statement-data-sharing-public-health-emergencies>).

# QUALITY CONTROL AND QUALITY ASSURANCE PROCEDURES

The study will be conducted in accordance with relevant regulations and standard operating procedures.

The study will be conducted in compliance with this protocol, International Conference on Harmonization (ICH) Guidelines for Good Clinical Practice (GCP) and any applicable regulatory requirement(s). Monitoring will be overseen by the MORU Clinical Trials Support Group (CTSG) according to a prespecified risk-based monitoring plan to ensure compliance to the study protocol and applicable guidelines and regulations. Blood samples will be processed, stored and shipped in accordance with MORU SOPs.

Data validation will be performed to identify errors or discrepancies and thus ensure completeness, validity and accuracy of data.

# ETHICAL AND REGULATORY CONSIDERATIONS

## Declaration of Helsinki

The Investigator will ensure that this study is conducted in accordance with the principles of the Declaration of Helsinki.

## Guidelines for Good Clinical Practice

The Investigator will ensure that this study is conducted in accordance with relevant regulations and with Good Clinical Practice.

## Approvals

The protocol, informed consent form, participant information sheet and any proposed advertising material (if applicable) will be submitted to OxTREC and applicable local/ national ethics committees and regulatory agencies for written approval.

The Investigator will submit and, where necessary, obtain approval from the above parties for all amendments to the original approved documents.

## Participant Confidentiality

The study team will ensure that the participants’ anonymity is maintained. The participants will be identified only by a participant ID number on all study documents and any electronic database, with the exception of the CRF, where participant initials may be added. All documents will be stored securely and only accessible by study team and authorised personnel. The study will comply with the Data Protection Act 2018, which requires that personal data must not be kept as identifiable data for longer than necessary for the purposes concerned.

## Expenses and Benefits

Participants will not be paid for their participation in the research. Reimbursement for costs incurred by participants during study participation will be reimbursed per local allowed guidelines and ethics committee policies.

## Reporting

The PI will ensure that an Annual Progress Report is submitted to all applicable ethics committees and regulatory agencies on the anniversary of the date of approval of the study, or as otherwise defined per local requirement. In addition, the PI shall submit an End of Study Report to all applicable parties upon completion of the study.

## Other Ethical Considerations

The decision to include only participants with a smartphone potentially runs the risk of violating the “fair subject principal” (28) i.e. introducing a socio-economic bias into the trial. Given the trial will be conducted in employed workers in facilities providing care for COVID-19, we do not think there will be many who would not be able to be enrolled on this basis. Additionally, as we have already selected a discrete group on whom to conduct the study we do not think excluding those without a smartphone will add any additional meaningful bias which will affect the study objectives.

Given the urgency of the question which this trial aims to answer and the difficulty of collecting the same information without the use of an app-enabled smartphone we believe that the prompt and definitive answering of the trial question is in society’s best interests, and given the current equipoise between chloroquine / hydroxychloroquine and placebo in prevention of COVID-19, does not disadvantage those ineligible to enrol.

## Community and public engagement

Given the current lack of evidence that chloroquine or hydroxychloroquine will be effective in the prevention of COVID-19, there is currently scientific equipoise which justifies the use of placebo in this study. Although chloroquine or hydroxychloroquine have both been shown to be very safe, the medication is not without side-effects.

As part of our engagement initiative (also called “patient and public involvement”, MORU will be conducting a series of workshops within Thailand with (1) potential participants e.g. hospital staff and (2) members of the public via existing advisory groups such the Bangkok Health Research Interest Group and community advisory boards, to embed their voices into the research design, implementation and dissemination of findings) (see Appendix C).

Similar or additional activities may be implemented at sites outside Thailand.

# FINANCE AND INSURANCE

## Funding

This study is funded via the ACT-Accelerator Therapeutics Partnership.

## Insurance

The University has a specialist insurance policy in place which would operate in the event of any participant suffering harm as a result of their involvement in the research (Newline Underwriting Management Ltd, at Lloyd’s of London).

# PUBLICATION POLICY

All publications will abide by the International Committee of Medical Journal Editors (ICMJE) recommendations of the role of authors and contributors.

The results of the study will be summarised in lay language, in both English and the language(s) commonly spoken at the study sites, and disseminated to key stakeholders, user communities and caretakers of study participants.

# REFERENCES

1. Chen Y, Chen L, Deng Q, Zhang G, Wu K, Ni L, et al. The presence of SARS-CoV-2 RNA in the feces of COVID-19 patients. J Med Virol. 2020;92(7):833-40.

2. Group RC, Horby P, Lim WS, Emberson JR, Mafham M, Bell JL, et al. Dexamethasone in Hospitalized Patients with Covid-19 - Preliminary Report. N Engl J Med. 2020.

3. Lambert LC, Fauci AS**.** Influenza vaccines for the future. N Engl J Med. 2010;363(21):2036-44.

4. Zhao S, Lin Q, Ran J, Musa SS, Yang G, Wang W, et al. Preliminary estimation of the basic reproduction number of novel coronavirus (2019-nCoV) in China, from 2019 to 2020: A data-driven analysis in the early phase of the outbreak. Int J Infect Dis. 2020.

5. Biggerstaff M, Cauchemez S, Reed C, Gambhir M, Finelli L**.** Estimates of the reproduction number for seasonal, pandemic, and zoonotic influenza: a systematic review of the literature. BMC Infect Dis. 2014;14:480.

6. Wang Z, Chen X, Lu Y, Chen F, Zhang W**.** Clinical characteristics and therapeutic procedure for four cases with 2019 novel coronavirus pneumonia receiving combined Chinese and Western medicine treatment. Biosci Trends. 2020.

7. White NJ, Miller KD, Churchill FC, Berry C, Brown J, Williams SB, et al. Chloroquine treatment of severe malaria in children. Pharmacokinetics, toxicity, and new dosage recommendations. N Engl J Med. 1988;319(23):1493-500.

8. White NJ, Watt G, Bergqvist Y, Njelesani EK**.** Parenteral chloroquine for treating falciparum malaria. J Infect Dis. 1987;155(2):192-201.

9. Krishna S, White NJ**.** Pharmacokinetics of quinine, chloroquine and amodiaquine. Clinical implications. Clin Pharmacokinet. 1996;30(4):263-99.

10. Villegas L, McGready R, Htway M, Paw MK, Pimanpanarak M, Arunjerdja R, et al. Chloroquine prophylaxis against vivax malaria in pregnancy: a randomized, double-blind, placebo-controlled trial. Trop Med Int Health. 2007;12(2):209-18.

11. Payne D**.** Did medicated salt hasten the spread of chloroquine resistance in Plasmodium falciparum? Parasitol Today. 1988;4(4):112-5.

12. Wang M, Cao R, Zhang L, Yang X, Liu J, Xu M, et al. Remdesivir and chloroquine effectively inhibit the recently emerged novel coronavirus (2019-nCoV) in vitro. Cell Res. 2020.

13. Vincent MJ, Bergeron E, Benjannet S, Erickson BR, Rollin PE, Ksiazek TG, et al. Chloroquine is a potent inhibitor of SARS coronavirus infection and spread. Virol J. 2005;2:69.

14. Lu R, Zhao X, Li J, Niu P, Yang B, Wu H, et al. Genomic characterisation and epidemiology of 2019 novel coronavirus: implications for virus origins and receptor binding. Lancet. 2020.

15. Zhou P, Yang XL, Wang XG, Hu B, Zhang L, Zhang W, et al. A pneumonia outbreak associated with a new coronavirus of probable bat origin. Nature. 2020.

16. Yao X, Ye F, Zhang M, Cui C, Huang B, Niu P, et al. In Vitro Antiviral Activity and Projection of Optimized Dosing Design of Hydroxychloroquine for the Treatment of Severe Acute Respiratory Syndrome Coronavirus 2 (SARS-CoV-2). Clin Infect Dis. 2020.

17. McChesney EW, Banks WF, Jr., Fabian RJ**.** Tissue distribution of chloroquine, hydroxychloroquine, and desethylchloroquine in the rat. Toxicol Appl Pharmacol. 1967;10(3):501-13.

18. Keyaerts E, Vijgen L, Maes P, Neyts J, Van Ranst M**.** In vitro inhibition of severe acute respiratory syndrome coronavirus by chloroquine. Biochem Biophys Res Commun. 2004;323(1):264-8.

19. Keyaerts E, Li S, Vijgen L, Rysman E, Verbeeck J, Van Ranst M, et al. Antiviral activity of chloroquine against human coronavirus OC43 infection in newborn mice. Antimicrob Agents Chemother. 2009;53(8):3416-21.

20. He X, Lau EHY, Wu P, Deng X, Wang J, Hao X, et al. Temporal dynamics in viral shedding and transmissibility of COVID-19. Nat Med. 2020;26(5):672-5.

21. Group RC, Horby P, Mafham M, Linsell L, Bell JL, Staplin N, et al. Effect of Hydroxychloroquine in Hospitalized Patients with Covid-19. N Engl J Med. 2020.

22. Hongchao Pan RP, Quarraisha Abdool Karim, Marissa Alejandria, Ana Maria Henao-Restrepo, César Hernández García, Marie-Paule Kieny, Reza Malekzadeh, Srinivas Murthy, Marie-Pierre Preziosi, Srinath Reddy, Mirta Roses Periago, Vasee Sathiyamoorthy, John-Arne Røttingen, Soumya Swaminathan**.** Repurposed antiviral drugs for COVID-19 –interim WHO SOLIDARITY trial results. medRxiv. 2020.

23. Xabier García-Albéniz JdA, Rosa Polo, José Miguel Morales-Asencio, Miguel A Hernán**.** Brief communication: A meta-analysis of randomized trials of hydroxychloroquine for the prevention of COVID-19. medRxiv 2020.09.29.20203869; doi: <https://doi.org/10.1101/2020.09.29.20203869>. 2020.

24. Lofgren SMM, Nicol MR, Bangdiwala AS, Pastick KA, Okafor EC, Skipper CP, et al. Safety of Hydroxychloroquine among Outpatient Clinical Trial Participants for COVID-19. medRxiv. 2020.

25. Lane JCE, Weaver J, Kostka K, Duarte-Salles T, Abrahao MTF, Alghoul H, et al. Risk of hydroxychloroquine alone and in combination with azithromycin in the treatment of rheumatoid arthritis: a multinational, retrospective study. Lancet Rheumatol. 2020.

26. White NJ, Watson JA, Hoglund RM, Chan XHS, Cheah PY, Tarning J**.** COVID-19 prevention and treatment: A critical analysis of chloroquine and hydroxychloroquine clinical pharmacology. PLoS Med. 2020;17(9):e1003252.

27. Watson JA, Tarning J, Hoglund RM, Baud FJ, Megarbane B, Clemessy JL, et al. Concentration-dependent mortality of chloroquine in overdose. Elife. 2020;9.

28. Emanuel EJ, Wendler D, Killen J, Grady C**.** What makes clinical research in developing countries ethical? The benchmarks of ethical research. J Infect Dis. 2004;189(5):930-7.

# APPENDIX A: EXAMPLE OF SEVERITY OUTCOME MEASURES

| **Observation** | **Scale** |
| --- | --- |
| **Outpatient** |  |
| Feels normal |  |
| Feels unwell |  |
| URTI Symptoms (coryza and/ or sore throat) |  |
| Muscle aches |  |
| Cough |  |
| Afebrile <37.5^o^C |  |
| Fever ≥37.5 and ≤38.5^o^C |  |
| High fever >38.6^o^C |  |
| Shortness of breath on exertion |  |
| Shortness of breath at rest |  |
| Mainly chair/ bed bound |  |
| Requires hospitalisation (based on clinical symptoms) |  |

| **Inpatient: Hospitalisation** |  |
| --- | --- |
| Hypoxia / Hypoxaemia 90-95% on air (if measured) or requiring supplemental O_2_ (not high-flow) |  |
| Hypoxia < 90% on air (if measured) or on supplemental O_2_ (up to 15L on non-rebreather) |  |
| Tachypnoea RR 25-40 |  |
| Tachypnoea RR ≥ 40 |  |
| ARDS |  |
| Non-invasive ventilation: high-flow or CPAP/ BiPAP |  |
| Mechanical ventilation |  |
| Organ support other than respiratory |  |
| ECMO criteria met |  |
| Death |  |

The above observations form an example of what may be collected and form the severity outcome scale for participants in the trial who have symptomatic COVID-19 and also for those with an ARI. Additionally, data will be collected on the duration of symptoms and analysed to determine if a difference in severity exists between the two arms. As part of our analysis plan we will explore grouping, weighting and aggregation of the above observations. A more detailed analysis plan can be found in the statistical analysis plan (SAP) document.

# APPENDIX B: SCHEDULE OF STUDY PROCEDURES

| **Procedures** | **Visits** | | | | | | | |
| --- | --- | --- | --- | --- | --- | --- | --- | --- |
|  | **Day 0**  **Enrolment** | **Day 30**  **(-3 / +1)** | **Day 60**  **(-3 / +1)** | **Day 90**  **(-3 / +7)** | **ARI symptom onset^1^** | **Outcome follow-up if symptomatic (≤Day 150)** | **Prior to vaccine**  **(or within 3 days after first dose)** | **Day 28**  **(+/-3 days) post first vaccine dose** |
|  | **1** | **2** | **3** | **4** | ***As needed during trial period*** | **5** | ***Vaccinated participants ONLY*** | |
| Screening | X |  |  |  |  |  |  |  |
| Eligibility assessment | X |  |  |  |  |  |  |  |
| Informed consent | X |  |  |  |  |  |  |  |
| Demographics | X |  |  |  |  |  |  |  |
| Medical history | X |  |  |  |  |  |  |  |
| Randomisation | X |  |  |  |  |  |  |  |
| Set up mobile app | X |  |  |  |  |  |  |  |
| Given thermometer | X |  |  |  |  |  |  |  |
| Venous blood test | Y |  |  | Z |  | Z^2^ | Z^3^ | Z^3^ |
| Observed 1^st^ dose of study medication | X |  |  |  |  |  |  |  |
| Dispensation of study medication (unless diagnosed as COVID-19 before visit) | X | X | X |  |  |  |  |  |
| Compliance assessment |  | X | X | X |  |  | X |  |
| DBS | X* | X | X | X* |  |  | X* |  |
| Adverse event assessments |  | X | X | X |  |  | X | X |
| Questions about well-being, illness, COVID-19 diagnosis and clinical severity data | X | X | X | X | X | X | X | X |
| Nose and throat swab (+/- sputum) |  |  |  |  | X |  |  |  |

^1^ Can be repeated on multiple occasions if illness worsens or new ARI during trial period.

^2^ If not already collected at Day 90.

^3^ If these timepoints are collected in vaccinated participants then no sample will be collected at Day 90.

Y 10mls of venous blood Z 5mls of venous blood

* This sample is expected to be obtained from the venous blood sample drawn at the same visit. If necessary direct finger prick may be performed.

# APPENDIX C: EXAMPLE OF COMMUNITY AND PARTICIPANT ENGAGEMENT

We will conduct a series of “Patient & Public Involvement” workshops, interviews and group discussions (virtual or face-to-face) with two different groups of stakeholders (i) potential participants e.g. hospital staff and (ii) members of the public via e.g. existing advisory groups such the Bangkok Health Research Interest Group and community advisory boards.

These activities will be conducted at various stages with the following objectives:

(1) protocol development stage and prior to study start:

- to inform study design, procedures, participant information materials, develop frequently asked questions (FAQs)
- to seek general attitudes about the study, use of placebo and study procedures
- to develop communication materials for dissemination to the wider public and media about the study, how best to communicate the meaning and rationale of “placebo”, “blinding”, etc
- to identify what could motivate and/or discourage those to meet eligibility criteria to join the study
- to determine to what extent is chloroquine is available from local pharmacies and/or informal vendors

(2) soon after study start:

- to identify any challenges in recruitment, study procedures, understanding of the study, and potential solutions

(3) after study completion:

- to disseminate the study results
- to inform strategies for dissemination of study results to the wider public so that they can use and find value in the research

In all stages, we will also be seeking the general attitudes about the epidemic e.g. fears, perception of risks, measures taken by individuals to protect themselves, likelihood of chloroquine or other drug self-medication, perception of public health measures (e.g. social distancing), and economic consequences. Understanding these social factors will help us with communication with participants, their families and the general public.

# APPENDIX D: POTENTIAL SITES

**The Faculty of Tropical Medicine (FTM), Mahidol University, Bangkok**, is affiliated and partnered with the Mahidol Oxford Tropical Medicine Research Unit (MORU), which have successfully collaborated together for more than 40 years in the management of Tropical diseases. FTM is the only faculty specialising in tropical medicine in Thailand and runs the Hospital for Tropical Diseases in Bangkok. Both FTM and MORU are fitted with state-of-the-art molecular diagnostic testing facilities.

**Sunpasithiprasong Hospital, Ubon Ratchathani**. Situated in the East of Thailand on the Laos border, this long-term 1200 bed hospital and MORU collaborator for 34 years has dedicated on-site study nurses currently conducting clinical trials.

**Udon Thani Hospital, Udon Thani**, (800 beds) is situated in the Northeast of Thailand adjacent to Lao’s capital, Vientiane, and has been a study site for previous trials with successful collaboration.

**Chiangrai Clinical Research Unit (CCRU)** is a MORU-affiliated unit in the North of Thailand. It has a full-time team of a clinician researcher, study nurses and laboratory staff, with a laboratory based in the Prachanukroh Hospital (760 beds).

**Shoklo Malaria Research Unit (SMRU), Mae Sot**, based on the Thai-Myanmar border in the West of Thailand is the largest of the MORU-affiliated sites. Originally set up to provide basic healthcare and malaria treatment to Myanmar migrants fleeing civil war in Kayin (Karen) state, this unit now supports and runs several in-patient clinics, has a fully accredited laboratory able to test for COVID-19 and other respiratory pathogens and works closely with the Thai Governmental hospital in the city.

**Lao-Oxford-Mahosot Hospital-Wellcome Trust Research Unit (LOMWRU)**, a clinical research unit embedded in the 450 bed Mahosot Hospital in Lao PDR’s capital, Vientiane, conducts clinical research on diseases of regional public health importance, and has recently developed capability for testing for SARS-CoV-2, as well as other respiratory viruses and causes of febrile illness. Audrey Dubot-Pérès, the head of Virology at LOMWRU and also works at the Institut de Recherche pour le Développement (IRD) in the Unit of Emerging Viruses (Aix-Marseille Univ-IRD 190-Inserm 1207-IHU Méditerranée Infection), Marseille. Additionally LOMWRU has excellent links, and is currently collaborating in, 5 provincial hospitals, which could be potential study sites: Xieng Khuang; Luang Namtha; Salavan; Savannakhet; and Phonhong. These provinces provide health care to the provinces which have porous borders with China, Myanmar and several geographically distinct regions of Thailand and Vietnam.

**Myanmar-Oxford Clinical Research Unit (MOCRU)** is a MORU-affiliated unit in Yangon, Myanmar with strong links to Medical Action Myanmar (MAM) a non-governmental organisation which runs a network of 2,000 health workers and 10 clinics as well as strong collaborative links with the Myanmar Ministry of Health and several public hospitals in Yangon and Mandalay.

**Cambodia-Oxford Medical Research Unit (COMRU)** a collaboration between MORU and the Angkor Hospital for Children (AHC) in Siem Reap, Cambodia. This collaboration started in 2006 and led by Professor Paul Turner, head of COMRU, and Dr Claudia Turner, CEO of AHC, provides free, quality healthcare to approximately 450 children a day, as well as high-quality research. The laboratory is equipped to do respiratory virus testing, including COVID-19 as well as whole genome sequencing of isolates. Given the volume of children presenting with respiratory illnesses, COVID-19 could quite easily spread to healthcare providers and back out to the community.

**Hospital of Tropical Diseases (HTD), Ho Chi Minh City, Vietnam** The Oxford University Clinical Research Unit (OUCRU), our sister unit based in the HTD, has a long history of conducting hospital-based clinical trials and was at the forefront of the research response to Avian Influenza in 2004 led by Professor Jeremy Farrar. HTD is a regional referral hospital serving a population of 38 million in the South of the country.

**The Christian Medical College (CMC), Vellore, India**. An institution currently collaborating with MORU, Bangkok on the biggest scrub typhus trial ever conducted and the only trial on severe scrub typhus. CMC comprises 3,000 hospital beds across 6 campuses providing primary to quaternary care management of patients. CMC consistently ranks as one of the top medical institutions in India.

**Other potential sites will be located in, but are not limited to, the following countries:**

| **Africa** | Benin, Botswana, Burkina Faso, Cameroon, Côte d’Ivoire, Democratic Republic of Congo, Egypt, Ethiopia, Gambia, Ghana, Guinea, Kenya, Mali, Mozambique, Niger, Nigeria, Senegal, Sierra Leone, South Africa, Sudan, Tanzania, Tunisia, Uganda, Zambia, Zimbabwe |
| --- | --- |
| **Asia** | Bangladesh, India, Indonesia, Malaysia, Maldives, Nepal, Pakistan, Saudi Arabia, Vietnam |
| **Europe** | Croatia, France, Italy, Netherlands, Russian Federation, Switzerland, Ukraine, United Kingdom |
| **North / South America** | Argentina, Brazil, Canada, Chile, Colombia, Cuba, Dominican Republic, Guatemala, Jamaica, Peru, Puerto Rico |

# APPENDIX E: AMENDMENT HISTORY

| **Amendment No.** | **Protocol Version No.** | **Date issued** | **Author(s) of changes** | **Details of Major Changes** |
| --- | --- | --- | --- | --- |
| 1 | 2.0 | 23 Mar 2020 | Dr Will Schilling, Dr Nick White | Study population increased to 40,000 |
|  |  |  |  | Hydroxychloroquine added as study drug for sites in Europe |
| 2 | 3.0 | 06 Apr 2020 | Dr Will Schilling, Dr Nick White | Co-primary objective related to symptom severity now secondary |
|  |  |  |  | Study population clarified as healthcare workers (other “high-risk” population removed) |
|  |  |  |  | To ensure that the primary outcome measures are clear, a table has been added: *Overview of primary endpoint ascertainment* |
|  |  |  |  | Procedures for follow up of participants if unwell clarified (multiple sections) |
|  |  |  |  | Provision added for sites to enrol up to 200 participants if agreed with MORU |
|  |  |  |  | Safety reporting procedures updated to acknowledge local reporting requirements |
|  |  |  |  | AE capture clarified to Grade 2 or above (per CTCAE scale) |
|  |  |  |  | The statistical analysis was clarified that 1) randomisation will be stratified by site, 2) a mixed effects negative binomial regression model will be used for the primary outcome, and 3) a rank-based mixed model approach will be used to analyse these scores |
|  |  |  |  | Funding source clarified to Wellcome Trust via COVID-19 Therapeutics Accelerator |
|  |  |  |  | Africa added as study location |
| 3 | 4.0 | 06 Apr 2020 | Dr Will Schilling, Dr Nick White | Tertiary outcome and procedures added for EQ-5D-3L assessment, to assess the potential impact of study drug prophylaxis on work or behaviour during the COVID-19 pandemic |
|  |  |  |  | Study population further clarified as healthcare workers with direct patient contact |
| 4 | 5.0 | 30 Jun 2020 | Dr Will Schilling, Dr Nick White | Participant inclusion has been broadened to include individuals working in healthcare facilities who are not providing direct patient care |
|  |  |  |  | Additional exclusion criteria and prohibited medications added after regulatory discussions re pregnancy and potential cardiotoxicity. Upper age for participation clarified as <70 years old |
|  |  |  |  | Clarification added in study design for participants to stop study medication if they are hospitalized due to COVID-19 illness |
|  |  |  |  | Addition of possible interim analysis to be completed by DSMB |
|  |  |  |  | Pregnancy reporting process added |
|  |  |  |  | Visit window expanded to 27-31 days |
| 5 | 6.0 | 13 Jan 2021 | Dr Will Schilling, Dr Nick White | Defined procedures for management of participants who may receive COVID-19 vaccination after enrolment |
|  |  |  |  | Clarified potential exploratory objectives which may be included in analysis, e.g., pharmacokinetic analysis of study medication and evaluation of antibody response in vaccinated participants |
|  |  |  |  | Updated benefit and risk profile for study medications and design |
|  |  |  |  | Participant inclusion has been further broadened to include individuals otherwise defined by site investigator as at risk for COVID-19 infection |
|  |  |  |  | Exclusion criteria have been updated to clarify that participants may not be enrolled with prior COVID-19 vaccination or current use of chloroquine or hydroxychloroquine |
|  |  |  |  | Study procedures updated to allow once daily reporting of participant temperature and symptoms |
|  |  |  |  | Further defined categories meeting serious adverse events (SAEs) |
|  |  |  |  | Clarified management of participants who discontinue study medication early for follow up through D90 |

Note: The following additional changes have been implemented in each revision as necessary, and are not individually itemized:

1. Updated background regarding COVID-19.
2. Site list for potential participating countries.
3. Minor formatting and consistency corrections.

1. World Health Organization’s COVID-19 situation report [↑](#footnote-ref-2)
2. World Health Organization’s Factsheet on Seasonal Influenza [↑](#footnote-ref-3)
3. World Health Organization’s list of Essential Medications [↑](#footnote-ref-4)
4. The term "life-threatening" in the definition of "serious" refers to an event in which the participant was at risk of death at the time of the event; it does not refer to an event which hypothetically might have caused death if it were more severe. [↑](#footnote-ref-5)
5. Hospitalisation is defined as an unplanned, formal inpatient admission, even if the hospitalisation is a precautionary measure for continued observation. The participant must be admitted overnight, a short stay of several hours to receive treatment is not considered hospitalisation. If a patient is admitted overnight or longer for social/economic reasons and is otherwise medically stable, this does not constitute a SAE. Other examples of visits to a hospital facility that are not considered hospitalisation are: Emergency room visits, outpatient surgery and pre-planned or elective procedures for a pre-existing condition (as long as that condition has not deteriorated while on trial treatment or brought forward because of worsening symptoms). [↑](#footnote-ref-6)
