# Supplementary material for: Evaluation of hydroxychloroquine or chloroquine for the prevention of COVID-19 (COPCOV): A double-blind, randomised, placebo-controlled trial
Source: PLoS Med. 2024 Sep 12;21(9):e1004428. doi: 10.1371/journal.pmed.1004428 (PMC11392261; doi:10.1371/journal.pmed.1004428)
Supplement: S1 Statistical Analysis Plan — (PDF) [file pmed.1004428.s005.pdf]

STATISTICAL ANALYSIS PLAN

Chloroquine/ hydroxychloroquine prevention of coronavirus disease (COVID-19) in the healthcare setting; a randomised, placebo-controlled prophylaxis study (COPCOV)

Internal Reference Number / Short title: COPCOV

Version 1.0 FINAL **SAP**

Date 10th Dec 2022

Written by:

Professor Mavuto Mukaka (Statistician) Signature______________Date- 10 December, 2022

Dr James Watson (Statistician)

Dr Arjun Chandna (Investigator

Dr James Callery (Investigator)

Reviewed by and approved by:

Prof Sir Nicholas White (Co-PI) Signature__________________Date 10 December 2022

Dr William Schilling (Co-PI) Signature__________________Date- 10 Dec 2022

Dr Walter Taylor (Investigator)

Prof Nicholas Day (Investigator)

Prof Arjen Dondorp (Investigator)

Trial Overview 2
1.1 Main research questions 3
1.2 COPCOV trial: Overview of primary endpoint ascertainment (i.e. symptomatic COVID-19): 4
Statistical Analysis Considerations 10
1.3 General Analysis Approach 10
1.3.1 Missing data 10
1.4 Primary objective 10
1.4.1 Primary endpoint 11
1.4.2 Secondary objectives and endpoints 11
1.4.3 Tertiary objective and endpoint 11
Study design 11
1.5 Determination of sample size 12
Data Analysis 12
1.6 Trial Profile 12
1.7 Demographics and other baseline characteristics 13
1.8 Comparisons of incidence/risk rates of symptomatic COVID-19 infections between the chloroquine or hydroxychloroquine and placebo groups 15
1.9 Safety of Chloroquine/Hydroxychloroquine 19
1.10 Meta-Analysis 20
1.11 Pharmacokinetic analysis of Chloroquine/Hydroxychloroquine 20
1.12 Antibody response to vaccination for chloroquine/hydroxychloroquine 21
Secondary objectives and endpoints 21
1.13 Comparison of severity and duration of COVID between the chloroquine/ hydroxychloroquine and placebo using a severity score. 21
1.13.1 Severity score table 22
1.14 Determining if chloroquine or hydroxychloroquine prophylaxis prevents asymptomatic COVID-19 infection. 23
1.15 Determining if chloroquine/ hydroxychloroquine prophylaxis prevents all-cause symptomatic acute respiratory illnesses. 23
1.16 Tertiary objective and endpoint 25
References 26

Trial Overview

We hypothesise that chloroquine and hydroxychloroquine might slow viral replication in SARS CoV2 infected participants, attenuating or preventing the COVID-19 illness. Given the enormous experience of their use in malaria chemoprophylaxis and autoimmune diseases, excellent safety and tolerability profile, and their very low cost, then if these drugs proved effective, chloroquine and hydroxychloroquine would be a readily deployable and affordable preventive measure for high-risk individuals such as healthcare workers.

1.1 Main research questions

The primary objective of the COPCOV study is to determine if prophylactic chloroquine or hydroxychloroquine prevents symptomatic COVID-19 illness. This is defined as symptoms in keeping/compatible with COVID-19 AND laboratory evidence of infection defined as either virologically confirmed infection (a PCR positive for SARS-CoV-2), OR if the PCR fails or is not done, then serologically confirmed infection (see below Overview of primary endpoint ascertainment).

1.2 COPCOV trial: Overview of primary endpoint ascertainment (i.e. symptomatic COVID-19):

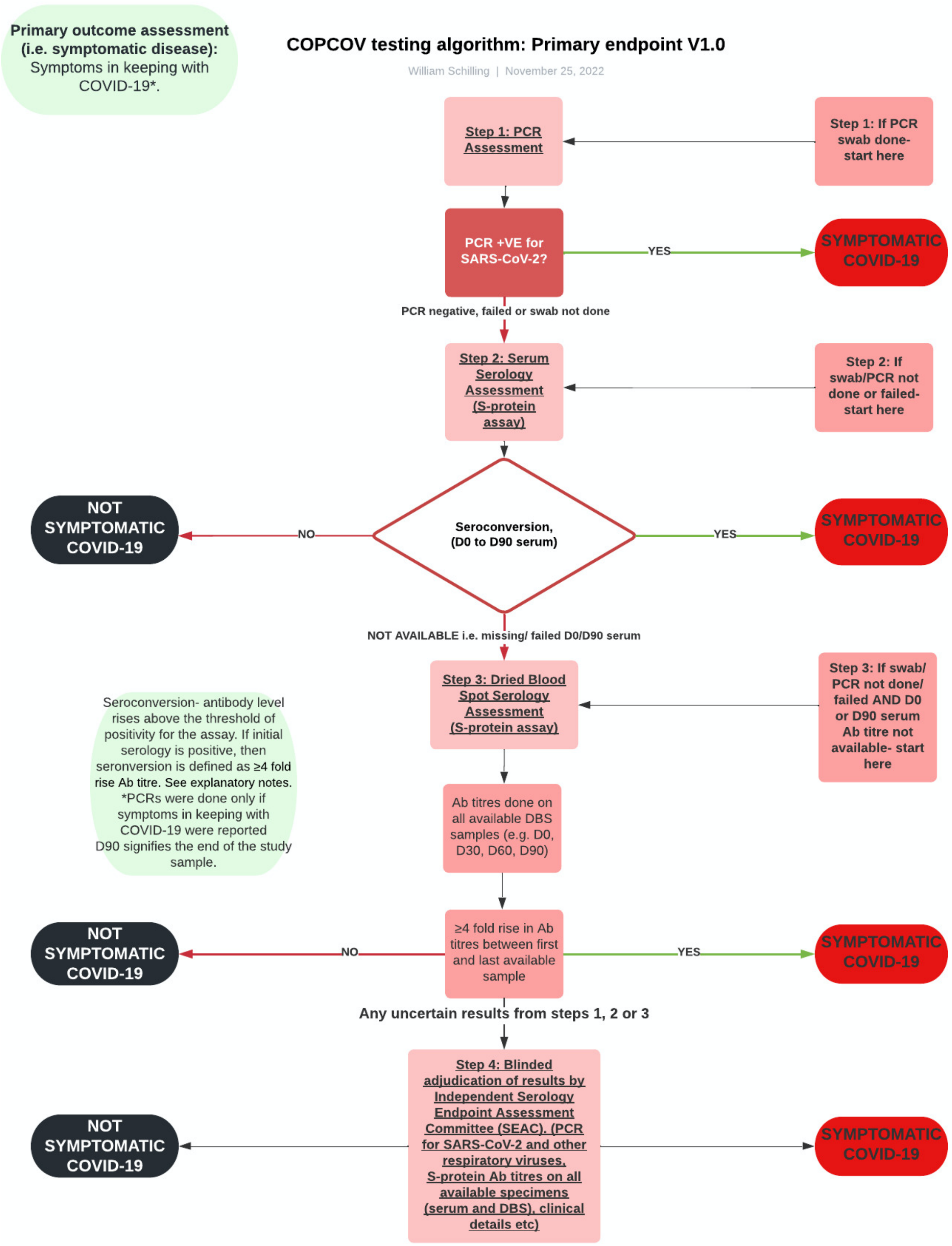

Figure 1- COPCOV testing algorithm for the primary endpoint prior to SEAC review (changes documented on page 8). Explanatory notes below.

D90 indicates end of study. For some participants, D90 samples were collected outside the window set in the protocol. For those vaccinated, the end of study sample (equivalent to the D90 sample for completed follow up) was collected around the

time of vaccination (no more than 3 days after vaccination). For the purposes of the diagnostic algorithm, these will be included and considered to be end of study (D90), so D90 signifies end of study samples, whenever they were taken.

Below we present the statistical analysis plan (SAP) and rationale as proposed by the investigators. We realised that there would be some cases where the trial outcome would not be clear cut. To adjudicate on these few cases an independent serology trial endpoints assessment committee (SEAC) was convened. The complete blinded serology and PCR datasets were presented to the SEAC. For cases referred to the SEAC, their final judgement was the outcome recorded in the database. We present the SAP as proposed originally, followed by the minor changes made by the SEAC and accepted by the investigators. These are the definitions and criteria upon which each participant's individual trial outcome is recorded in the trial database. The database will be locked and date-time stamped before unblinding.

A. *Definitions of symptomatic COVID-19 and asymptomatic COVID-19:*

COVID-19 infection encompasses the spectrum of asymptomatic, pauci-symptomatic (mild symptoms) to severe symptoms and signs which can result in hospitalisation and death. Our primary endpoint is ***prevention of symptomatic COVID-19****. As there are currently no widely agreed definitions of 'symptomatic COVID-19', this sets out our definition that will be used in the trial analysis and the rationale for it.*

1. Symptoms and signs compatible with COVID-19 AND laboratory evidence of SARS-CoV-2 infection

The trial protocol stated symptomatic COVID-19 would be defined as 'symptoms compatible with COVID-19 AND laboratory evidence of SARS-CoV-2 infection'. We will include self-recorded temperature in the assessment thereby adjusting the definition to 'symptoms ***and signs*** *compatible with COVID-19 AND laboratory evidence of SARS-CoV-2 infection'. The decision to add temperature criteria was based on the fact that although COVID-19 can have protean manifestations it is primarily a febrile acute respiratory infection.*

2. Definition of symptoms used in the "symptomatic COVID-19" definition

Recently research has been published linking symptoms with the likelihood of a positive diagnosis of COVID-19 in the UK population(1). The symptoms of 1. sore throat, 2. cough, 3. shortness of breath, 4. muscle aches (severity not specified), 5. headache, 6. abdominal pain, 7, nausea, 8. vomiting, 9. diarrhoea overlap between this research definition and the prespecified symptom list which participants in this study were asked about when they reported to be unwell. In addition, we captured symptoms of 10. runny nose, 11. only able to leave the bed or chair for short periods because of severe symptoms, 12. going to hospital for treatment, and 13. admitted to hospital.

The UK symptom review (1) also captured loss of taste or smell, and fatigue/ weakness, and reported fever (as opposed to documented fever which we captured with oral temperature) which demonstrated good specificity for COVID-19 in this population. Thus 13. loss of taste or smell, 14. fatigue/ weakness, and 15. reported fever have been added as symptom categories, from entries documented in the free text box. These are the symptoms which are used to define "symptomatic COVID-19".

In summary the list of symptoms we will use in the "symptomatic COVID-19" definition in the analysis are:

1. sore throat	*9. diarrhoea*
2. cough	*10. runny nose*
3. shortness of breath	*11. only able to leave the bed or chair for short periods because of severe symptoms*

4. muscle aches	*12. going to hospital for treatment and admitted to hospital.*
5. headache	*13. loss of taste or smell*
6. abdominal pain	*14. fatigue/ weakness*
7. nausea	*15. reported fever*
8. vomiting	

*We also documented four symptoms which may be associated with the medication but which were **not** used to define symptomatic COVID-19 (itching, skin rash, dizziness or visual disturbance).*

3. ***Definition of asymptomatic in the asymptomatic COVID-19 definition***

*Being **asymptomatic** is defined as 1. not reporting to be 'unwell' at all during the duration of the study, 2. not reporting being 'unwell' as a reason for not going to work, 3. not reporting having symptoms on the three scheduled occasions where the participant met with the study teams (on D30, D60 and D90), and 4. not reporting to be febrile (oral temperature of < 37.5°C) throughout or not being defined as symptomatic as defined above and below.*

4. ***Definition of symptomatic in the symptomatic COVID-19 definition***

*Being **symptomatic** is defined for the primary endpoint as:*

- ***A. EITHER reporting the pre-specified symptoms and signs on the mobile app eDiary, which were in keeping with COVID-19 (symptoms as above or reported fever)***
  or
  documented symptoms in keeping with COVID-19 in the free text box (e.g. "nasal congestion") BUT NOT itching, skin rash, dizziness or visual disturbance, which were included to capture predominantly medication-related side-effects
- ***B. OR a documented oral temperature of ≥ 37.5°C***
- ***C. OR was diagnosed with COVID-19 during the study (at the time, and the locations where the study was conducted, screening of asymptomatic individuals was not conducted, so a diagnosis of COVID-19 would only have been on the basis of symptoms in keeping with COVID-19 +/- laboratory confirmation)***
- ***D. OR symptoms reported at the D30, D60 or D90 consultation with the study team (although the nature of the symptoms was not detailed at these visits, it was assumed these symptoms were significant enough to warrant mention at the consultation)***
- ***E. OR because a swab was done, or suggested to be done, by the study team.***

*If any of the criteria A to E listed above are met, the participant is classed as symptomatic **UNLESS** the participant documents in the free text box at the same time details (or an SAE report) that make COVID-19 or an ARI unlikely (e.g. Road Traffic Accident), in which case these episodes will be re-classified as asymptomatic. These free text symptoms will be assessed by two independent investigators blinded to treatment allocation, along with the other reported signs/ symptoms of the participant for that episode. Where there is disagreement in their assessment, a third blinded investigator will make the final decision.*

The protocol was designed to have the specific symptoms reported in the mobile eDiary app, and then for the participant to be assessed by the study team as to whether the reported symptoms were in keeping with COVID-19. Based on this assessment a swab would then be taken or not. Unfortunately, the assessments and swabbing practices proved highly variable between sites and periods. Some reasons for this were that certain countries could only perform swabbing with restrictive symptomatic

criteria (i.e. a continuous cough), or only those with highly likely COVID-19 were offered swabs. In some sites many participants (>25%) declined swabs or could not be contacted for review. To address the bias associated with variable practices across sites, the reported symptoms on the mobile app eDiary were used to determine whether a participant had asymptomatic or symptomatic COVID-19 supported by the face-to-face interviews at D30, D60 and D90 and recorded temperatures.

The criteria above should have high sensitivity for symptomatic COVID-19 and lower specificity, as symptoms may in cases be caused by a non-COVID-19 illness. The randomisation should balance across treatment arms, so the false positives (if there are any) will dilute the treatment effect (if there is one).

5. ***Pre-specified sensitivity analysis***

In addition to the above, a sensitivity analysis is planned using more stringent symptom definitions of:

- ***EITHER sore throat, runny nose, cough or shortness of breath (any), fever (reported or documented ≥37.5°C), loss of smell/taste, or a documented symptom in the free text corresponding to a URTI or LRTI ("nasal congestion", "flu") or another symptom <u>suggestive</u> of COVID-19 for <u>two out of three consecutive days</u>.***
- ***OR sore throat, running nose, cough or shortness of breath (any), fever (reported or documented ≥37.5°C), loss of smell/taste, for one out of three consecutive days, with another symptom reported on another occasion but within the same time window i.e. within 3 days (all symptoms mentioned in the primary endpoint 'symptomatic' definition, e.g. except itching, rash, dizziness or visual disturbance).***
- ***OR a documented diagnosis of COVID-19 in the CRF during the study in the absence of symptoms meeting the above criteria.***
- ***OR because a swab was done, or suggested to be done, by the study team.***

*If one of the listed symptoms/ signs were reported, the participant would be classed as symptomatic **UNLESS** the participant documents in the free text of the symptom diary details that make COVID-19 or an ARI unlikely (e.g. Road Traffic Accident), in which case these episodes will be re-classified as asymptomatic (see above).*

This sensitivity analysis may not capture all symptomatic cases of COVID-19 but has a-priori greater specificity, focussing on febrile acute respiratory illness symptoms, and for a greater duration.

B. The COPCOV testing algorithm steps (Figure 1)

1. ***Step 1: PCR Assessment: If a swab was done, this was because there were symptoms in keeping with COVID-19 (based on symptoms reported in the eDiary, or on discussion with the study team) and, as such, a SARS-CoV-2 PCR positive swab means that the participant had symptomatic COVID-19.***

*However, if the PCR was negative, this may have been because the patient did not have COVID-19, or that it was a false negative, or that the swab was taken outside the window of shedding of SARS-CoV-2. For those participants for whom the PCR was done and was negative, they will move to step 2 in the algorithm. Those participants who are PCR negative but seroconvert will have their cases reviewed by the **Serology Endpoint Assessment Committee (SEAC)** before the trial is unblinded (step 4). If influenza A, B or RSV were detected on the swab, and there were no other symptoms or fever recorded outside the window of +/- 7 days from the date of swab, the participant was classified as asymptomatic, on the basis that their symptoms were most likely caused by the detected virus. In all cases referred to the SEAC, the trial outcome recorded in the database will be the outcome determined by the SEAC.*

2. ***Step 2: Serum Serology Assessment (S-protein IgG assay): The study recruited from April 2020 and the last patient last visit was in March 2022. A previous diagnosis of COVID-19 and vaccination were exclusion criteria at enrolment. As such, the majority of people who contracted***

COVID-19 in the study will likely have been seronegative at baseline early on in the trial. For those who were seronegative (defined by the cut-offs established for this assay using extensive data from UK subjects and stated in the product insert for the Thermo Fisher OMNIPath Assay, i.e. ≤1,250ng/mL), a binary definition of seroconversion will be used (2).

This means a serum antibody titre of ≤1,250ng/mL at baseline and >1,250ng/mL at the end of the study (e.g. D90).

However, for cases where the binary threshold criterion was met but where there was a small total antibody rise (defined as <4 fold increase), these cases will be reviewed by the SEAC (before unblinding) to determine if this rise in antibody titre was in keeping with a new infection.*

For those participants who have a baseline serum antibody titre of >1,250ng/mL, which indicates previous exposure to SARS-CoV-2 spike protein antigen, a quantitative rise in antibody titre will be used to determine a new infection. The exact fold change in antibody titre which would indicate a new infection from baseline seropositivity has not been established previously, in particular in the populations in the COPCOV study. As such, an earlier recommendation from the SEAC was to use a conservative cut-off (i.e. one which maximises the capture of true positives, even if it produces some false negatives). The threshold decided upon by the SEAC was ***a ≥ 4-fold rise in antibody titre between D0 and the end of the study*** *(usually D90, but in some cases this could be earlier- if vaccinated, or later- if the D90 sample was not taken as described above)).*

3. ***Step 3: Dried Blood Spot (DBS) Serology Assessment (S-protein IgG assay): Serology is done primarily on serum samples but, where paired samples were unavailable, was performed also on dried blood spots. In the protocol, DBS were collected from all participants at D0, D30, D60 and D90 in the study. In the minority of cases where a D0 OR D90 serum sample was missing, for such cases all available DBS will be used for a serology test. Cut-offs based on DBS serology have not been established previously so these were derived from the current study by comparing simultaneous serum and DBS serology results. The cut-offs and criteria recommended by the SEAC are shown below. The window used for this seroconversion will be from the D0 Dried Blood Spot sample (or the first available sample) until the last available DBS sample (i.e. if a D0, D30 and D60 DBS is available, only the D0 and D60 will be used to determine seroconversion). When a D0 DBS is not available, but a D0 serum is available, the result will also be reviewed by the SEAC to determine if there was seroconversion.***

4. ***Blinded adjudication by the Serology Endpoint Assessment Committee (SEAC): In most cases the classification of trial outcome will be straightforward, but there will be a small number of cases where the algorithm and definitions leave uncertainty. The Serology Endpoint Assessment Committee (SEAC) will comprise experienced independent investigators with experience of COVID-19 clinical and laboratory diagnosis. Before the trial is unblinded the SEAC will be asked to determine for uncertain results from steps 1, 2 or 3, whether in their expert opinion, that the patient likely had COVID-19 or not. The trial report will identify which diagnoses of COVID-19 were determined by the SEAC. The blinded adjudication will be finalised (locked) before unblinding the treatment allocation.***

*Indicates that the above criteria were changed based on the SEAC assessment as documented below.

On review of the Diagnostic Algorithm by the SEAC:

The SEAC made the following minor suggestions to the endpoints before unblinding. The hierarchy is shown below:

1. PCR positive

2. Binary seroconversion on serum S-protein assay (≤1250ng/mL to >1250ng/mL, of which the large majority also had a 4-fold rise in titre)

3. A four-fold rise in serum antibody titre in those participants who were seropositive at baseline (based either on the 1:50 or 1:400 assay at 90 days)

4. DBS seroconversion. DBS derived serology results are systematically lower than those based on a serum assay. A regression analysis of simultaneous serum and DBS samples was used to derive the binary threshold value corresponding to the serum derived concentration of 1,250ng/mL of spike protein antibody (as in 2.). The corresponding value derived for the cut-off was 196ng/mL (when the starting value was corrected to 0). Thus, a conversion from ≤196ng/mL to >196ng/mL (of which the large majority also had a 4-fold rise in titre) was used to correspond with the serum criteria shown above in 2.

5. A four-fold rise in DBS titre in those seropositive on DBS at baseline

In addition, the SEAC decided that some cases would not be not assessable if the samples needed to make the above assessments were missing. The serology result for a particular timepoint was excluded if the serum 1:50 dilution result was greater than 7,500ng/mL (the threshold at which the SOP required that the sample would be repeated at a 1:400 dilution) BUT the 1:400 result was not available or was below 2,500ng/mL, (a value considered by the SEAC to be unreliable).

The SEAC's classification for the serological endpoints based upon the above criteria was accepted and their judgements on the individual cases performed before unblinding are those that are included in the database.

Those cases where the SEAC adjustment to the original study algorithm classification are made are indicated in the database.

The secondary objectives include determining if chloroquine/ hydroxychloroquine:

- Attenuates the clinical severity of COVID-19 infection
- Prevents asymptomatic COVID-19
- Prevents and attenuates the severity of symptomatic all-cause acute respiratory illnesses (ARI)

The study is a double-blind, randomised, placebo-controlled trial conducted in healthcare settings, and those at risk of developing COVID-19 (henceforth the study population). It is described briefly as follows. After obtaining fully informed consent, we will recruit members of the study population who can be followed up reliably for up to 5 months. In Asia, participants will be randomised to receive either chloroquine or placebo (1:1 randomisation). In Europe and Africa participants will be randomised to receive either hydroxychloroquine or placebo (1:1 randomisation).

A loading dose of 10mg base/kg (four 155mg tablets of base for a 60kg subject), followed by 155mg base daily (250mg chloroquine phosphate salt or 200mg of hydroxychloroquine sulphate) will be taken for 90 days. Episodes of symptomatic respiratory illness, including symptomatic COVID-19, clinical outcomes, and asymptomatic infection with the virus causing COVID-19 will be recorded during the follow-up period.

If participants are diagnosed with COVID-19 during the period of prophylaxis, they will continue their prophylaxis unless advised to do so by their healthcare professional or the study team. They will be

followed up for at least 28 days from the start of illness (up until a maximum of 60 days if not recovered at 28 days).

The procedures for identifying a case and the subsequent isolation and management will follow local and national guidelines; this study will not interfere in the usual local investigation and management of COVID-19 cases. Chloroquine and hydroxychloroquine have very few drug-drug interactions and should not interfere with the management of pneumonia.

Statistical Analysis Considerations

1.3 General Analysis Approach

The main analysis for the primary outcome will be in the intention-to-treat (ITT) population.

In this analysis, participants will be analysed according to the arm of randomisation irrespective of the treatment that was actually given and participant adherence to study drug. The ITT population includes all the participants that had either PCR, or Serology (serum) or Serology (dry blood spot (DBS)) endpoint result.

Clinical and diagnostic knowledge is informative for determining the result for uncertain serology outcomes. Thus, independent expert opinion (Independent serology endpoint assessment committee: SEAC) will be used to assist in assessing the data with uncertain outcomes based on their clinical, diagnostic and statistical/epidemiological knowledge. The number of uncertain endpoints will be clearly described in the trial reports.

These ITT analyses will be followed by the per protocol (PP) analysis.

A per-protocol (PP) analysis will be conducted to adjust for non-compliance to study protocols. Under an assumption of no post-randomisation confounding, this is a form of sensitivity analysis of the intention to treat analysis.

Inclusion in the per protocol analysis will require validation of the clinical data for each patient. Participants who did not receive the allocated study drug or who missed a significant number of doses, as defined as a major protocol deviation (>25%) in the study's SOP, will be excluded from the per protocol analysis.

Secondary endpoints will be analysed using the per protocol population. Data analysis will mainly be performed using R, Stata 17.0 or higher, StataCorp, 4905 Lakeway Drive College Station, Texas 77845 USA, Graphpad and other relevant software.

1.3.1 Missing data

The main ITT analyses will use cases with complete case outcomes from the PCR and Serology (serum) and Serology (dry blood spot (DBS) results. An independent endpoint review expert committee (SEAC) will provide their opinion on how to handle indeterminate outcomes from the PCR, serology (serum) and serology (DBS) results. Complete case analysis will be used to analyse the ITT endpoint (3-5). Those without outcomes will be excluded from this analysis but included in the sensitivity analysis as explained below.

For the participants without outcome data after the SEAC review, a sensitivity analysis will be considered using extreme case analysis (that they were COVID-19 negative) to impute the missing outcomes.

Study objectives and endpoints

1.4 Primary objective

- To determine if chloroquine/ hydroxychloroquine prophylaxis prevents symptomatic COVID-19 infection in the study population.

1.4.1 Primary endpoint

- The proportions of symptomatic COVID-19 infections will be compared between the chloroquine or hydroxychloroquine and placebo groups.

1.4.2 Secondary objectives and endpoints

- To determine if chloroquine/hydroxychloroquine prophylaxis attenuates the severity of COVID-19 infections.
- In all participants, the severity of infection will be compared between the two groups using a predefined severity score
- To determine if chloroquine/hydroxychloroquine prophylaxis prevents asymptomatic COVID-19 infection.
- The proportions of asymptomatic cases of COVID-19 will be determined by comparing acute and convalescent serology in the two groups.
- To determine if chloroquine/hydroxychloroquine prophylaxis prevents and attenuates all-cause symptomatic acute respiratory illnesses.
- The proportions and severity of symptomatic acute respiratory illnesses will be compared between the chloroquine/hydroxychloroquine and placebo groups.

1.4.3 Tertiary objective and endpoint

- To characterise genetic and baseline biochemical markers associated with symptomatic COVID-19, respiratory illness and disease severity
- Genetic loci and levels of biochemical components will be correlated with frequency of COVID-19, ARI and disease severity.
- To assess the impact of chloroquine or hydroxychloroquine prophylaxis on work and behaviour during the pandemic.
- The days lost to work, and the relationship between the subjective assessment of well-being and the decision to self-isolate when unwell (i.e. not go to work) will be examined in relation to the infection and treatment arm.
- To perform health economic analyses to assess the impact of chloroquine or hydroxychloroquine prophylaxis on costs and quality of life measures.
- The trial will collect data on use of health care resources and health related quality of life (EQ-5D-3L) to determine the effects between treatment groups.

Study design

The study is a double-blind, randomised, placebo-controlled trial that will be conducted in the study population. We will recruit participants, who can be followed reliably for up to 5 months. 40,000 participants were planned to be recruited and we predicted an average of 400-800 participants per site in 50-100 sites.

The participants will be randomised in Asia to receive either chloroquine or placebo (1:1 randomisation), and in Europe and Africa, to hydroxychloroquine or placebo (1:1 randomisation). The randomisation list will be prepared by the trial statistician using block randomisation and sent to the drug companies for drug packaging. The randomisation procedure will be stratified by site.

1.5 Determination of sample size

The sample size calculations performed at the beginning of the pandemic were based on an assumption of 3% incidence of symptomatic COVID-19 during the trial period. Expert opinion considers that if chloroquine/ hydroxychloroquine is effective, it may decrease symptomatic COVID-19 by approximately 23%, and therefore, the chloroquine/ hydroxychloroquine arm would have a 2.31% incidence of COVID-19 diagnosis. A 95% confidence interval with 80% power would require 8,520 subjects randomised to each arm. We aimed to enrol 10,000 subjects in each arm in the two trials which allows for a 20% LTFU, withdrawal rate, protocol deviation and non-adherence. Thus 20,000 would be randomised in Asia and 20,000 in Europe/Africa.

With repeated waves of COVID-19 during the study the sample size calculations have been updated during the study. The possibility of pooling data from chloroquine/ hydroxychloroquine arms (same mechanism of action) as well as with other similar studies, has justified continuation, although the original 40,000 recruitment was not feasible due to practical reasons.

Data Analysis

1.6 Trial Profile

The number of patients who will be screened, reasons for non-enrolment, number of patients randomised, number of patients lost to follow up and the number of patients assessed for the primary endpoint will be summarised in a CONSORT flow diagram, as shown in Figure 1, below.

Figure 1 Consort Trial Profile by Arms

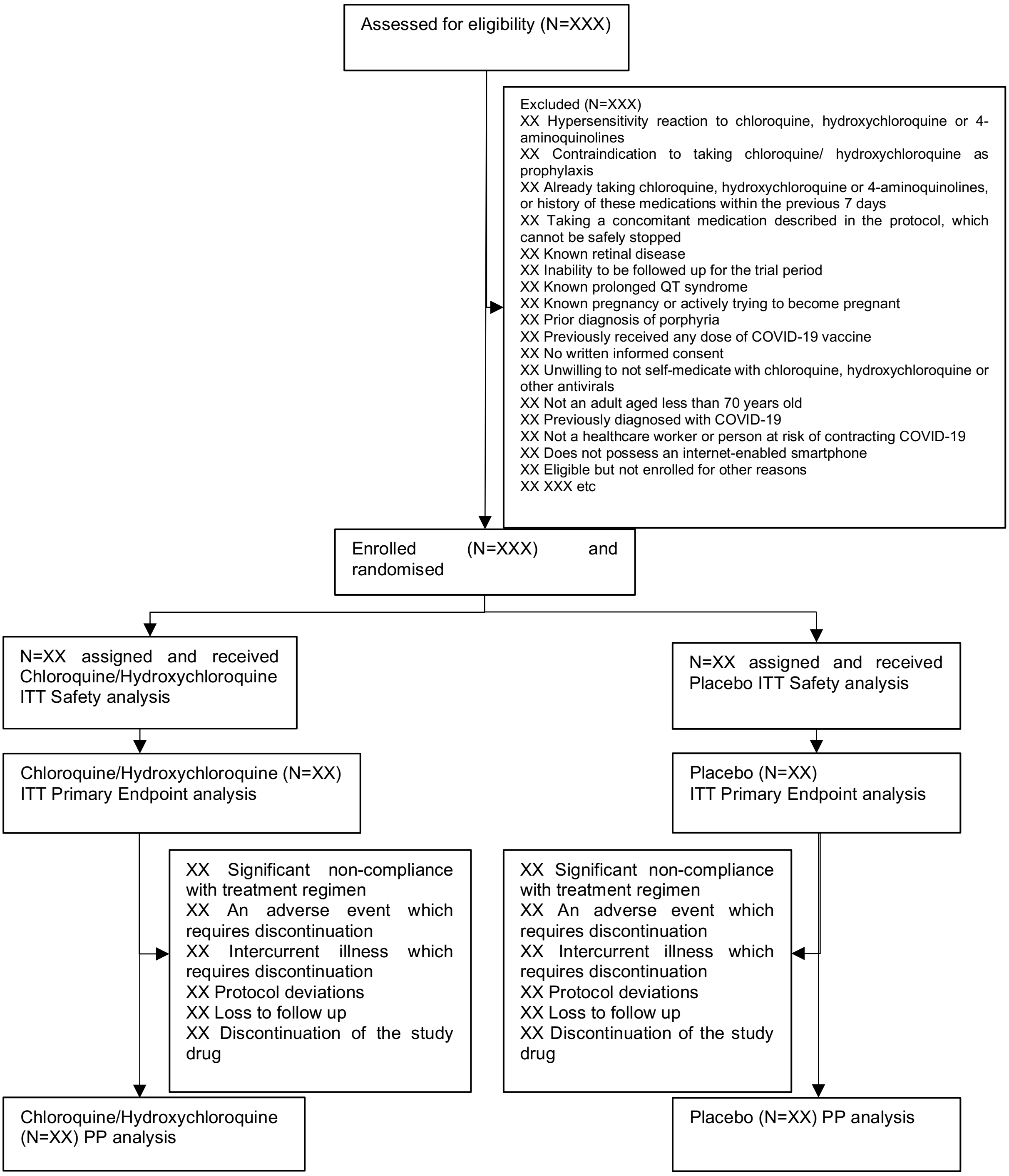

1.7 Demographics and other baseline characteristics

The following baseline characteristics will be described by study arm as shown in table 1 (below). Skewed continuous variables such as age will be summarised using medians with interquartile ranges (IQR). Continuous variables such as weight, height and temperature will be summarised using the mean with the standard deviation. Categorical variables such as sex, presence of fever, at baseline will be summarised using frequencies and percentages.

Table XXX. Baseline Characteristics of participants by study arm

**Characteristics**		**Chloroquine/Hydroxychloroquine (N=XX)**	**Placebo (N=XX)**
Age (years), med (IQR)		XX.X (XX.X-XX.X)	XX.X (XX.X-XX.X)
Sex	Male, n (%)	XX (XX.X)	XX (XX.X)
Oral Temperature °C, mean (SD)		XX (XX.X)	XX (XX.X)
Weight (kg), mean (SD)		XX.X (XX.X)	XX.X (XX.X)
Height (cm), mean (SD)		XX.X (XX.X)	XX.X (XX.X)
BMI **kg**/m^2			
Smoker	Yes, n (%)	XX (XX.X)	XX (XX.X)
COVID-19 in Household	Yes, n (%)	XX (XX.X)	XX (XX.X)
	No, n (%)	XX (XX.X)	XX (XX.X)
	Unknown, n (%)	XX (XX.X)	XX (XX.X)
**Existing co-morbidities**	Yes, n (%)	XX (XX.X)	XX (XX.X)
Chronic pulmonary disease (not asthma)	Yes, n (%)	XX (XX.X)	XX (XX.X)
Asthma (physician diagnosed)	Yes, n (%)	XX (XX.X)	XX (XX.X)
Chronic kidney disease	Yes, n (%)	XX (XX.X)	XX (XX.X)
Liver disease	Yes, n (%)	XX (XX.X)	XX (XX.X)
AIDS / HIV	Yes, n (%)	XX (XX.X)	XX (XX.X)
Diabetes	Yes, n (%)	XX (XX.X)	XX (XX.X)
Hypertension	Yes, n (%)	XX (XX.X)	XX (XX.X)
Cancer	Yes, n (%)	XX (XX.X)	XX (XX.X)
Condition requiring immunosuppressive	Yes, n (%)	XX (XX.X)	XX (XX.X)
Ischaemic heart disease	Yes, n (%)	XX (XX.X)	XX (XX.X)
High Cholesterol	Yes, n (%)	XX (XX.X)	XX (XX.X)
Other	Yes, n (%)	XX (XX.X)	XX (XX.X)
**Baseline symptoms**			
Fever	Yes, n (%)	XX (XX.X)	XX (XX.X)
Cough	Yes, n (%)	XX (XX.X)	XX (XX.X)

Sore throat	Yes, n (%)	XX (XX.X)	XX (XX.X)
Rhinorrhoea	Yes, n (%)	XX (XX.X)	XX (XX.X)
Wheezing	Yes, n (%)	XX (XX.X)	XX (XX.X)
Anosmia	Yes, n (%)	XX (XX.X)	XX (XX.X)
Chest pain	Yes, n (%)	XX (XX.X)	XX (XX.X)
Myalgia	Yes, n (%)	XX (XX.X)	XX (XX.X)
Arthralgia	Yes, n (%)	XX (XX.X)	XX (XX.X)
Shortness of breath on exertion	Yes, n (%)	XX (XX.X)	XX (XX.X)
Shortness of breath at rest	Yes, n (%)	XX (XX.X)	XX (XX.X)
Fatigue/ malaise	Yes, n (%)	XX (XX.X)	XX (XX.X)
Itching	Yes, n (%)	XX (XX.X)	XX (XX.X)
Headache	Yes, n (%)	XX (XX.X)	XX (XX.X)
Dizziness	Yes, n (%)	XX (XX.X)	XX (XX.X)
Visual Disturbance	Yes, n (%)	XX (XX.X)	XX (XX.X)
Abdominal pain	Yes, n (%)	XX (XX.X)	XX (XX.X)
Anorexia	Yes, n (%)	XX (XX.X)	XX (XX.X)
Nausea	Yes, n (%)	XX (XX.X)	XX (XX.X)
Vomiting	Yes, n (%)	XX (XX.X)	XX (XX.X)
Diarrhoea	Yes, n (%)	XX (XX.X)	XX (XX.X)
Rash	Yes, n (%)	XX (XX.X)	XX (XX.X)
XXXX etc	Yes, n (%)	XX (XX.X)	XX (XX.X)

1.8 Comparisons of incidence/risk rates of symptomatic COVID-19 infections between the chloroquine or hydroxychloroquine and placebo groups

The absolute numbers and the corresponding proportions (percentages) of symptomatic COVID-19 infections, asymptomatic COVID-19, (PCR-confirmed diagnosis, serology confirmed diagnosis (serum), serology confirmed diagnosis (dry blood spot (DBS)) will be presented as risks by treatment arm. A combined (symptomatic and asymptomatic COVID-19) outcome will be considered as an exploratory analysis. The proportions (usually expressed as percentages) will be summarised along with their 95% confidence intervals. The risk ratios and the corresponding 95% confidence intervals for the risk ratios will also be reported. If modelling will be required to adjust for relevant variables, a binomial regression model will be used to model the risk/ odds of symptomatic COVID-19 infection to obtain risk ratios/ odds ratios/ risk differences as appropriate comparing the chloroquine/ hydroxychloroquine arm with placebo. The risk ratios (or risk differences/ odds ratios) and the corresponding 95% confidence intervals will be obtained and reported. The primary measure will be the risk ratio. However, alternative measures such as odds ratios/risk differences may be considered if a log-binomial model does not converge (as is often encountered in practice) or if this trial's results

are combined in a meta-analysis with other trials that have reported these measures. Tests of significance for these analyses will be based on Fisher's exact test.

Where applicable and informative, graphical methods will be used to show trends in the incidence or probability of symptomatic COVID-19 over time, and by arm.

Survival methods may be used to estimate the time to COVID-19 infection as complementary approaches to the analysis if time to outcome data are available. In this approach, participants without outcomes will be censored at their longest observed time. Survival curves will be compared using the log-rank test. Where the survival approach is used as complementary analysis method, participants for whom study drugs are discontinued and/ or endpoints are not available due to other reasons (such as withdrawal from the study, loss to follow up) will be censored or treated as competing risks, as appropriate, from the moment of occurrence of one of these events in the ITT analysis. Survival plots may be informative for the PCR COVID-19 outcome if time to outcome data will be available.

Table 2 Outcomes of Chloroquine/Hydroxychloroquine Therapy for Pre-exposure Prophylaxis against COVID-19

Outcome	Chloroquine/ Hydroxychloroquine (N=XX)	Placebo (N=XX)	Risk ratios (95% CI)	P-value
Symptomatic COVID-19. n(%); 95%CI	XX (XX.X) (XX.X - XX.X)	XX (XX.X) (XX.X - XX.X)	XX (XX.X)	0.XXX
PCR-confirmed diagnosis. n(%); 95%CI	XX (XX.X) (XX.X - XX.X)	XX (XX.X) (XX.X - XX.X)	(XX.X - XX.X	0.XXX
Serology confirmed diagnosis (serum). n(%); 95%CI	XX (XX.X) (XX.X - XX.X)	XX (XX.X) (XX.X - XX.X)	XX (XX.X)	0.XXX
Serology confirmed diagnosis (DBS). n(%); 95%CI	XX (XX.X) (XX.X - XX.X)	XX (XX.X) (XX.X - XX.X)	(XX.X - XX.X	0.XXX
Asymptomatic COVID-19. n(%); 95%CI	XX (XX.X) (XX.X - XX.X)	XX (XX.X) (XX.X - XX.X)	(XX.X - XX.X	0.XXX
All cause respiratory illness. n(%); 95%CI	XX (XX.X) (XX.X - XX.X)	XX (XX.X) (XX.X - XX.X)	XX (XX.X)	0.XXX
Severity Score median (IQR)	XX (XX.X) (XX.X - XX.X)	XX (XX.X) (XX.X - XX.X)	XX (XX.X)	0.XXX

Supplementary figure XX

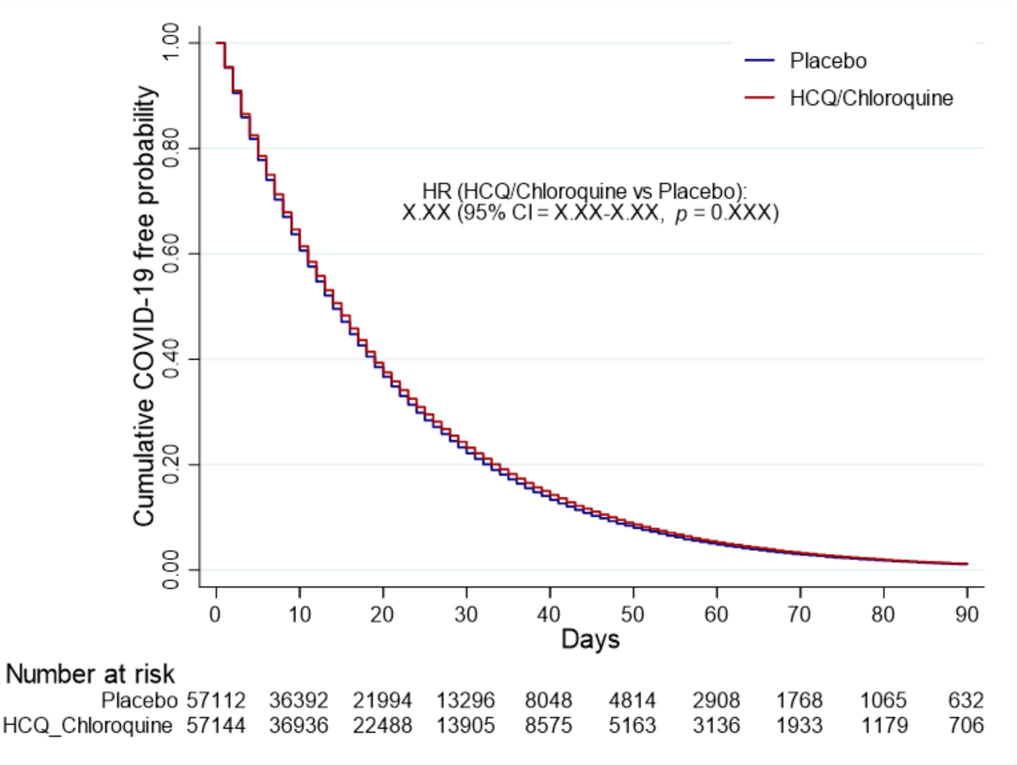

Figure XXX The Kaplan-Meier curves of cumulative COVID-19 free probability in the Chloroquine/ hydroxychloroquine (HCQ) or Placebo (NB: DUMMY Figure) for PCR confirmed COVID-19 (will appear in the supplementary materials if plotted).

1.9 Safety of Chloroquine/Hydroxychloroquine

Safety analyses will be based on the study intention to treat population. The safety and tolerability of chloroquine/ hydroxychloroquine vs placebo will be assessed by comparing the frequency of adverse events and serious adverse events, using Fisher's exact test, with particular attention to those adverse events that resulted in participants being unable to attend work or which interfered with the quality of life. Safety data will be presented in tabular and/or graphical format and summarized descriptively. AEs occurring in participants from enrolment and during trial participation (up until Day 150 for a sub-set of participants with extended follow-up) with a severity grade of 2 (moderate) or higher will be recorded. Adverse events will be graded according to the Common Terminology Criteria for Adverse Events (CTCAE) v5.0

The table below summaries the adverse events

Table XX Adverse events during follow up.

**Adverse events**	**Chloroquine/ hydroxychloroquine**		**Placebo**	
Number of subjects	XX		XX	
Serious adverse events (SAEs), n/N, (%)	XX/XXX (XX.X)		XX/XXX (XX.X)	
Deaths, n/N, (%)	XX/XXX (XX.X)		XX/XXX (XX.X)	
Possible, probable or definite drug related SAEs, n/N, (%)	XX/XXX (XX.X)		XX/XXX (XX.X)	
Grading of adverse events, n/N, (%)	2	3-4	2	3-4
**Symptoms, n/N (%)**	**X/XX (X.X)**	**X/XX (X.X)**	**X/XX (X.X)**	**X/XX (X.X)**
XXXXXXX	X/XX (X.X)	X/XX (X.X)	X/XX (X.X)	X/XX (X.X)
XXXXXXX	X/XX (X.X)	X/XX (X.X)	X/XX (X.X)	X/XX (X.X)
XXXXXXX	X/XX (X.X)	X/XX (X.X)	X/XX (X.X)	X/XX (X.X)
XXXXXXX	X/XX (X.X)	X/XX (X.X)	X/XX (X.X)	X/XX (X.X)
XXXXXXX	X/XX (X.X)	X/XX (X.X)	X/XX (X.X)	X/XX (X.X)
XXXXXXX	X/XX (X.X)	X/XX (X.X)	X/XX (X.X)	X/XX (X.X)
XXXXXXX	X/XX (X.X)	X/XX (X.X)	X/XX (X.X)	X/XX (X.X)
XXXXXXX	X/XX (X.X)	X/XX (X.X)	X/XX (X.X)	X/XX (X.X)
XXXXXXX	X/XX (X.X)	X/XX (X.X)	X/XX (X.X)	X/XX (X.X)
XXXXXXX	X/XX (X.X)	X/XX (X.X)	X/XX (X.X)	X/XX (X.X)
XXXXXXX	X/XX (X.X)	X/XX (X.X)	X/XX (X.X)	X/XX (X.X)
XXXXXXX	X/XX (X.X)	X/XX (X.X)	X/XX (X.X)	X/XX (X.X)
XXXXXXX	X/XX (X.X)	X/XX (X.X)	X/XX (X.X)	X/XX (X.X)
XXXXXXX	X/XX (X.X)	X/XX (X.X)	X/XX (X.X)	X/XX (X.X)

XXXXXXX	X/XX (X.X)	X/XX (X.X)	X/XX (X.X)	X/XX (X.X)
XXXXXXX	X/XX (X.X)	X/XX (X.X)	X/XX (X.X)	X/XX (X.X)
XXXXXXX	X/XX (X.X)	X/XX (X.X)	X/XX (X.X)	X/XX (X.X)
XXXXXXX	X/XX (X.X)	X/XX (X.X)	X/XX (X.X)	X/XX (X.X)

1.10 Meta-Analysis

Several other studies have reported randomised controlled trial (RCT) data on chloroquine/ hydroxychloroquine prophylaxis to prevent symptomatic COVID-19 infection. We plan to include in our report a meta-analysis of all hydroxychloroquine/chloroquine pre-exposure prophylaxis RCTs in COVID-19. Inclusion criteria for the studies are:

- **Pre-exposure studies;**
- **Pre-registered before trial start;**
- **Randomised controlled trials**

Primary endpoints and dose regimens will vary across the studies. For each included trial, we will use the individual study's pre-specified primary endpoint. We will conduct a fixed effect meta-analysis using the approach described in the meta-analysis reported by Garcia-Albeniz *et al*. (2022) (6). For cluster randomised trials, only estimates where clustering was explicitly accounted for in the analysis will be included.

A systematic review will be done in order to search for the relevant randomised trials with similar design and analysis approach to be included in the meta-analysis. We will use the WHO "living guidelines" to search for all relevant studies up until the reported search date. We will check Pubmed and EMBASE for trials or preprints reported since the last reported search by the WHO guidelines committee.

The meta-analysis will be presented as a forest plot. The I^2 statistic will be used characterise the percentage of total variation across studies resulting from trial heterogeneity rather than chance, with the p value of significance included. A funnel plot will be presented to assess publication bias.

1.11 Pharmacokinetic analysis of Chloroquine/Hydroxychloroquine

In the case that the primary endpoint of the study is met, a subset of patients in the chloroquine/hydroxychloroquine arms (approximately 10% of the total study population) will be randomly selected across the sites to have drug concentrations quantitated from their DBS samples in order to explore dose-response relationships between drug (or metabolite) exposure and the primary outcome (symptomatic COVID-19). There are three objectives for the pharmacometric data analysis:

- First, to verify that whole blood concentrations are within the expected range (related to published pharmacokinetic data from equivalent patient groups and dosing);
- Second, to explore whether there is any correlation between drug concentrations and outcome (symptomatic COVID-19) for those randomised to chloroquine/hydroxychloroquine;
- Third, to assess for baseline exposure to chloroquine/hydroxychloroquine in the study populations before enrolment into the study.

The first objective will use published pharmacokinetic data to assess whether drug levels of those in the chloroquine/hydroxychloroquine arms are within the expected range. For the second objective, participants who have D90 DBS, who are defined as meeting the primary endpoint (all, or a random subset, depending on how many) will be randomly matched with those participants who also have D90 DBS who did not develop symptomatic COVID-19. All DBS samples will be tested for drug levels for these participants but the D90 trough level will be used for the analysis. Significance is defined as a two-sided test at the 5% level significance. For the third objective, baseline DBS will be tested for chloroquine and hydroxychloroquine to indicate the prevalence of the use of these medications (these will be presented as a %, and reported in aggregate and per site).

1.12 Antibody response to vaccination for chloroquine/hydroxychloroquine

In those participants, who were vaccinated during the study and had antibody levels done pre-vaccine (or shortly afterwards as described in the protocol) and 28 days later, the antibody titre change will be compared between those in the chloroquine/hydroxychloroquine arm and the placebo arm, to determine if chloroquine/hydroxychloroquine attenuate the vaccine serological response.

Secondary objectives and endpoints

1.13 Comparison of severity and duration of COVID between the chloroquine/ hydroxychloroquine and placebo using a severity score.

A continuous severity score will be used to assess the severity of COVID-19, specifically taking into account the duration of illness. It has been adapted from the WHO Working Group on the Clinical Characterisation and Management of COVID-19 infection (7).

Each trial participant will be scored with a number varying from zero to 10^9 (death due to COVID-19). Participants who remain asymptomatic throughout the trial, are ill due to other reasons than COVID-19 or have asymptomatic COVID-19 infection (i.e. may have been symptomatic at a time that did not correlated with their COVID-19 diagnosis) will be assigned a score of zero implying that they all have tied ranks equal to 1 (Group 0).

We will use a rank-based mixed model approach to analyse these scores, stratified by site (hospital) (8). For site *i*, the Wilcoxon-Mann-Whitney estimate of the site-specific effect is denoted θ_i. We will use an ANOVA F test of the null hypothesis that $\theta_1=\theta_2=\ldots=\theta_N=1/2$ where N is the total number of sites (8).

1.13.1 Severity score table

Outpatient		
**Group**	**Score/day**	**Definition**
1	1	Feels unwell (reported on app but no specific symptoms)
2	5	Sore throat OR runny nose OR myalgia (not significantly limiting mobility) OR oral temperature ≥ 37.5°C
3	25	Cough
4	250	Only able to leave chair/bed for short periods (~15mins) due to severe symptoms
5	500	Shortness of breath on exertion
6	1,000	Shortness of breath at rest

Inpatient: hospitalised on clinical grounds (not for control/ isolation reasons)*		
**Group**	**Score/day**	**Definition**
7	10^4	Not requiring supplemental oxygen
8	10^5	SpO_2 < 94% (RA) or requiring** supplemental oxygen via face mask or nasal prongs
9	10^6	SpO_2 < 90% (RA) or requiring** supplemental high-flow oxygen or non-invasive ventilation
10	10^7	Requiring** intubation and mechanical ventilation
11	10^8	Ventilation and additional organ support (vasopressors, renal replacement therapy) or ECMO criteria met
12	10^9	Death

* Patient must be hospitalised due to illness severity (the opinion of the admitting physician is such that the patient cannot be safely managed out of hospital) and not for public health control/isolation reasons or legislation

** Either receipt of supplemental oxygen via this route OR in the opinion of the treating physician this was required but not administered (e.g. due to resource constraints)

The least severe symptomatic group (Group 1) is given a baseline score of 1 per day of symptoms (arbitrary choice, this does not influence the statistical analysis which is rank based). Each ordinal grouping, from Groups 2-12, is then assigned a relative increase in severity. For example, Group 2 is considered 5x worse than Group 1, whereas Group 5 is considered 100x worse than Group 2.

Scores are calculated on a daily basis (the average of the participants' two half-daily self-ratings via the mobile application). In cases of missing data between periods where symptoms were recorded, we will use linear imputation on the log10 scale. The final score of a symptomatic participant is defined as the sum of the scores for the period of symptoms associated with COVID-19.

By performing daily scoring with scores that are linear on a logarithmic scale, we can use the duration of symptoms to rank severity between participants, whilst ensuring that the most significant symptoms will dominate the final score.

For example, a participant who feels unwell for 3 days but no specific symptoms (Group 1), followed by 2 days of shortness of breath on exertion (Group 5) and then 4 days of cough (Group 3) would have a total score of 1x3 + 500x2 + 25x4 = 1003.

Table XX Comparison of severity scores between chloroquine or hydroxychloroquine and the placebo

**n/N, (%) Severity score**		**IRR**<br>**(XX% CI, p-value)**
Chloroquine/ Hydroxychloroquine<br>(N=XX)	Placebo<br>(N=XX)	
XX (XX.X)<br>(XX.X - XX.X)	XX (XX.X)<br>(XX.X - XX.X)	XX.X (XX.X - XX.X, 0.XXX)

1.14 Determining if chloroquine or hydroxychloroquine prophylaxis prevents asymptomatic COVID-19 infection.

The number of asymptomatic cases of COVID-19 will be determined by comparing baseline and end of trial serology between chloroquine/ hydroxychloroquine and the placebo arms (defined as those who seroconvert during the study (see Step 2 of the Primary Endpoint Assessment, but who do not meet the criteria for symptomatic COVID-19). The proportions will be obtained and risk differences along with the 95% confidence intervals will be calculated and reported.

Table XX Comparison of incidence rates of asymptomatic cases of COVID-19 with acute and convalescent serology between chloroquine or hydroxychloroquine and the placebo

**n/N, (%)**		**IRR**<br>**(XX% CI, p-value)**
Chloroquine/ Hydroxychloroquine<br>(N=XX)	Placebo<br>(N=XX)	
XX/XXX (XX.X)	XX/XXX (XX.X)	XX.X (XX.X - XX.X, 0.XXX)

1.15 Determining if chloroquine/ hydroxychloroquine prophylaxis prevents all-cause symptomatic acute respiratory illnesses.

The number and severity of symptomatic acute respiratory illnesses will be compared between the chloroquine/ hydroxychloroquine and placebo groups. The proportions will be obtained and risk differences along with the 95% confidence intervals will be calculated and reported to assess the difference in the proportions of symptomatic acute respiratory illnesses. Number will be assessed as per the methodology of **5.3**. Severity will be assessed as per the methodology of **6.1**. Subgroup analyses will occur for symptomatic acute respiratory illnesses excluding those with SARS-CoV-2 detected and in other individual respiratory viruses.

The severity of the symptomatic acute respiratory illnesses will be summarised as frequencies and percentages. The Poisson distribution will be used to calculate the incidence rates of severe symptomatic acute respiratory illnesses. The incidence rate ratios and the corresponding 95% confidence intervals will be obtained and used to compare the incidence rates of severe symptomatic acute respiratory illnesses between chloroquine/ hydroxychloroquine and the placebo. Tests of significance will be performed at 5% significance level.

Table XX: Comparison the proportion of symptomatic acute respiratory between the chloroquine or hydroxychloroquine and placebo groups.

**n/N, (%)**		**IRR**<br>**(XX% CI, p-value)**
Chloroquine/Hydroxychloroquine<br>(N=XX)	Placebo<br>(N=XX)	
XX/XXX (XX.X)	XX/XXX (XX.X)	XX.X (XX.X - XX.X, 0.XXX)

1.16 Tertiary objective and endpoint

- To characterise genetic and baseline biochemical markers associated with symptomatic COVID-19, respiratory illness and disease severity
- Genetic loci and levels of biochemical components will be correlated with frequency of COVID-19, ARI and disease severity.

- To assess the impact of chloroquine or hydroxychloroquine prophylaxis on work and behaviour during the pandemic.
- The days lost to work, and the relationship between the subjective assessment of well-being and the decision to self-isolate when unwell (i.e. not go to work) will be examined in relation to the infection and treatment arm.

A comparison of the mean number of days lost to work in the intervention and placebo arm will be made and presented with 95% Confidence Intervals (CI).

- To perform health economic analyses to assess the impact of chloroquine or hydroxychloroquine prophylaxis on costs and quality of life measures.
- The trial will collect data on use of health care resources and health related quality of life (EQ-5D-3L) to determine the effects between treatment groups.

- A Quality Adjusted Life Year (QALY) measurement (EQ-5D-3L) will be used to evaluate utility, by converting the EQ-5D-3L health states into utility scores using country/ region specific scoring algorithms. Where total costs are available or estimable, an incremental cost-effectiveness ratio will be calculated between the mean total costs and mean number of QALYs between the intervention and placebo groups. To account for the likely skewed nature of the cost data, the 95% confidence intervals (CI) for the mean differences between the intervention and control groups will be calculated. Where total costs are not estimable, or if the total costs are not available across a number of sites, making the analysis incomplete, the mean number of days lost to work due to being unwell or self-isolating, with the total days of hospitalisation and level of care required (i.e. requirement for standard hospital bed vs. Intensive Care Unit bed) will be compared to the mean number of QALYs. The analysis will be outlined in a separate health economics analysis plan.

References

1. Vihta KD, Pouwels KB, Peto TEA, Pritchard E, Eyre DW, House T, et al. Symptoms and Severe Acute Respiratory Syndrome Coronavirus 2 (SARS-CoV-2) Positivity in the General Population in the United Kingdom. Clin Infect Dis. 2022;75(1):e329-e37.
2. Wei J, Matthews PC, Stoesser N, Maddox T, Lorenzi L, Studley R, et al. Anti-spike antibody response to natural SARS-CoV-2 infection in the general population. Nat Commun. 2021;12(1):6250.
3. Groenwold RH, Donders AR, Roes KC, Harrell FE, Jr., Moons KG. Dealing with missing outcome data in randomized trials and observational studies. Am J Epidemiol. 2012;175(3):210-7.
4. Mukaka M, White SA, Terlouw DJ, Mwapasa V, Kalilani-Phiri L, Faragher EB. Is using multiple imputation better than complete case analysis for estimating a prevalence (risk) difference in randomized controlled trials when binary outcome observations are missing? Trials. 2016;17:341.
5. Sullivan TR, White IR, Salter AB, Ryan P, Lee KJ. Should multiple imputation be the method of choice for handling missing data in randomized trials? Stat Methods Med Res. 2018;27(9):2610-26.
6. Garcia-Albeniz X, Del Amo J, Polo R, Morales-Asencio JM, Hernan MA. Systematic review and meta-analysis of randomized trials of hydroxychloroquine for the prevention of COVID-19. Eur J Epidemiol. 2022;37(8):789-96.
7. Characterisation WHOWGotC, Management of C-i. A minimal common outcome measure set for COVID-19 clinical research. Lancet Infect Dis. 2020;20(8):e192-e7.
8. Boos DD, Brownie C. A rank-based mixed model approach to multisite clinical trials. Biometrics. 1992;48(1):61-72.
