# Supplementary material for: Evaluation of hydroxychloroquine or chloroquine for the prevention of COVID-19 (COPCOV): A double-blind, randomised, placebo-controlled trial
Source: PLoS Med. 2024 Sep 12;21(9):e1004428. doi: 10.1371/journal.pmed.1004428 (PMC11392261; doi:10.1371/journal.pmed.1004428)
Supplement: S1 Appendix — List of COPCOV study sites. Table A2. Baseline characteristics in the COPCOV trial (Per Protocol Analysis). Table A3. Outcomes of Chloroquine/Hydroxychloroquine and Placebo Pre-exposure Prophylaxis against COVID-19 in the COPCOV study (Per Protocol Analysis). Table A4. Summary of Serious Adverse Events in the COPCOV study. Table A5. Primary and secondary outcomes of Chloroquine/Hydroxychloroquine Therapy for Pre-exposure Prophylaxis against COVID-19 (missing outcomes treated as not having had COVID-19 during the study period) ITT–Results presented as “Risk differences.” Table A6. Outcomes of Chloroquine/Hydroxychloroquine and Placebo Pre-exposure Prophylaxis against COVID-19 in the COPCOV study (removing cases for which the SEAC judged that a study endpoint could not be determined). Table A7. Summary characteristics of previously published pre-exposure prophylaxis studies considered for meta-analysis. Table A8. Listing of causes of PCR-confirmed respiratory illness. Fig A1. Atlas showing those countries in which investigators were contacted to enquire whether they would be interested in, and able to join the COPCOV study. Fig A2. Atlas showing the location of the COPCOV trial sites which recruited participants, the 4-aminoquinoline tested, and the approximate numbers recruited. Fig A3. Graph showing cumulative enrollment over time (per week) by country. Fig A4. Funnel plot showing the 4-aminoquinoline COVID-19 pre-exposure chemoprevention RCTs included in the prespecified meta-analysis, and the relationship between point estimate risk ratios for the primary outcome and the corresponding standard errors. Fig A5. Meta-analysis of 4-aminoquinoline COVID-19 pre-exposure chemoprevention RCTs based on individual study primary endpoints using Risk Of Bias tool (RoB 2). Fig A6. Meta-analysis of the safety and tolerability outcomes in COVID-19 chemoprevention RCTs using the same methodology as reported in the WHO living guideline [24]. Fig A7. Meta-analysis of adverse events [file pmed.1004428.s007.docx]

Evaluation of hydroxychloroquine or chloroquine for the prevention of COVID-19 (COPCOV):

a double-blind, randomised, placebo-controlled trial

SUPPLEMENTARY APPENDIX

Contents

[Details of the COPCOV Collaborative Group and Acknowledgements 3](#_Toc172792844)

[Supplementary methods 11](#_Toc172792845)

[Protocol changes 11](#_Toc172792846)

[Methods A1 – Sample collection and processing 13](#_Toc172792847)

[Methods A2 – Meta-analysis of efficacy and treatment discontinuation 15](#_Toc172792848)

[Methods S3 – Interpretation of results 16](#_Toc172792849)

[Methods A4 – Symptom reporting and drug compliance via ePRO 17](#_Toc172792850)

[Methods A5 – Coding for the prespecified algorithm for determining the primary endpoint 18](#_Toc172792851)

[Supplementary Figures 19](#_Toc172792852)

[Figure A1 – Atlas showing those countries in which investigators were contacted to enquire whether they would be interested in, and able to join the COPCOV study 20](#_Toc172792853)

[Figure A2 – Atlas showing the location of the COPCOV trial sites, the 4-aminoquinoline tested (chloroquine or hydroxychloroquine), and the approximate numbers recruited 21](#_Toc172792854)

[Figure A3 – Graph showing cumulative enrollment over time (per week) by country 22](#_Toc172792855)

[Figure A4 – Funnel plot showing the relationship between estimated risk ratio and its standard error for the 4-aminoquinoline COVID-19 pre-exposure chemoprevention RCTs included in primary endpoint efficacy meta-analysis 23](#_Toc172792856)

[Figure A5 – meta-analysis of 4-aminoquinoline COVID-19 pre-exposure chemoprevention RCTs based on individual study primary end-points using Risk Of Bias tool (RoB 2) 24](#_Toc172792857)

[Figure A6 – Meta-analysis of the 4-aminoquinoline COVID-19 pre-exposure chemoprevention RCTs for the endpoint of PCR-confirmed symptomatic COVID-19 25](#_Toc172792858)

[Figure A7 – Meta-analysis of adverse events leading to treatment discontinuation reported in double-blind, placebo-controlled, 4-aminoquinoline COVID-19 pre-exposure chemoprevention RCTs 27](#_Toc172792859)

[Figure A8 – Graph showing cumulative loss to follow-up (LTFU) for Hydroxychloroquine/ Chloroquine arms and Placebo arm 28](#_Toc172792860)

[Supplementary tables 29](#_Toc172792861)

[Table A1 - List of study sites 30](#_Toc172792862)

[Table A2 – Baseline characteristics in the COPCOV trial (Per Protocol Analysis) 31](#_Toc172792863)

[Table A3 – Outcomes of Chloroquine/Hydroxychloroquine and Placebo Pre-exposure Prophylaxis against COVID-19 in the COPCOV study (Per Protocol Analysis) 33](#_Toc172792864)

[Table A4 – Summary of Serious Adverse Events in the COPCOV study 34](#_Toc172792865)

[Table A5 – Primary and secondary outcomes of Chloroquine/Hydroxychloroquine Therapy for Pre-exposure Prophylaxis against Covid-19 (missing outcomes treated as not having had covid-19 during the study period) ITT – Results presented as “Risk differences” 35](#_Toc172792866)

[Table A6 – Outcomes of Chloroquine/Hydroxychloroquine and Placebo Pre-exposure Prophylaxis against COVID-19 in the COPCOV study (removing cases for which the SEAC judged that a study endpoint could not be determined) 37](#_Toc172792867)

[Table A7 – Summary characteristics of previously published pre-exposure prophylaxis studies considered for meta-analysis ^7-18^ 38](#_Toc172792868)

[Table A8 – Listing of causes of PCR-confirmed respiratory illness 39](#_Toc172792869)

[References for COPCOV study supplementary materials 42](#_Toc172792870)

[List of legends for S1 Appendix 45](#_Toc172792871)

# Details of the COPCOV Collaborative Group and Acknowledgements

**Authors:**

William HK Schilling^1,2,*^, Mavuto Mukaka^1,2^, James J Callery^1,2^, Martin J Llewelyn^3,4^, Cintia V Cruz^1,2^, Mehul Dhorda^1,2^, Thatsanun Ngernseng^1^, Naomi Waithira^1,2^, Maneerat Ekkapongpisit^1^, James A Watson^2,5^, Arjun Chandna^2,6^, Erni J. Nelwan^7,8^, Raph L Hamers^2,9^, Anthony Etyang^2,10^, Mohammad Asim Beg^11^, Samba Sow^12^, William Yavo^13^, Aurel Constant Allabi^14^, Buddha Basnyat^2,15^, Sanjib Kumar Sharma^16^, Modupe Amofa-Sekyi^17^, Paul Yonga^18^, Amanda Adler^19^, Prayoon Yuentrakul^1^, Tanya Cope^1^, Janjira Thaipadungpanit^1,20^, Panuvit Rienpradub^1^, Mallika Imwong^1,21^, Mohammad Yazid Abdad^1,2^, Stuart D Blacksell^1,2^, Joel Tarning^1,2^, Frejus Faustin Goudjo^22^, Ange D. Dossou^23^, Abibatou Konaté-Touré^13^, Serge-Brice Assi^24^, Kra Ouffoué^25^, Nasronudin Nasronudin^26,27^, Brian Eka Rachman^26,27^, Pradana Zaky Romadhon^26,27^, Didi Darmahadi Dewanto^28^, Made Oka Heryana^28^, Theresia Novi^28^, Ayodhia Pitaloka Pasaribu^29^, Mutiara Mutiara^30^, Miranda Putri Rahayu Nasution^30^, Khairunnisa Khairunnisa^30^, Fauzan Azima Dalimunthe^29^, Eka Airlangga^31^, Akmal Fahrezzy^31^, Yanri Subronto^32^, Nur Rahmi Ananda^33^, Mutia Rahardjani^9^, Atika Rimainar^9^, Ruth Khadembu Lucinde^10^, Molline Timbwa^10^, Otieno Edwin Onyango^10^, Clara Agutu^10^, Samuel Akech^2,10^, Mainga Hamaluba^2,10^, Jairus Kipyego^18^, Obadiah Ngachi^18^, Fadima Cheick Haidara^12^, Oumar Y Traoré^12^, François Diarra^12^, Basudha Khanal^16^, Piyush Dahal^16^, Suchita Shrestha^15^, Samita Rijal^15^, Youssouf Kabore^34^, Eric Adehossi^35^, Ousmane Guindo^34^, Farah Naz Qamar^36^, Abdul Momin Kazi^36^, Charles J Woodrow^37,38^, Steven Laird^39^, Maina Cheeba^17^, Helen Ayles^17,40^, Phaik Yeong Cheah^1,2^, Walter RJ Taylor^1,2^, Elizabeth M Batty^1,2^, Kesinee Chotivanich^1,20^, Sasithon Pukrittayakamee^1,20^, Weerapong Phumratanaprapin^20^, Lorenz von Seidlein^1,2^, Arjen Dondorp^1,2^, Nicholas PJ Day^1,2^, Nicholas J White^1,2^ on behalf of the COPCOV Collaborative Group

1. Mahidol Oxford Tropical Medicine Research Unit, Faculty of Tropical Medicine, Mahidol University, Bangkok, Thailand
2. Centre for Tropical Medicine and Global Health, Nuffield Department of Medicine, University of Oxford, Oxford, UK
3. Department of Global Health and Infection, Brighton and Sussex Medical School, Brighton, UK
4. Department of Microbiology and Infection, University Hospitals Sussex NHS Foundation Trust, Brighton, UK
5. Oxford University Clinical Research Unit, Hospital for Tropical Diseases, Ho Chi Minh City, Vietnam
6. Cambodia Oxford Medical Research Unit, Angkor Hospital for Children, Siem Reap, Cambodia
7. Faculty of Medicine, Universitas Indonesia, Jakarta, Indonesia
8. Division of Tropical Medicine and Infectious Diseases, Department of Internal Medicine, Dr. Cipto Mangukusumo Hospital, Jakarta, Indonesia
9. Oxford University Clinical Research Unit Indonesia, Faculty of Medicine, Universitas Indonesia, Jakarta, Indonesia
10. KEMRI-Wellcome Trust Research Programme, Kilifi, Kenya
11. Department of Pathology and Laboratory Medicine, The Aga Khan University Hospital, Karachi, Pakistan
12. Centre pour le Développement des Vaccins (CVD-Mali), Bamako, Mali
13. Centre de Recherche et de Lutte contre le Paludisme, Institut National de Santé Publique, Abidjan, Côte d’Ivoire
14. Faculty of Health Sciences, Laboratory of Pharmacology and Toxicology, University of Abomey-Calavi, Cotonou, Benin
15. Oxford University Clinical Research Unit Nepal, Lalitpur, Nepal
16. B.P. Koirala Institute of Health Sciences (BPKIHS), Dharan, Nepal
17. Zambart, University of Zambia School of Public Health, Lusaka, Zambia
18. Fountain Health Care Hospital, Fountain Projects and Research Office (FOPRO), Eldoret, Kenya
19. Diabetes Trials Unit, Oxford Centre for Diabetes, Endocrinology and Metabolism, Radcliffe Department of Medicine, University of Oxford, Oxford, UK
20. Department of Clinical Tropical Medicine, Faculty of Tropical Medicine, Mahidol University, Bangkok, Thailand
21. Department of Molecular Tropical Medicine and Genetics, Faculty of Tropical Medicine, Mahidol University, Bangkok, Thailand
22. Coordination of Allada Ze Toffo Health zone, Adjian, Benin
23. National Public Health Laboratory, Cotonou, Benin
24. Institut Pierre Richet, Institut National de Santé, Publique, Bouaké, Côte d’Ivoire
25. Centre Hospitalier Universitaire (CHU) de Bouaké, Bouaké, Côte d’Ivoire
26. Faculty of Medicine, Universitas Airlangga, Surabaya, Indonesia
27. Universitas Airlangga Teaching Hospital, Universitas Airlangga, Surabaya, Indonesia
28. Husada Utama Hospital, Surabaya, Indonesia
29. Faculty of Medicine, Universitas Sumatra Utara, Medan, Indonesia
30. Murni Teguh Hospital, Medan, Medan, Indonesia
31. Bunda Thamrin Hospital, Medan, Indonesia
32. Department of Internal Medicine, Faculty of Medicine, Public Health And Nursing, Universitas Gadjah Mada/ Dr. Sardjito Hospital, Yogyakarta, Indonesia
33. Dr. Sardjito Hospital, Yogyakarta, Indonesia
34. Epicentre, Niamey, Niger
35. Université Abdou Moumouni de Niamey, Faculté des Science de la Santé, Niamey, Niger
36. Department of Paediatrics and Child Health, Aga Khan University Hospital, Karachi, Pakistan
37. Infectious Diseases department, Oxford University Hospitals NHS Foundation Trust, John Radcliffe Hospital, Oxford, UK
38. University of Oxford, Medical Sciences Division, John Radcliffe Hospital, Oxford, UK
39. University Hospitals of Coventry and Warwickshire NHS Trust, Coventry, UK
40. Clinical Research Department, Faculty of Infectious and Tropical Diseases, London School of Hygiene & Tropical Medicine, London, UK

* [william@tropmedres.ac](mailto:william@tropmedres.ac) (WHKS)

**Trial Steering Committee**

Nathalie Strub-Wourgaft (Independent Chair), Deborah Waller (Independent member), Attavit Asavisanu (Independent member), Nick Cammack (Wellcome observer, non-voting), Martin J Llewelyn (voting Investigator), Nicholas J White (voting Investigator) and William Schilling (Non-voting investigator)

**Independent Data and safety monitoring board**

Timothy Peto, Dennis Shanks, Sharon Kaur, James Tumwine and Sarah Walker

**Serology and Diagnostics Expert advice**

Timothy Peto, David Eyre, Susanna Dunachie, Andrew Simpson

COPCOV Trial Central Coordinating Office

**High-level support for finances, logistics, communication and strategy**

David Burton

**Data management**

Marja Schilstra, Montri Ridjaibun, Brian Mutinda, Paphapisa Chotthanawathit, Prapass Wannapinij

**Finance**

David Gandy, Noppamard Saowara, Vitalija Da Silva

**Logistics**

Winai Kaewkong, Soiratchaneekorn Ruanchaiman

**Ethics and Regulatory Submissions**

Salwaluk Panapipat, Orawan Anunsittichai, Varaporn Kruabkontho

**Monitoring and trial support**

Jaruwan Tubprasert, Akanittha Poonchai, Kittichai Theankham, Pongphaya Pongsuwan

**Statistical support**

Pimnara Peerawaranun

**Serology laboratory MORU (Mohammad Yazid Abdad and Stuart Dean Blacksell’s laboratory)**

Asama Vinitsorn, Phattaranit Tanunchai

**Molecular Microbiology laboratory (Janjira Thaipandungpanit’s laboratory- conducted SARS-CoV-2 and Respiratory Virus panel)**

Napasorn Prakthong, Mintra Thongyen

**Molecular Malaria laboratory (Mallika Imwong’s laboratory- conducted repeat SARS-CoV-2 and WGS on isolates)**

Kanokon Suwannasin, Wanassanan Madmanee

**MORU Public Engagement team**

Rita Chanviriyavuth (kindly took minutes for all COPCOV meetings), Natinee Kulpijit, Supa-at Asarath

**COPCOV Animation team (https://www.youtube.com/watch?v=wnWXuAvn8sw)**

Kanpong Boonthaworn, Ponlawoot Raksat, Puey Ounjai, Supa-at Asarath

**WWARN Sample management team**

Ranitha Vongpromek, Cholrawee Promnarate, Pak Jansawangkul, Phongtawee Thaweekan

**Communications and Newsletters**

John Bleho

**IT and Call Helpline support:**

Dean Sherwood, Nantawat Sriwattana, Diana Reyes Valencia, Adoracion Castillo Aranda

**Drug supply:**

Eric Bouilloux and Anthony Grosso from Accord Healthcare, which kindly donated the Hydroxychloroquine and matched placebo for the study and gave much support.

Local Clinical Centre COPCOV trial staff

*(listed in descending order of the number of patients randomised per country)*

**Indonesia**

Erni Nelwan (Country Co-PI), Raph L Hamers (Country Co-PI)

**Coordinating centre, OUCRU-ID, Jakarta (conducted monitoring):**

Kevin J Baird, Decy Subekti, Jeny Jeny, Winahayu Handayani, Fitria Wulandari, Nunung Nuraeni, Ichsan Kalbuadi, Novi Dwi Susilowati, Lia Waslia, Saraswati Soebianto, Mewahyu Dewi, Nurul Muyasaroh, Fitria Lestari

**Murni Teguh Hospital, Medan:**

Bangbang Buhari, Rico Andryan Simatupang, Eric Yudhianto, Muhammad Taqiyuddin Harahap, Rico Andryan Simatupang, Jesslyn Norberta

**Bunda Thamrin Hospital, Medan:**

Nita Aulia Nadana Lubis, Vincent Jimanto, Muhammad Anggi Ikhsan Siregar, Zakya Radita Nasution

**Husada Utama Hospital, Surabaya:**

Rony Agustian Hutri, Dwi Renti Astuti, Purw Purwaningsih, Wawan Riyanto, Mila Hestika Sari, Fitri Sei Linda

**UNAIR Hospital, Surabaya:**

Dia Rizka Isnawati, Soraya Isfandiary Iskandar, M. Robiul Fuadi, Arina Dery Puspitasari, Rosita Prananingtias, Cahyo Wibisono, Roza Fitrianingrum

**Sardjito Hospital, Yogyakarta:**

B Hartopo, Siti Nur Rohmah, Utsamani Cintyamena, Pebriati Sumarningsih, F Linda Tri Pramatasari

**Pakistan**

Asim Beg (Country PI)

**The Aga Khan University Hospital, Karachi**

Dilshad Begum, Anum Hussaini, Sonia Qureshi, Faisal Mahmood

**Mali**

Samba Sow (Country PI)

**Hospital of Mali, Bamako**

**The Bamako Hospital of Dermatology, Bamako**

Mamadou Karim Toure, Garan Dabo, Awa Traoré

**Kenya**

Anthony Etyang (Country Co-PI), Paul Yonga (Country Co-PI)

**Mbagathi County Hospital (KEMRI- conducted monitoring), Nairobi and Fountain Healthcare Hospital, Eldoret**

**Data collection:**

Joshua Wambua, Lilian Mulemi, Jennifer Mikusa, Karoli Lwanga, Ann Mbugua, Allan Hyugah, Don Obote, Karen Kendi, Charity Magawi, Elizabeth Weya, Jay Berkley

**Laboratory:**

Anthony Muema, Jennifer Musyoki, Caroline Ngetsa, Gerishom Angote, Katunge Mutinda, Purity Karemi, Martin Mwangi

**Community Engagement:**

Betty Yeri, Salim Mwakulore, Betty Kalama, Joy Kiptim

**Mbagathi Hospital Administration:**

Loice Mutai, Caren Emadau

**KWTRP Clinical Trials Facility:**

Salome Chira, Marianne Munene

**Côte d’Ivoire**

William Yavo (Country PI)

**Centre de Recherche et de Lutte contre le Paludisme, Institut National de Santé Publique, Abidjan, Côte d’Ivoire**

Gnagne Akpa Paterne, Boro Yves Arnaud, Coulibaly Abdoulkarim,

**Centre Hospitalier Universitaire (CHU) de Bouaké**

Koné Djakaridja, Yapo Martine Tatiana, Angaman N’guetta, Koné Mathurin, Yoda Adama, Dongui Gérald

**Centre Hospitalier Universitaire (CHU) d'Angré**

Binan Yves, Diaby Yahaya, Baffon-Abena Clarisse

**Benin**

Aurel Constant Allabi (Country PI)

**Centre Hospital University of Abomey-Calavi & So Ava (CHUZ/AS), Abomey-Calavi and Hospital De Zone Allada, Allada, Benin**

Asand OSSENI , Assad BIO SYA, Myrlene GBEGBE

**Nepal**

Sanjib Sharma (Country Co-PI), Buddha Basnyat (Country Co-PI)

**B.P. Koirala Institute of Health Sciences (BPKIHS), Dharan**

Narayan Raj Bhattarai, Surendra Uranw, Mamit Rai, Akash Rai, Sangita Shah, Keshav Rai, Akshat Mishra, Sonika Bhattarai

**Zambia**

Modupe Amofa-Sekyi (Country Co-PI), Helen Ayles (Country Co-PI)

**ZAMBART, Lusaka**

Kalenga Mwila, Elizabeth Biemba, Royd Simunga, Bernadette Chanda

**U.K.**

Martin Llewellyn (Country PI)

**Coordinating centre, Diabetes Trial Unit, University of Oxford, Oxford**

**(**Amanda Adler lead), Jo Milton, Steve Richards, Fay Croft, Greig Dougall, Lynne Tucker, Anne Gilligan, Ada Tse

**Brighton and Sussex University Hospitals NHS Trust, Brighton**

PI Martin J Llewelyn, Mindy Clarke, Celia Richardson, Dominika Wlazly, Daniel Richardson

**Oxford University Hospitals NHS Foundation Trust, Oxford**

PI Charles J Woodrow, Musaiwale Kamfose, Rachel Greer, Hannah Chase

**University Hospitals Coventry and Warwickshire NHS Trust, Coventry**

PI Steven Laird, Abeesh Panicker, Cathleen Chan, Rhian Hughes

**Imperial College Healthcare NHS Trust, London**

PI Alan Winston O’Keefe

**Birmingham and Solihull Mental Health NHS Foundation Trust, Birmingham**

PI Manny Bagary

**The Dudley Group NHS Foundation Trust, Dudley**

PI Raganath Dujairajan

**Rotherham Doncaster and South Humber NHS FT, Doncaster**

PI Adrian Phillipson, Michael Seneviratne, Kevin Williamson, Adrianne Close

**University Hospitals Of Morecambe Bay NHS Foundation Trust, Cumbria**

PI Marwan Bukhari

**Thailand**

Weerapong Phumratanaprapin (Country PI)

**Faculty of Tropical Medicine, Mahidol University, Bangkok**

**Niger**

Eric Adehossi (Country PI)

**Hôpital Lamordé, Niamey**

Rebecca Grais, Abdoul-Moumouni Issa-Soumani

Further acknowledgements:

We are very grateful to the many other colleagues in the trial sites and the MORU network who made this study possible. In particular:

We thank the donor, the Wellcome Trust, for the opportunity to conduct the research and especially Nick Cammack for continued support and encouragement.

We thank Accord Healthcare for the donation of the original hydroxychloroquine and matched placebo, and support with the study.

Marc Pellegrini from the Walter and Eliza Hall Institute, University of Melbourne who kindly donated hydroxychloroquine and matched placebo to COPCOV, after the expiry of the original IMP and Rima Darwiche and the team at CPL Australia for doing a fantastic job at of re-labelling and distributing to sites.

We would like to acknowledge the advice and support of Prof A. John Camm and Prof William J McKenna who gave expert Cardiology input.

We thank Graeme Bilbe for his pharmaceutical and strategic advice.

We thank Axiom for their assistance and support.

We thank Piramal for the preparation and packaging of the hydroxychloroquine and placebos.

We are very grateful indeed to colleagues and sites that were not able to participate eventually in the COPCOV trial but performed substantial preparatory work:

**Bangladesh:**

Abul Faiz, Anirhuddha Ghose

**Croatia:**

Davorka Lukas

**Democratic Republic of Congo:**

Gaston Tona Lutete

**Ethiopia:**

Kassa Haile, Mekonnen Teferi

**Guatemala:**

Celia Cordon-Rosales, Ingrid Sajmola, Carlos Graziosa, Laura Grajeda

**Italy:**

Piero Olliaro, Dora Buonfrate

**Lao People’s Democratic Republic:**

Elizabeth Ashley, Mayfong Mayxay

**Malaysia:**

Jean-Michel Piedagnel, Sasikala Siva

**Thailand:**

Carlo Perrone, François Nosten, Cindy Chu

**Vietnam:**

Sophie Yacoub, Guy Thwaites

# Supplementary methods

## Protocol changes

Initially, in response to the rapidly spreading COVID-19 pandemic, the COPCOV study was focussed on healthcare workers (as they were considered at high risk and effective chemoprevention would benefit both them and the pandemic stressed health system). However, as the study progressed, and the pandemic came slowly under control, and many healthcare workers had already contracted COVID-19, the inclusion criteria were widened and exclusions around COVID-19 vaccination were added. In version 6.0 of the study protocol, individuals who were considered at risk of COVID-19 infection could be enrolled but those who had received any dose of a COVID-19 vaccine were excluded.

The original and final study protocol are included in the supplementary material to this publication, together with summaries of the changes made.

| **Amendment No.** | **Protocol Version No.** | **Date issued** | **Author(s) of changes** | **Details of Major Changes** |
| --- | --- | --- | --- | --- |
| 1 | 2.0 | 23 Mar 2020 | Dr Will Schilling, Prof Nick White | Study population increased to 40,000 |
|  |  |  |  | Hydroxychloroquine added as study drug for sites in Europe |
| 2 | 3.0 | 06 Apr 2020 | Dr Will Schilling, Prof Nick White | Co-primary objective related to symptom severity now secondary |
|  |  |  |  | Study population clarified as healthcare workers (other “high-risk” population removed) |
|  |  |  |  | To ensure that the primary outcome measures are clear, a table has been added: *Overview of primary endpoint ascertainment* |
|  |  |  |  | Procedures for follow up of participants if unwell clarified (multiple sections) |
|  |  |  |  | Provision added for sites to enrol up to 200 participants if agreed with MORU |
|  |  |  |  | Safety reporting procedures updated to acknowledge local reporting requirements |
|  |  |  |  | AE capture clarified to Grade 2 or above (per CTCAE scale) |
|  |  |  |  | The statistical analysis was clarified to explain that 1) randomisation will be stratified by site, 2) a mixed effects negative binomial regression model will be used for the primary outcome, and 3) a rank-based mixed model approach will be used to analyse the severity scores |
|  |  |  |  | Funding source were clarified as Wellcome Trust via COVID-19 Therapeutics Accelerator |
|  |  |  |  | Africa added as study location |
| 3 | 4.0 | 06 Apr 2020 | Dr Will Schilling, Prof Nick White | Tertiary outcome and procedures added for EQ-5D-3L assessment, to assess the potential impact of study drug prophylaxis on work or behaviour during the COVID-19 pandemic |
|  |  |  |  | Study population was further clarified as healthcare workers with direct patient contact |
| 4 | 5.0 | 30 Jun 2020 | Dr Will Schilling, Prof Nick White | Participant inclusion has been broadened to include individuals working in healthcare facilities who are not providing direct patient care |
|  |  |  |  | Additional exclusion criteria and prohibited medications added after regulatory discussions and concerns expressed re pregnancy and potential cardiotoxicity. Upper age for participation clarified as <70 years old |
|  |  |  |  | Clarification added in study design for participants to stop study medication if they are hospitalised due to COVID-19 illness |
|  |  |  |  | Addition of possible interim analysis to be completed by DSMB |
|  |  |  |  | Pregnancy reporting process added |
|  |  |  |  | Visit window expanded to 27-31 days |
| 5 | 6.0 | 13 Jan 2021 | Dr Will Schilling, Prof Nick White | Defined procedures for management of participants who may receive COVID-19 vaccination after enrolment |
|  |  |  |  | Clarified the potential exploratory objectives which may be included in analysis, e.g., pharmacokinetic analysis of study medication and evaluation of antibody response in vaccinated participants |
|  |  |  |  | Updated benefit and risk profile for study medications and design |
|  |  |  |  | Participant inclusion has been further broadened to include individuals otherwise defined by site investigator as at risk for COVID-19 infection |
|  |  |  |  | Exclusion criteria have been updated to clarify that participants may not be enrolled with prior COVID-19 vaccination or current use of chloroquine or hydroxychloroquine |
|  |  |  |  | Study procedures updated to allow once daily reporting of participant temperature and symptoms |
|  |  |  |  | Further defined categories meeting serious adverse events (SAEs) |
|  |  |  |  | Clarified management of participants who discontinue study medication early for follow up through D90 |

Note: The following additional changes have been implemented in each revision as necessary, and are not individually itemised:

1. Updated background regarding current COVID-19 situation and currently available preventive and treatment options.
2. Site list for potential participating countries.
3. Minor formatting and consistency corrections.

## Methods A1 – Sample collection and processing

Dried blood spots (DBS) were collected from all participants at all visits (D0, D30, D60 and D90) on Whatman 31Et Chr (chromatography paper) and were stored in individual, sealed plastic bags with a desiccant until analysis. Serum samples were collected, separated and stored at -80°C at enrollment and at the end of the study, i.e., at D90, or at the last visit in case the patient left the study earlier. Nose and throat swabs for viral PCR were collected from patients who reported symptoms and were also stored as soon as possible at -80°C. All samples were shipped on dry ice to the MORU Laboratory and were checked to confirm specimen identity and quality as per standardised criteria before distribution to the respective labs for testing.

**Serology samples**

Serum or DBS samples collected from enrolled patients at D0, D30, D60, D90 were tested for the presence and quantification of IgG antibodies against the SARS-CoV-2 spike protein using the high-throughput methodology validated by the laboratory of the United Kingdom Office of National Statistics.

All serum samples were initially diluted to 1:50 for the assay. Samples with measured antibody levels greater than the upper limit of accurate quantitation of the assay (7,500ng/mL) were diluted further 1:8 (i.e. final dilution of 1:400) and re-assayed. During assay validation it was determined that for serum samples, any result with a concentration greater than 7500 ng/mL, was re-assayed at 1:400 dilution but any of these re-assayed samples which gave results ≤33% of the lower range of expected values at 1:50 (i.e. <1,250ng/mL), were rejected, and results from the dried blood spot (DBS) were used instead, if available.

Whole blood from DBS samples was resuspended by punching three 3mm diameter paper circles, roughly equivalent to 4.5µL of serum, from the DBS into 300 µL of the provided sample diluent, resulting in a dilution of approximately 1:67.

**Serology assays**

The OmniPATH 384 Max Combi SARS-CoV-2 IgG ELISA Kits for quantitative antibody assays were procured directly from the manufacturer (Thermo Fisher Diagnostics B.V., Landsmeer, The Netherlands). These kits include internal calibrators. The high-throughput workflow integrated a CyBio FeliX liquid handling robot (Analytik Jena, Jena, Germany), a Biotek EL406 plate washer (Agilent Technologies, Bangkok, Thailand) and a MultiSkan Sky High plate reader with the associated SkanIt software (Thermo Fisher). The assays were performed according to manufacturers’ instructions. Final results for the quantitative assay were read at 450nm.

**Nucleic acid amplification tests for the detection of SARS-CoV-2**

All swabs collected from patients in all countries (except Indonesia) were tested for SARS-CoV-2 using an in-house triplex real-time reverse transcriptase polymerase chain reaction (RT-PCR) in Thailand. The primer and probe sets targeted sequences encoding the viral E and N2 proteins as well as the human RNAseP3 as an internal control.^1-3^ The triplex real-time RT-PCR assay was validated and compared to commercial kits from Da An Gene Co., Ltd. of Sun Yat-Sen University, China and from Sansure BioTech Inc., China. Those kits were in the Global Fund’s (GFATM) “List of SARS-CoV-2 Diagnostic test kits and equipment eligible for procurement according to Board Decision on Additional Support for Country Responses to COVID-19 (GF/B42/EDP11)” version 41, 30-Sep-2022. The results from the in-house real-time RT-PCR assay and the commercial kits were in almost perfect agreement (kappa=0.968: 95%CI=0.932-1.000). Study samples were tested using the qScript XLT 1-Step RT-qPCR ToughMix^®^ (Quantabio, Beverly, MA, USA) with the following: concentrations of primers (0.19, 0.25 and 0.07 µM) and probes (0.1, 0.1 and 0.75 µM) for E, N2, RNAseP3 respectively at an annealing temperature of 62.5 °C. The RNA extraction, reverse transcription, and amplification for all validation experiments and for study sample testing were performed on the integrated BD MAX^TM^ platform (validation and reaction details to be published separately). Respiratory virus panels were developed earlier by our colleagues in Toronto University.^4^ The assay was done in Thailand using the integrated BD MAX^TM^ platform for the following eight viral targets: influenza A virus, influenza B virus, respiratory syncytial virus, entero/rhinoviruses, human parainfluenza virus 2, human parainfluenza virus 3, adenovirus, and human metapneumovirus. In Indonesia, commercial laboratories performed the SARS-CoV-2 PCRs (GSI Lab, Indonesia), and the respiratory virus panel (Micro-UI, Indonesia).

All assays showing negative results from the initial in-house assays in Thailand were re-tested in the laboratory of the Department of Molecular Tropical Medicine, Faculty of Tropical Medicine, Bangkok, Thailand using the TaqCheck™ SARS-CoV-2 Fast PCR Assay (Applied Biosystems^®^, Thermo Fisher Scientific, Waltham, Massachusetts).

## Methods A2 – Meta-analysis of efficacy and treatment discontinuation

**Efficacy**

As pre-specified in the statistical analysis plan (SAP), a meta-analysis of all hydroxychloroquine/chloroquine pre-exposure prophylaxis RCTs in COVID-19 was conducted. Inclusion criteria for these studies were:

- Pre-exposure studies;
- Pre-registered before they started the trial;
- Randomised controlled trials.

Primary endpoints and dose regimens varied considerably across the studies, reflecting the changing contexts as the pandemic progressed. In the main efficacy meta-analysis, the individual study’s pre-specified primary endpoint is used.^5^ A secondary analysis was performed using PCR-confirmed symptomatic COVID-19 only (as used by the WHO living guideline: <https://www.who.int/publications/i/item/WHO-2019-nCoV-prophylaxes-2021-1>).

Of the 12, individually randomised, pre-exposure chemoprevention studies which met the inclusion criteria for the efficacy meta-analysis,^7-18^ Grau-Pujol et al.^15^ was excluded as there were no incident events in the hydroxychloroquine arm. All risk ratios used the intention to treat population.

**Safety**

As in the WHO living guidelines (<https://www.who.int/publications/i/item/WHO-2019-nCoV-prophylaxes-2021-1>), the number of adverse events leading to treatment discontinuations was also assessed. As reporting of adverse effects may be influenced by knowledge of the treatment, only randomised, double-blind, placebo-controlled trials were included in this analysis. Effects were estimated as odds-ratios as the overall event rate is <1%. For all included trials we used the intention to treat population as the denominator.

**Search strategy**

We included all trials which met our inclusion criteria identified by the WHO living guidelines (last search date 17 August, version 1 24^th^ March 2023 <https://www.who.int/publications/i/item/WHO-2019-nCoV-prophylaxes-2023.1>) and Garcia-Albeniz et al. (last search date 17^th^ August 2023).^5^ A search from March 2022 to 1^st^ August 2023 was done on PubMed and EMBASE using the terms “chloroquine”, “hydroxychloroquine”, and “COVID-19” and restricted to randomised control trials. Two reviewers independently screened all abstracts. Risk ratios for the primary efficacy endpoint and the total number of events for the safety endpoint were extracted by two reviewers independently. Disagreements were discussed and, if not resolved, were decided by a third reviewer.

**Analysis**

Meta-analytic risk ratios and forest plots were estimated and drawn using the R package meta version 4.19 in R version 4.0.2. The meta-analytic risk ratios were estimated under a common effects model. The meta-analysis function used to generate the forest plots estimates the standard error (and hence to study weight) from the width of the CI on the log scale and then re-calculates the CI relative to the point estimate provided. As such the confidence intervals reported in the primary publication may differ slightly. Two authors (JAW, JJC) conducted a risk of bias assessment using the version 2 (RoB 2) of the Cochrane Risk-of-Bias tool.^6^ The code and data are provided in the accompanying GitHub repository (which will be made available at publication).

## Methods A3 – Interpretation of results

The prespecified decision tree algorithm used to determine the COPCOV trial primary endpoint, and the sequence of serological assessment used in endpoint determination is shown in the statistical analysis plan (SAP: appendix 5). The code is provided in the accompanying GitHub repository (made available at publication).

*PCR positivity*

PCR positivity for SARS-CoV-2 is regarded as 100% specific for the primary endpoint as it was tested only in symptomatic patients, but it can have a lower sensitivity depending on the swabbing technique and viral load (in particular the interval between swabbing and symptoms reported varied considerably).

*Seroconversion 1. Serum assays*

The serological assay based on serum is based on the extensive experience of COVID-19 serological testing by the United Kingdom Office of National Statistics. Seroconversion based on the serum assays was a change in titre from <1250 to >1250 ng/mL (of which 95% also had a 4-fold rise in titre). A four-fold rise in serum antibody titre in those participants who were seropositive at baseline (based either on the 1:50 or 1:400 assay at end of study) was also considered to indicate seroconversion.

*Seroconversion 2. Dried blood spot assays*

In contrast with the serum-based assay the DBS derived serological assessment method has not been validated previously. A comparison of estimated titres across a range of antibody concentrations from simultaneously measured serum and DBS samples showed variation, but did not show systematic bias related to concentration, although the serological results from DBS were substantially less sensitive (as evidenced by the lower seroprevalence derived from DBS). The regression model with a forced intercept of 0 identified a DBS titre of 196 ng/mL as corresponding to 1,250 ng/mL (the seroconversion threshold used in the serum assay). Thus, for the seroconversion criteria, a binary change from <196 ng/mL to >196 ng/mL was used in the DBS assay, or a four-fold rise in DBS titre in those seropositive on DBS at baseline, so as to correspond to the serum criteria.

As the timing of seroconversion in relation to symptoms could not be ascertained reliably, seroconversion was considered less reliable than qPCR in determining the primary endpoint. Overall, before unblinding, the specificity of the qPCR derived trial endpoint was regarded as highest, and that of the DBS derived serological assessment the lowest, and this was reflected in the hierarchy in the prespecified algorithm in the SAP.

Although the trial was conducted in unvaccinated participants, a high proportion (70% in those with available and analysable serum at baseline) were seropositive at study entry indicating high rates of previous SARS-CoV-2 infection.

## Methods A4 – Symptom reporting and drug compliance via ePRO

A simple reporting mobile ‘phone application (ePRO, Axiom Real-Time Metrics) was used in the COPCOV study. ePRO is iOS/Android compatible and available through the Apple App Store/Google Play Store. Participants were assisted with downloading ePRO at the initial visit, selected their language of choice, and inputted their user credentials (subject ID) and password.


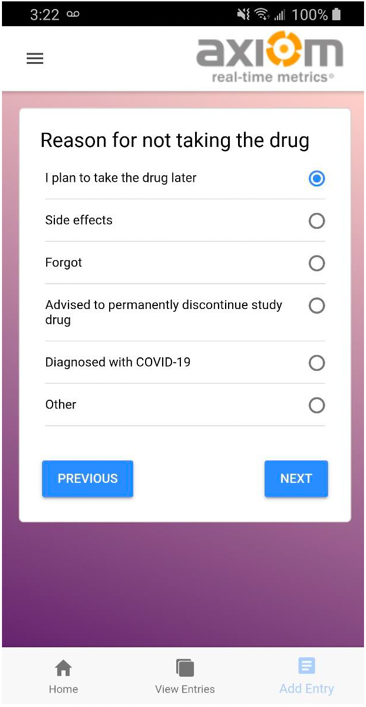

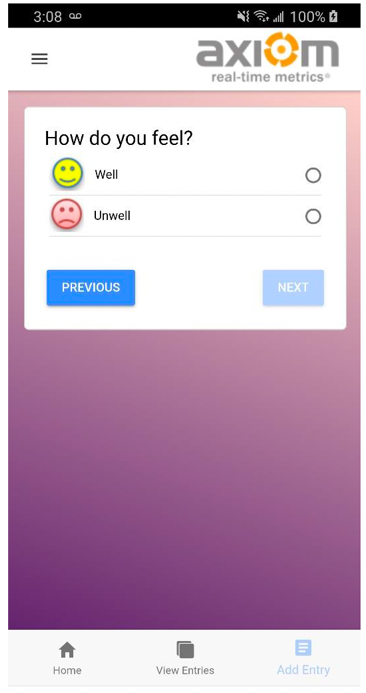

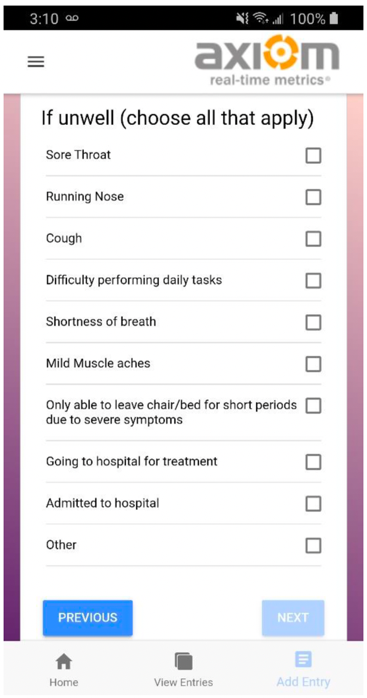
Participants recorded their temperature, symptoms and study drug intake daily via ePRO and received reminders via mobile phone notifications if this was not done.

Study nurses received real-time data via the Fusion eClinical Suite, allowing them to contact and further assess unwell participants. Nose and throat swabs were arranged if symptoms consistent with COVID-19 were present. Study drug compliance was also monitored via the Fusion eClinical Suite throughout the study.

Upon study completion, participant user accounts were deactivated, and participants were advised to delete the ePRO app from their phones.

## Methods A5 – Coding for the prespecified algorithm for determining the primary endpoint

Code for the prespecified algorithm for determining the serology component of the primary endpoint was implemented as an RMarkdown script (run using R version 4.2.2 with the R packages tidyverse, readxl, and lubridate). This is given as endpoint_review_algorithm.Rmd in the accompanying GitHub repository.

# Supplementary Figures

## Fig A1. – Atlas showing those countries in which investigators were contacted to enquire whether they would be interested in, and able to join the COPCOV study


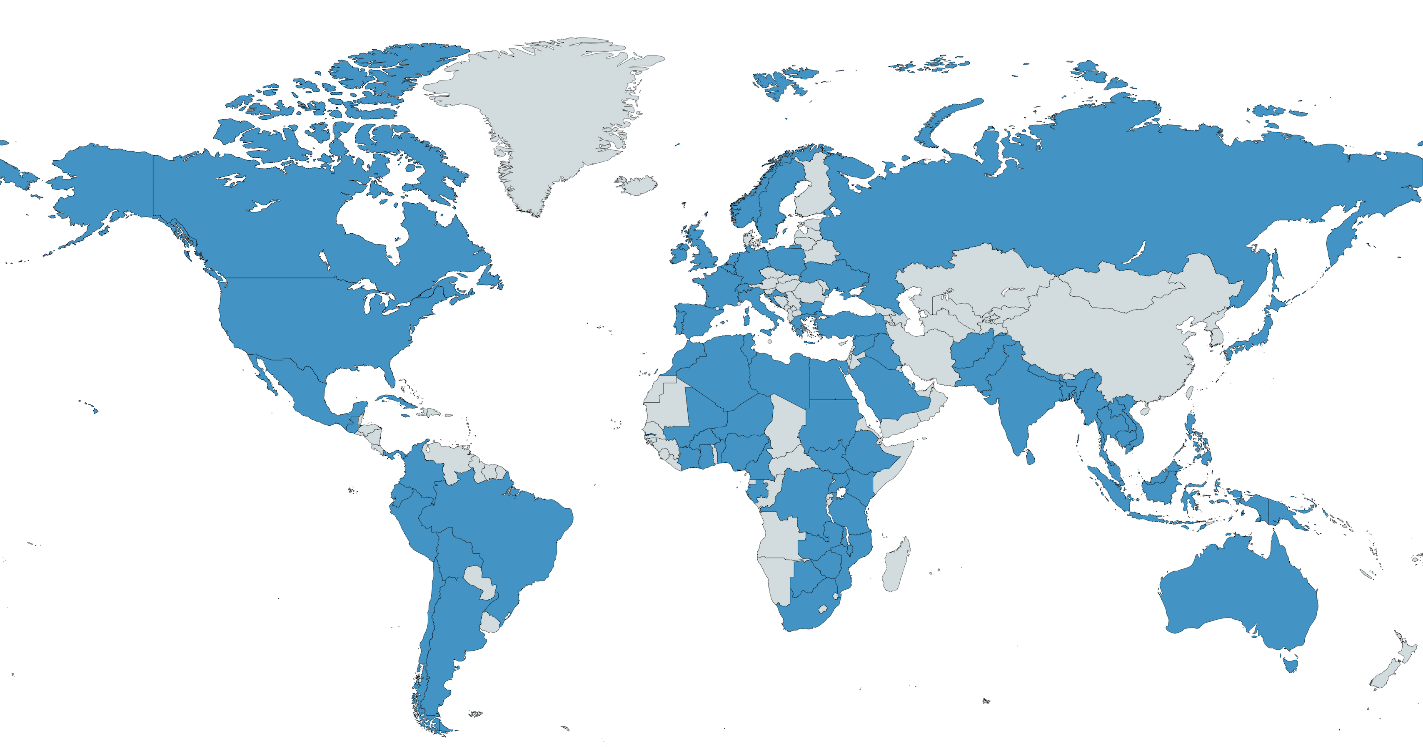


## Fig A2. – Atlas showing the location of the COPCOV trial sites, the 4-aminoquinoline tested (chloroquine or hydroxychloroquine), and the approximate numbers recruited


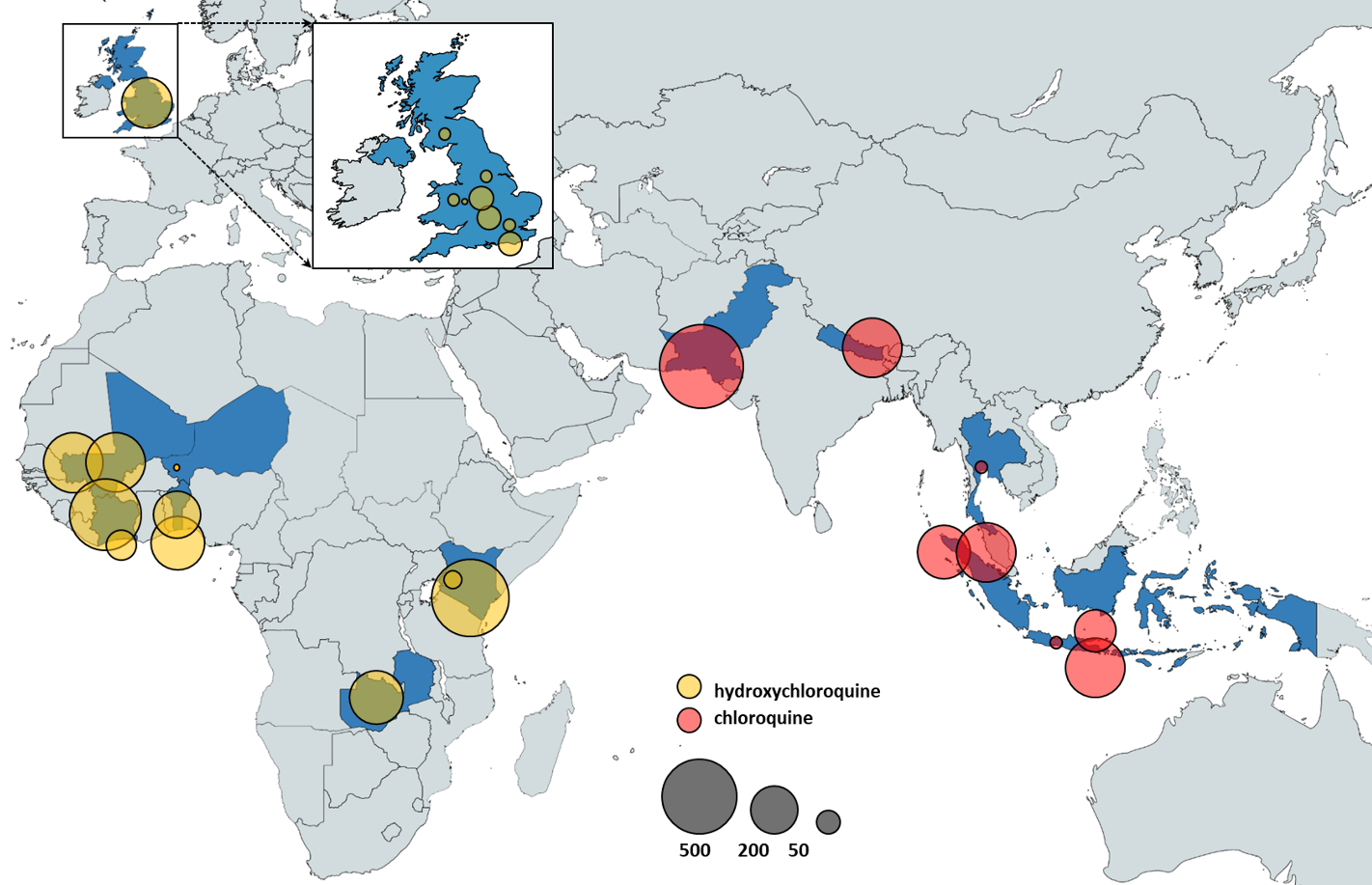


## Fig A3. – Graph showing cumulative enrollment over time (per week) by country


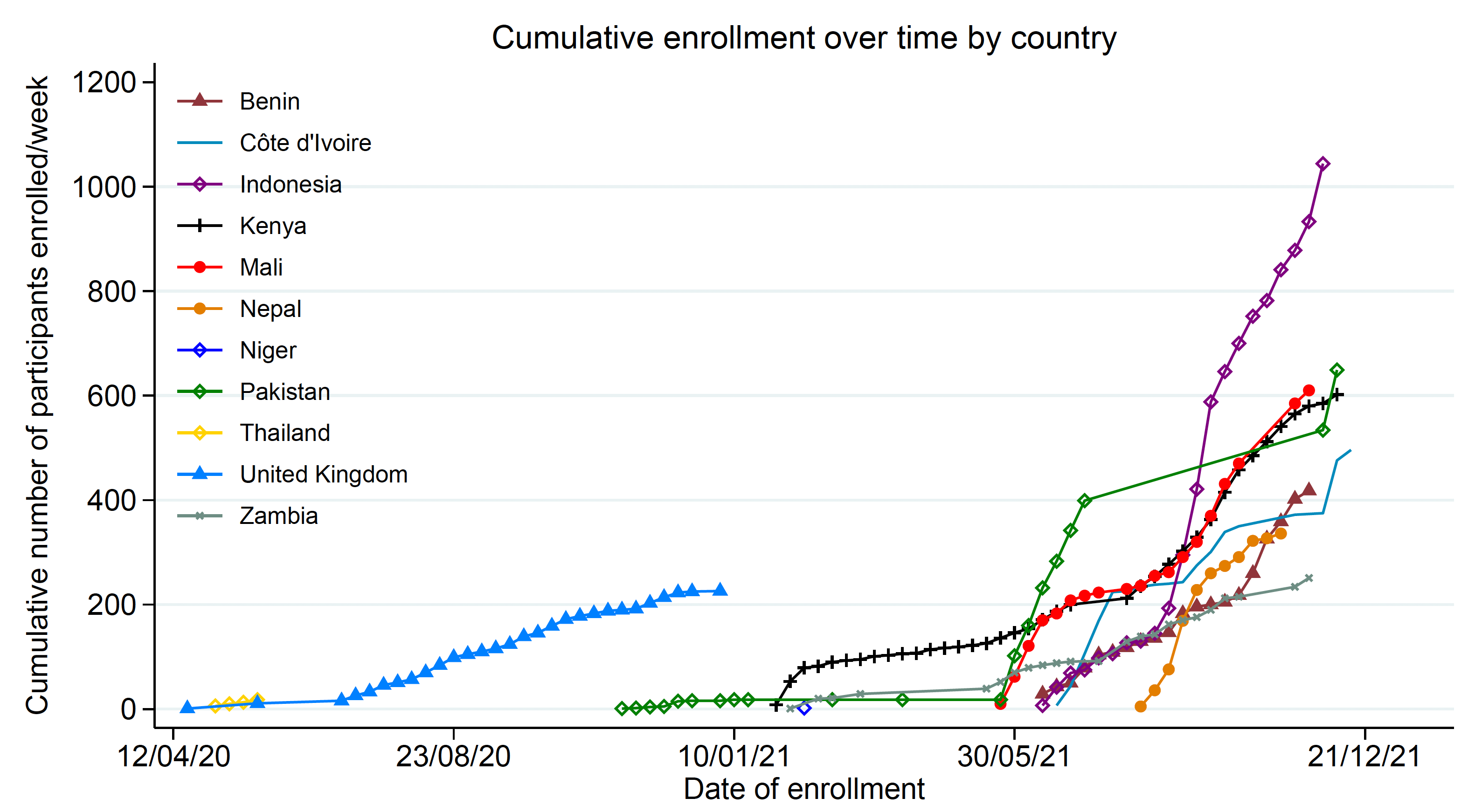


## Fig A4. – Funnel plot showing the relationship between estimated risk ratio and its standard error for the 4-aminoquinoline COVID-19 pre-exposure chemoprevention RCTs included in primary endpoint efficacy meta-analysis


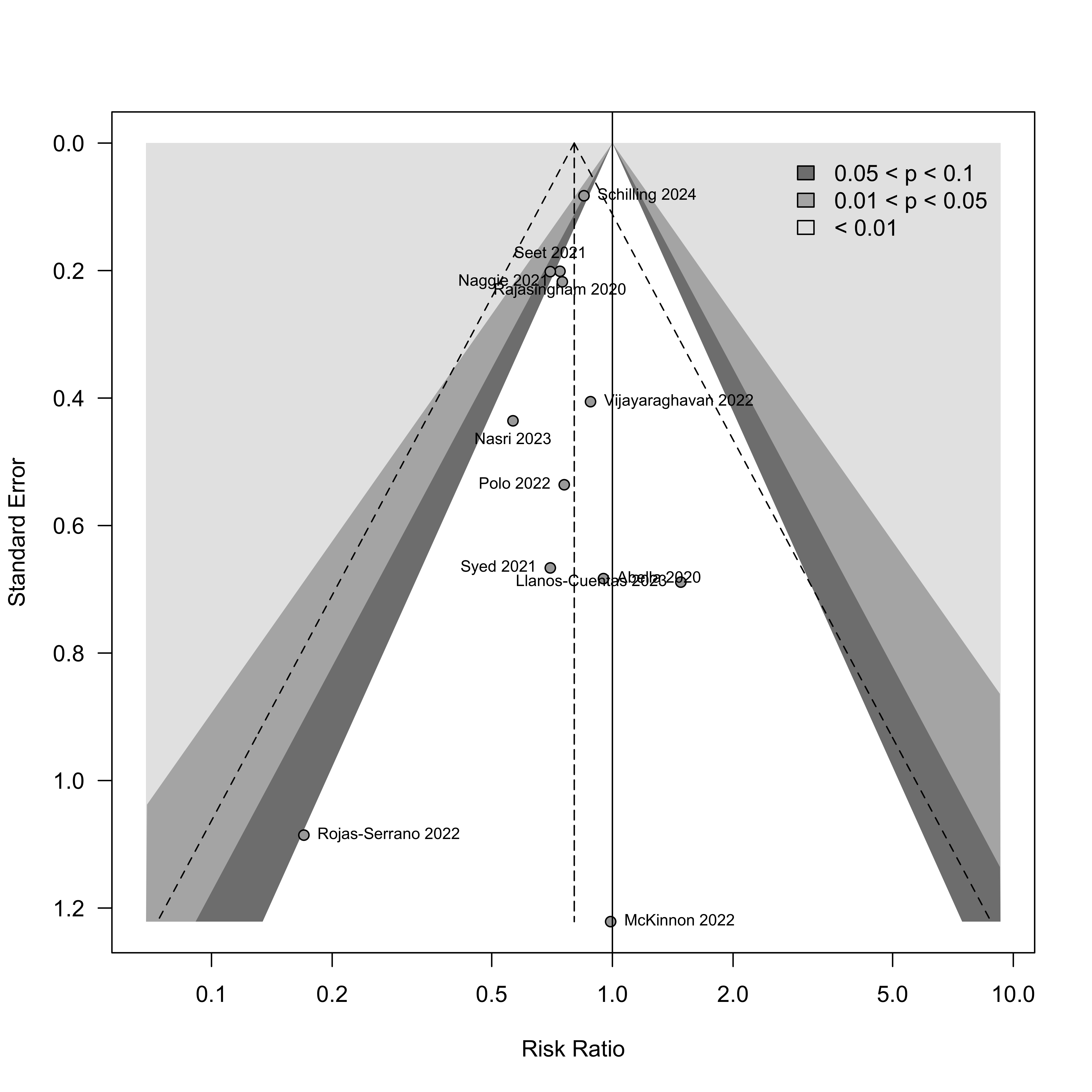


Assessment of possible publication bias by evaluating the relationship between the point estimate risk ratios and their corresponding standard errors for the hydroxychloroquine/chloroquine COVID-19 pre-exposure chemoprevention RCTs included in the primary endpoint efficacy meta-analysis.^7-18^ The x-axis denotes the risk ratio for the primary study endpoint on the log-scale and the y-axis shows the corresponding standard error. The vertical dashed line shows the summary meta-analysis risk ratio also shown in the main paper (Figure 3).

## Fig A5. – meta-analysis of 4-aminoquinoline COVID-19 pre-exposure chemoprevention RCTs based on individual study primary end-points using Risk Of Bias tool (RoB 2)


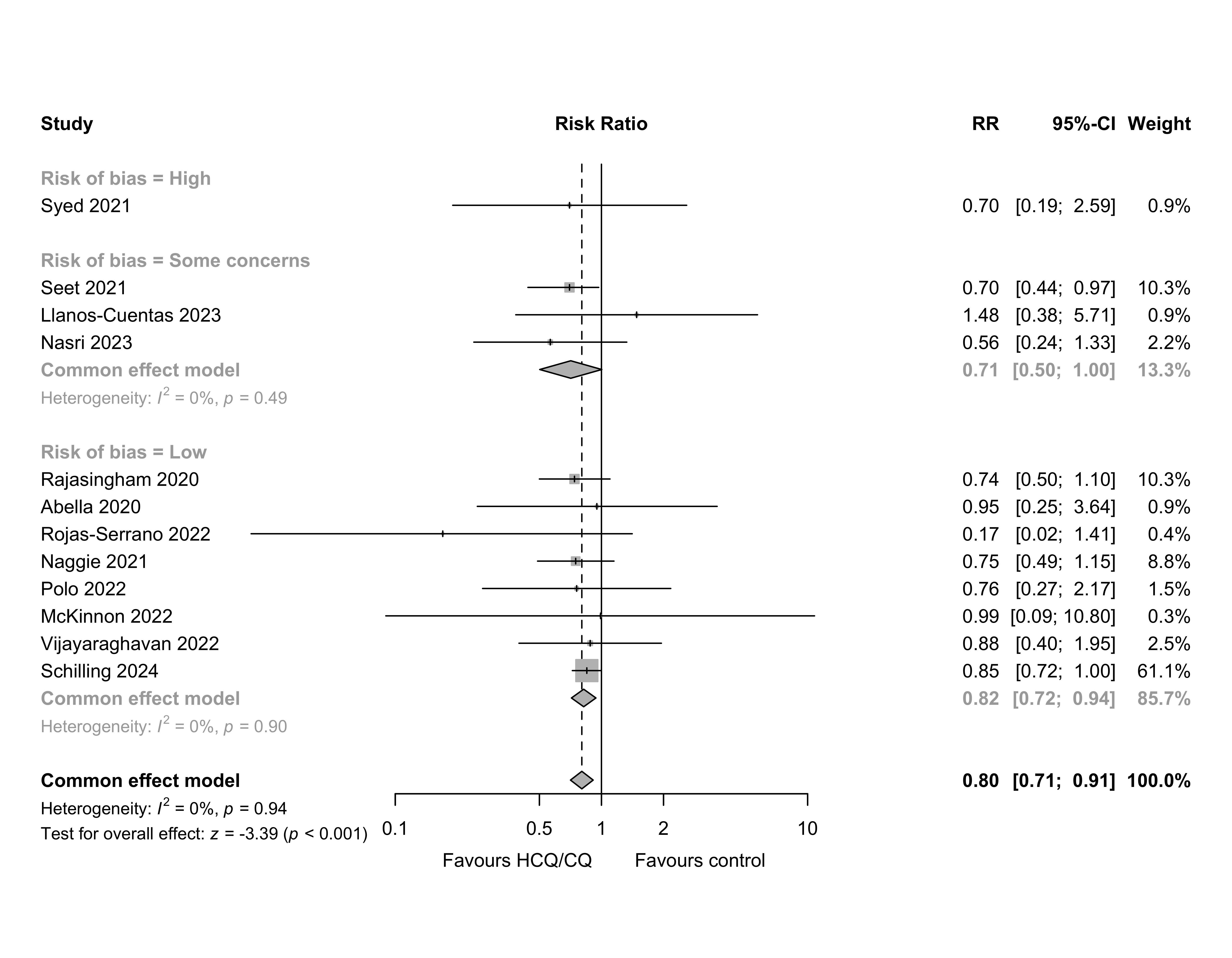


Hydroxychloroquine/chloroquine COVID-19 pre-exposure chemoprevention RCTs with Risk-of-Bias analysis. ^6-18^ Schilling 2024 refers to the current study.

## Fig A6. – Meta-analysis of the 4-aminoquinoline COVID-19 pre-exposure chemoprevention RCTs for the endpoint of PCR-confirmed symptomatic COVID-19


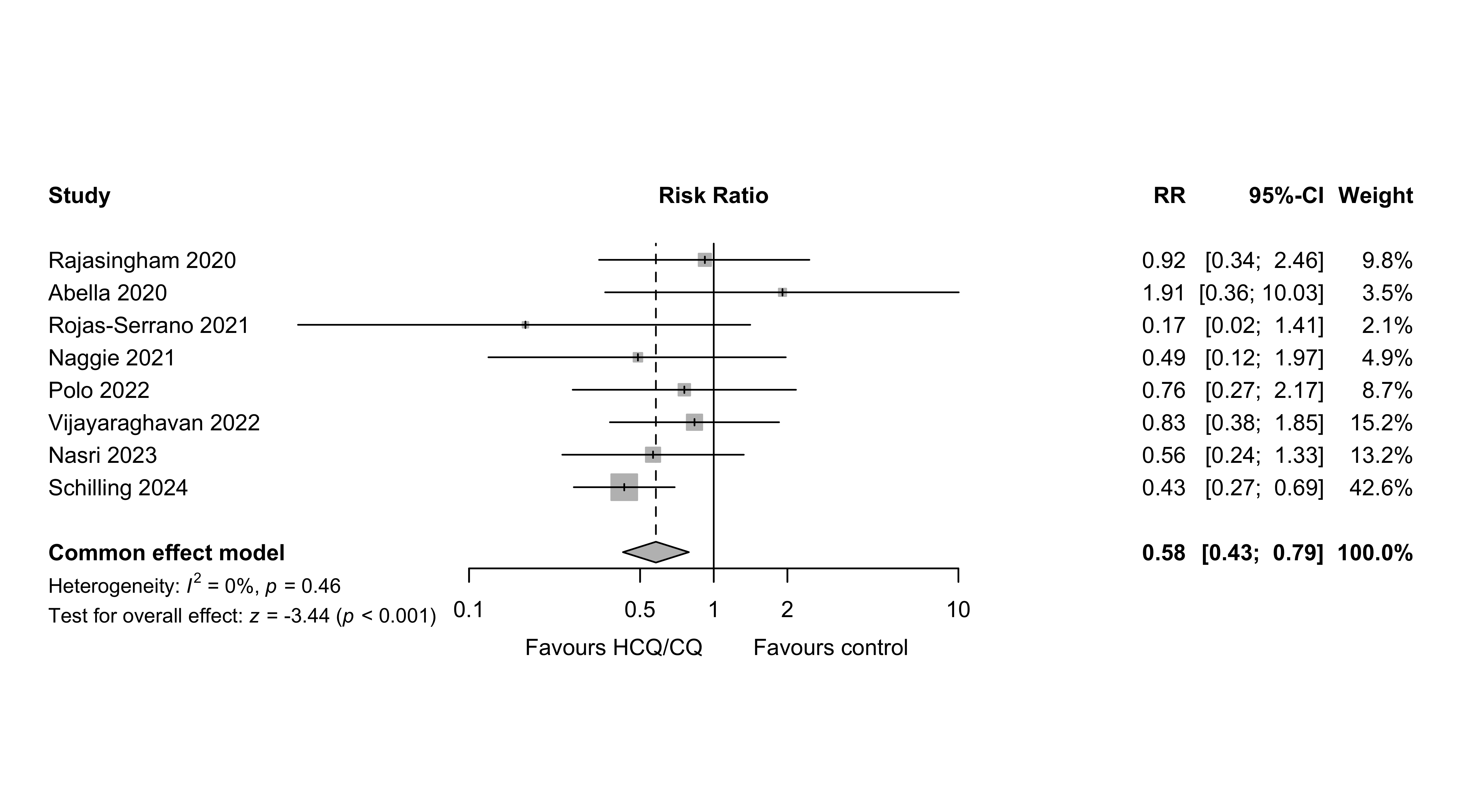


Hydroxychloroquine/chloroquine COVID-19 pre-exposure chemoprevention RCTs comparing rates of PCR-confirmed symptomatic COVID-19. ^7-18^ Schilling 2024 refers to the current study.

## Fig A7. – Meta-analysis of adverse events leading to treatment discontinuation reported in double-blind, placebo-controlled, 4-aminoquinoline COVID-19 pre-exposure chemoprevention RCTs


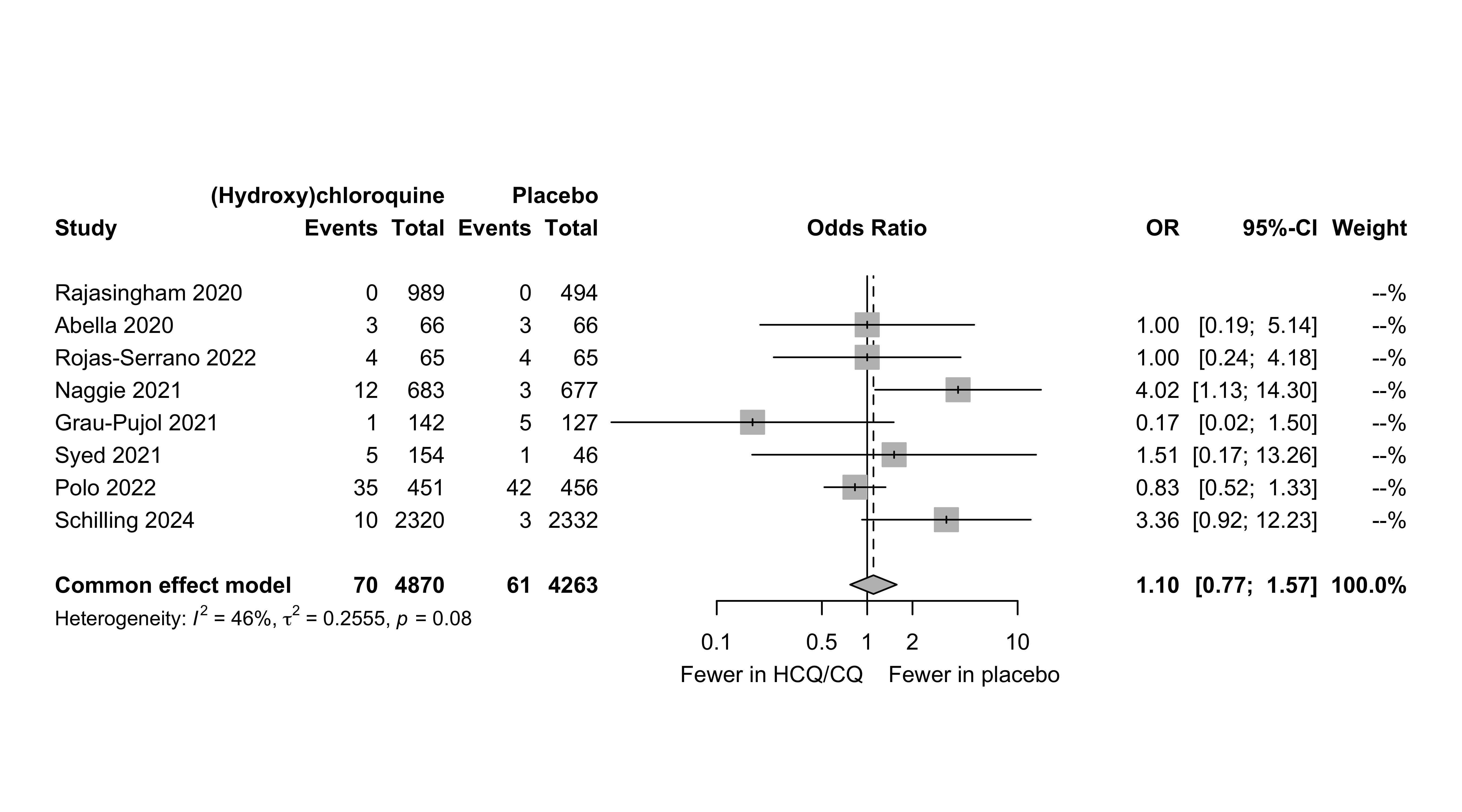


Adverse event rates leading to treatment discontinuation reported in the double-blind, placebo-controlled, hydroxychloroquine/chloroquine COVID-19 pre-exposure chemoprevention RCTs ^7-18^ Schilling 2024 refers to the current study.

## Fig A8. – Graph showing cumulative loss to follow-up (LTFU) for Hydroxychloroquine/ Chloroquine arms and Placebo arm


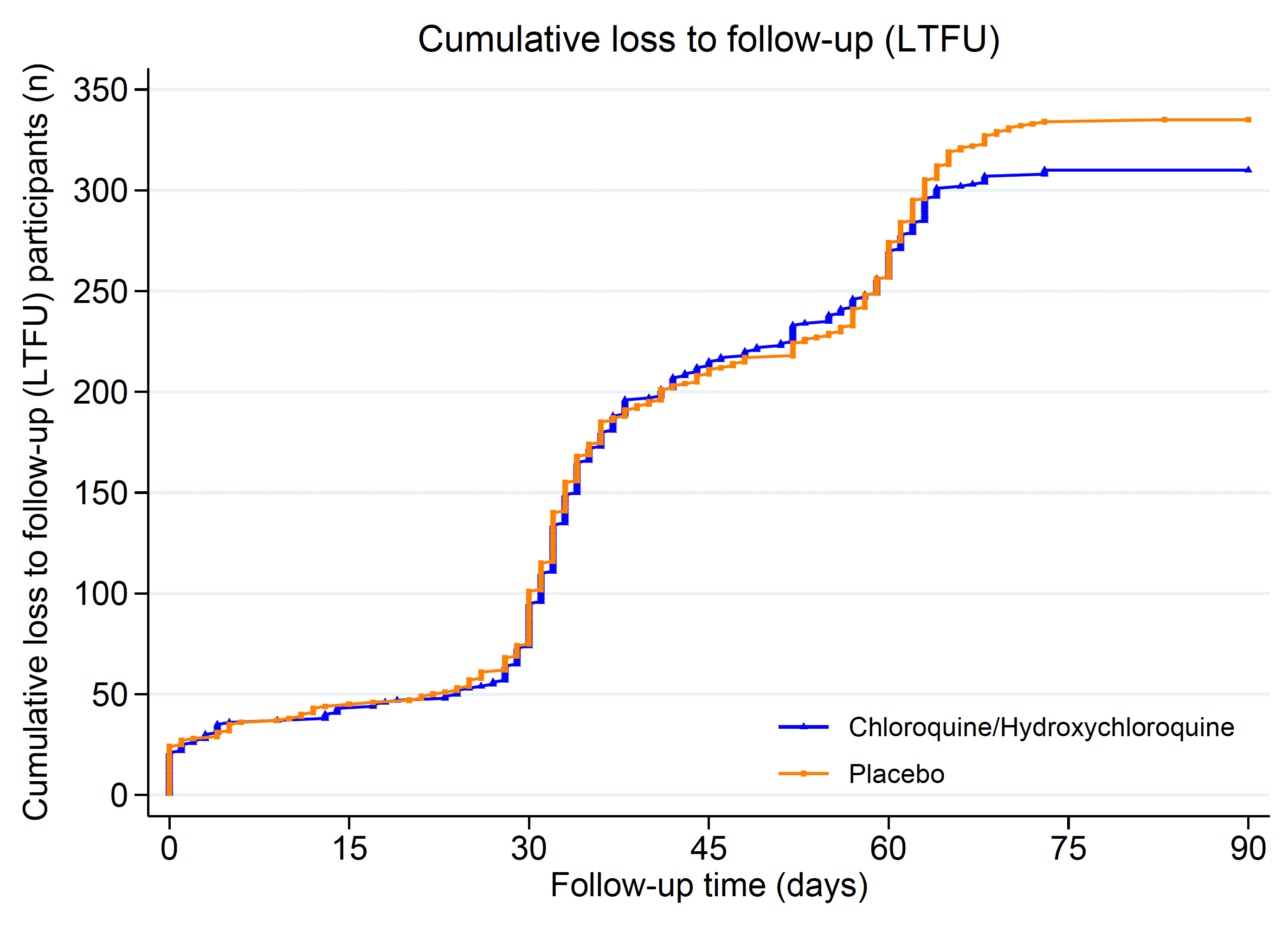


Day 90 corresponds to the end of study- 90 days (-3/ +7 days)

# Supplementary tables

## Table A1. - List of study sites

Benin

1. Centre Hospitalier Universitaire de Zone Abomey-Calavi, Abomey-Calavi
2. Hospital De Zone Allada, Allada

Côte d’Ivoire

1. University Hospital Center of Angré, Angré
2. University Hospital Center of Bouaké, Bouaké

Indonesia

1. Murni Teguh Memorial Hospital, Medan, North Sumatra
2. Bunda Thamrin Hospital, Medan, North Sumatra
3. Husada Utama Hospital, Surabaya, East Java
4. Airlangga University Hospital (UNAIR), Surabaya, East Java
5. Sardjito Hospital, Yogyakarta

Kenya

1. Mbagathi County Hospital, Nairobi
2. Fountain Healthcare Hospital, Eldoret

Mali

1. Hospital of Mali, Bamako
2. The Bamako Hospital of Dermatology, Bamako

Nepal

1. B.P. Koirala Institute of Health Sciences (BPKIHS), Dharan

Niger

1. Niamey (Epicentre France), Maradi

Pakistan

1. The Aga Khan University Hospital, Karachi

Thailand

1. Faculty of Tropical Medicine, Mahidol University, Bangkok

U.K.

1. Brighton and Sussex University Hospitals NHS Trust, Brighton
2. Imperial College Healthcare NHS Trust, London
3. Oxford University Hospital NHS Foundation Trust, Oxford
4. University Hospitals Coventry and Warwickshire NHS Trust, Coventry
5. Birmingham and Solihull Mental Health NHS Foundation Trust, Birmingham
6. The Dudley Group NHS Foundation Trust, Dudley
7. Rotherham, Doncaster and South Humber NHS Foudation Trust, Doncaster
8. University Hospitals of Morecambe Bay NHS Foundation Trust, Cumbria

Zambia

1. Zambart, Lusaka

## Table A2. – Baseline characteristics in the COPCOV trial (Per Protocol Analysis)

|  | **Chloroquine/Hydroxychloroquine** | **Placebo** | **Total** |
| --- | --- | --- | --- |
|  | **N=1,645** | **N=1,678** | **N=3,323** |
| Age years, median (IQR) | 30 (24‒39) | 30 (24‒40) | 30 (24‒40) |
| Sex, n (%) |  |  |  |
| Male | 846 (51.4) | 859 (51.2) | 1,705 (51.3) |
| Female | 798 (48.5) | 818 (48.7) | 1,616 (48.6) |
| Not specified | 1 (0.1) | 1 (0.1) | 2 (0.1) |
| Oral Temperature ⁰C, mean (SD) | 36.4 (0.5) | 36.4 (0.5) | 36.4 (0.5) |
|  |  |  |  |
| Weight (kg), mean (SD) | 65.1 (14.0) | 65.9 (14.8) | 65.5 (14.4) |
| Height (cm), mean (SD) | 165 (9) | 164 (10) | 164 (9) |
| BMI (kg/m^2^), median (IQR) | 23.2 (20.4‒26.9) | 23.4 (20.6‒27.2) | 23.3 (20.5‒27.1) |
| Smoking, n (%) |  |  |  |
| Yes | 203 (12.34) | 215 (12.81) | 418 (12.58) |
| Never smoked | 1,340 (81.46) | 1,349 (80.39) | 2,689 (80.92) |
| Former smoker | 102 (6.20) | 114 (6.79) | 216 (6.50) |
| COVID-19 in Household, n/N (%) | 240/1,644 (14.6) | 250/1,674 (14.9) | 490/3,318 (14.8) |
| **Existing comorbidities** |  |  |  |
| Chronic pulmonary disease (not asthma), n/N (%) | 3 (0.2) | 0 (0) | 3 (0.1) |
| Asthma (physician diagnosed), n/N (%) | 14 (0.9) | 13 (0.8) | 27 (0.8) |
| Chronic kidney disease, n/N (%) | 0/1,644 (0) | 0/1,677 (0) | 0/3,321 (0) |
| Liver disease, n/N (%) | 0/1,644 (0) | 0/1,677 (0) | 0/3,321 (0) |
| AIDS/HIV, n/N (%) | 32/1,644 (1.9) | 30/1,677 (1.8) | 62/3,321 (1.9) |
| Diabetes, n/N (%) | 14/1,643 (0.9) | 13/1,677 (0.8) | 27/3,320 (0.8) |
| Hypertension, n/N (%) | 27/1,644 (1.6) | 27/1,676 (1.6) | 54/3,320 (1.6) |
| Cancer, n/N (%) | 1/1,643 (0.06) | 1/1,676 (0.06) | 2/3,319 (0.06) |
| Condition requiring immunosuppressive drugs, n/N (%) | 1/1,644 (0.06) | 0/1,677 (0) | 1/3,321 (0.03) |
| Ischaemic heart disease, n/N (%) | 2/1,642 (0.1) | 0/1,675 (0) | 2/3,317 (0.06) |
| High cholesterol, n/N (%) | 4/1,639 (0.2) | 7/1,673 (0.4) | 11/3,312 (0.3) |
| Any chronic condition, n (%) | 84 (5.1) | 82 (4.9) | 166 (5.0) |
| **Baseline symptoms** |  |  |  |
| Fever, n (%) | 0 (0) | 0 (0) | 0 (0) |
| Cough, n (%) | 4 (0.2) | 2 (0.1) | 6 (0.2) |
| Sore throat, n (%) | 0 (0) | 1 (0.06) | 1 (0.03) |
| Runny nose (rhinorrhoea), n (%) | 0 (0) | 1 (0.06) | 1 (0.03) |
| Wheezing, n (%) | 1 (0.06) | 0 (0) | 1 (0.03) |
| Anosmia, n (%) | 0 (0) | 0 (0) | 0 (0) |
| Chest pain, n (%) | 0 (0) | 1 (0.06) | 1 (0.03) |
| Muscle pain (myalgia), n (%) | 1 (0.06) | 2 (0.1) | 3 (0.09) |
| Joint pain (arthralgia), n (%) | 2 (0.1) | 2 (0.1) | 4 (0.1) |
| Shortness of breath on exertion, n (%) | 0 (0) | 2 (0.1) | 2 (0.06) |
| Shortness of breath at rest, n (%) | 0 (0) | 0 (0) | 0 (0) |
| Fatigue/malaise, n (%) | 0 (0) | 2 (0.1) | 2 (0.06) |
| Itching, n (%) | 0 (0) | 1 (0.06) | 1 (0.03) |
| Headache, n (%) | 1 (0.06) | 3 (0.2) | 4 (0.1) |
| Dizziness, n (%) | 0 (0) | 3 (0.2) | 3 (0.09) |
| Visual disturbance, n (%) | 0 (0) | 1 (0.06) | 1 (0.03) |
| Abdominal pain, n (%) | 0 (0) | 2 (0.12) | 2 (0.06) |
| Poor appetite, n (%) | 1 (0.06) | 1 (0.06) | 2 (0.06) |
| Nausea, n (%) | 0 (0) | 2 (0.1) | 2 (0.06) |
| Vomiting, n (%) | 0 (0) | 1 (0.06) | 1 (0.03) |
| Diarrhoea, n (%) | 0 (0) | 0 (0) | 0 (0) |
| Skin rash, n (%) | 0 (0) | 2 (0.1) | 2 (0.06) |

## Table A3. – Outcomes of Chloroquine/Hydroxychloroquine and Placebo Pre-exposure Prophylaxis against COVID-19 in the COPCOV study (Per Protocol Analysis)

| **Outcome** | **Chloroquine/**  **Hydroxychloroquine**  **(N=1,645)** | **Placebo**  **(N=1,678)** | **Risk ratio (95%CI)** | **Fisher’s exact P-value** |
| --- | --- | --- | --- | --- |
| Total participant days | 150,505 | 153,442 |  |  |
| Symptomatic COVID-19. n(%); 95%CI | 222/1,645  13.5(11.9 to 15.2) | 263/1,678  15.7(14.0 to17.5) | 0.86 (0.73 to 1.02) | 0.077 |
| PCR-confirmed diagnosis. n/N (%); 95%CI | 22/1,645  1.3(0.8 to 2.0) | 50/1,678  3.0(2.2 to 3.9) | 0.45 (0.27 to 0.74) | 0.001 |
| Serology confirmed diagnosis (serum).  n (%); 95%CI | 207/1,431  14.5(12.7 to 16.4) | 242/1,468  16.5(14.6 to 18.5) | 0.88 (0.74 to 1.04) | 0.137 |
| Serology confirmed diagnosis (DBS).  n (%); 95%CI | 11/62  17.7(9.2 to 29.5) | 11/60  18.3(9.5 to 30.4) | 0.97 (0.45 to 2.06) | 1.000 |
| Asymptomatic COVID-19.  n (%); 95%CI | 247/1,645  15.0(13.3 to 16.8) | 245/1,678  14.6(12.9 to 16.4) | 1.03 (0.87, 1.21) | 0.769 |
| All SARS-CoV-2 infection  n(%); 95%CI | 469/1,645  28.5(26.3 to 30.8) | 508/1,678  30.3(28.1 to 32.5) | 0.94 (0.85 to 1.05) | 0.270 |
| All-cause respiratory illness. n(%); 95%CI | 41/1,645  2.5(1.8 to 3.4) | 66/1,678  3.9 (3.1 to 5.0) | 0.63 (0.43 to 0.93) | 0.023 |

## Table A4. – Summary of Serious Adverse Events in the COPCOV study

| **SAE ID** | **Treatment** | **Diagnosis** | **Relationship to study drug** |
| --- | --- | --- | --- |
| 1 | Placebo | Bronchitis | Not related |
| 2 | CQ/HCQ | Pneumonia | Not related |
| 3 | Placebo | Acute heart failure and pneumonia | Not related |
| 4 | CQ/HCQ | Dengue and pneumonia | Not related |
| 5 | Placebo | Gastroenteritis | Unlikely |
| 6 | Placebo | Pneumonia | Not related |
| 7 | CQ/HCQ | COVID-19 | Not related |
| 8 | Placebo | Malaria | Not related |
| 9 | Placebo | Extradural haematoma | Not related |
| 10 | Placebo | Nephrolithiasis | Not related |
| 11 | CQ/HCQ | Ovarian abscess | Not related |
| 12 | CQ/HCQ | Incomplete abortion | Unlikely |
| 13 | Placebo | Benign prostatic hyperplasia | Not related |
| 14 | CQ/HCQ | Ovarian cyst | Not related |
| *15 | CQ/HCQ | Constipation | Not related |
| *16 | CQ/HCQ | Gastric balloon complication | Unlikely |
| 17 | CQ/HCQ | Ovarian cyst | Not related |
| 18 | CQ/HCQ | Substance use disorder | Unlikely |

All Serious Adverse Events (SAEs) qualified as SAEs because the participant was hospitalised

CQ/HCQ = Chloroquine/hydroxychloroquine

* This individual had two separate SAEs during the study period

## Table A5. – Primary and secondary outcomes of Chloroquine/Hydroxychloroquine Therapy for Pre-exposure Prophylaxis against Covid-19 (missing outcomes treated as not having had covid-19 during the study period) ITT – Results presented as “Risk differences”

| **Outcome** | **Chloroquine/ Hydroxychloroquine (N= 2,320)** | **Placebo (N= 2,332)** | **Risk difference (95%CI)** | **Fisher's exact**  **P-value** |
| --- | --- | --- | --- | --- |
| Symptomatic COVID-19. n(%); 95%CI | 240/2320  10.3 (9.1 to 11.7) | 284/2332  12.2 (10.9 to 13.6) | -1.83 (-3.65 to -0.02) | 0.051 |
| PCR-confirmed diagnosis. n/N (%); 95%CI | 24/2320  1.0 (0.7 to 1.5) | 56/2332  2.4 (1.8 to 3.1) | -1.37 (-2.11 to -0.62) | <0.001 |
| Serology confirmed diagnosis (serum). n (%); 95%CI | 211/1462  14.4 (12.7 to 16.3) | 245/1498  16.4 (14.5 to 18.3) | -1.92 (-4.52 to 0.68) | 0.154 |
| Serology confirmed diagnosis (DBS). n (%); 95%CI | 25/280  8.9 (5.9 to 12.9) | 26/297  8.8 (5.8 to 12.6) | 0.17 (-4.46 to 4.81) | 1.000 |
| Asymptomatic SARS-CoV-2 infection. n (%); 95%CI | 267/2320  11.5 (10.2 to 12.9) | 280/2332  12.0 (10.7 to 13.4) | -0.50 (-2.35 to 1.35) | 0.617 |
| All SARS-CoV-2 infection n (%); 95%CI | 507/2320  21.9 (20.2 to 23.6) | 564/2332  24.2 (22.5 to 26.0) | -2.33 (-4.75 to 0.09) | 0.060 |
| All-cause respiratory illness*. N (%); 95%CI | 44/2320  1.9 (1.4 to 2.5) | 73/2332  3.1 (2.5 to 3.9) | -1.23 (-2.13 to -0.34) | 0.009 |
| Severity score  (Median (IQR) | 20.0  (5-85) | 21.5  (5-89) | NA | 1.000 |
| Participant reported work days lost | 700/181,263 | 932/184,688 | NA | 0.0002** |

**Assessed by a zero-inflated Poisson regression model.

## Table A6. – Outcomes of Chloroquine/Hydroxychloroquine and Placebo Pre-exposure Prophylaxis against COVID-19 in the COPCOV study (removing cases for which the SEAC judged that a study endpoint could not be determined)

| **Outcome** | **Chloroquine/**  **Hydroxychloroquine**  **(N=1,742)** | **Placebo**  **(N= 1,796)** | **Risk ratio (95%CI)** | **Fisher’s exact P-value** |
| --- | --- | --- | --- | --- |
| Symptomatic COVID-19. n (%); 95%CI | 240/1,742  13.8(12.2 to 15.5) | 284/1,796  15.8(14.2 to 17.6) | 0.87 (0.74 to 1.02) | 0.089 |
| PCR - confirmed diagnosis. n/N (%); 95%CI | 24/2,320  1.0(0.7 to 1.5) | 56/2,332  2.4(1.8 to 3.1) | 0.43 (0.27 to 0.69) | <0.001 |
| Serology confirmed diagnosis (serum).  n (%); 95%CI | 211/1,462  14.4(12.7 to 16.3) | 245/1,498  16.4(14.5 to 18.3) | 0.88 (0.74 to 1.05) | 0.154 |
| Serology confirmed diagnosis (DBS).  n (%); 95%CI | 25/280  8.9(5.9-12.9) | 26/297  8.8(5.8 to 12.6) | 1.02 (0.60 to 1.72) | 1.000 |
| Asymptomatic COVID-19.  n (%); 95%CI | 267/1,742  15.3(13.7 to 17) | 280/1,796  15.6(13.9 to 17.4) | 0.98 (0.84 to 1.15) | 0.852 |
| All SARS-CoV-2 infection  n (%); 95%CI | 507/1,742  29.1(27.0 to 31.3) | 564/1,796  31.4(29.3 to 33.6) | 0.93 (0.84 to 1.02) | 0.143 |
| All-cause respiratory illness. n (%); 95%CI | 44/2,320  1.9(1.4 to 2.5) | 73/2,332  3.1(2.5 to 3.9) | 0.61 (0.42 to 0.88) | 0.009 |

SEAC: Serology End-point Assessment Committee

## Table A7. – Summary characteristics of previously published pre-exposure prophylaxis studies considered for meta-analysis ^7-18^

| **First Author** | **Country** | **Publication date** | **Journal** | **Population** | **Sample size (HCQ)** | **Dose HCQ** | **Sample size (placebo/control)** | **Duration** | **Type of study** | **Primary outcome of study** |
| --- | --- | --- | --- | --- | --- | --- | --- | --- | --- | --- |
| **Abella** | USA | Sep-21 | JAMA Internal Medicine | HCWs | 66 | 600mg OD | 66 | 8 weeks | RCT, Placebo, Double blind | PCR confirmed SARS-CoV-2 (regardless of symptoms) |
| **Grau-Pujol** | Spain | Nov-21 | Trials | HCWs | 142 | 400mg OD for 4 days, then 400mg OW | 127 | 30 days | RCT, Placebo, Double blind | Lab confirmed symptomatic COVID-19 (PCR or seroconversion) |
| **McKinnon** | USA | Dec-21 | IJID | HCWs, first responders and those at occupationally high-risk | 1) 188 2) 199 | 1) 400mg once then 200mg OD 2) 400mg OW | 191 | 8 weeks | RCT, Placebo, Double blind | COVID-19 symptoms and lab confirmed (PCR or serology) |
| **Naggie** | USA | Aug-21 | MedRxiv/ later IJID | HCWs | 683 | 600mg x 2 D1, 400mg OD | 676 | 29 days | RCT, Placebo, Double blind | Symptomatic COVID-19 (PCR confirmed or not) |
| **Polo** | Spain, Venezuela, Ecuador | Mar-22 | Clinical Microbiology and Infection | HCWs | 231(+TDF/FTC placebo) + 220(+TDF/FTC active) | 200mg OD | 233(+TDF/FTC active) + 223(+TDF/FTC placebo) | 12 weeks | RCT, Placebo, Double blind; factorially designed with Tenofivir disoproxil fumarate/ Emtricitabine | PCR confirmed symptomatic COVID-19 |
| **Rajasingham** | USA and Canada | Jun-21 | CID | HCWs (79% perform aerosolising procedures) | 1) 494 2) 495 | 1) 400mg loading dose x2 then 400mg OW 2)400mg loading dose x2 then 400mg BW | 494 | 12 weeks | RCT, Placebo, Double blind | PCR confirmed COVID-19 or probable compatible illness |
| **Rojas-Serrano** | Mexico | Feb-22 | PLoS ONE | HCWs caring for COVID patients | 62 | 200mg OD | 65 | 60 days | RCT, Placebo, Double blind | PCR confirmed symptomatic COVID-19 |
| **Seet** | Singapore | Apr-21 | IJID | Quarantine | 432 | 400mg loading dose then 200mg OD | 619 (ascorbic acid) | 6 weeks | Cluster RCT Open label | PCR confirmed SARS-CoV-2 (regardless of symptoms) |
| **Syed** | Pakistan | Dec-21 | Cureus | HCWs (high risk) | 1) 48 2) 51 3) 55 | 1) 400mg loading dose x2 then 400mg OW 2) 400mg once every 3 weeks 3) 200mg once every 3 weeks | 46 | 12 weeks | RCT, Placebo but 1x placebo for 3 obviously different interventions | COVID-19 free survivals |
| **Vijayaraghavan** | India | May-21 | BMJ Open | Healthcare workers in an environment with exposure to COVID- 19 (all used PPE) | 213 | 800 mg loading dose, 400 mg once a week for 12 weeks | 203 | 12 weeks | RCT; Open-label | Laboratory-confirmed COVID- 19 infection within 6 months after randomisation |
| **Llanos-Cuentas** | Peru | Feb-23 | BMC Res notes | HCWs | 36 | 600mg D0, and 400mg every 2nd day | 32 | Up to 4 weeks | RCT, Open-label | PCR or rapid serology up to 4 weeks after randomisation |
| **Nasri** | Iran | Jan-23 | Adv Biomed Res | HCWs | 70 | 400mg OW | 73 | 12 weeks | RCT, Open-label | Not clear- symptoms and then PCR confirmation? |

HCWs= Healthcare workers, OD= Once Daily, OW= Once Weekly, BW= Twice Weekly, TDF/FTC= Tenofivir disoproxil fumarate/ Emtricitabine, RCT= Randomised Controlled Trial, PCR= Polymerase Chain Reaction, IJID= International Journal of Infectious Diseases, CID= Clinical Infectious Diseases.

## Table A8. – Listing of causes of PCR-confirmed respiratory illness

| **Trial** | **Anonymous ID** | **Treatment** | **PCR-confirmed results** |
| --- | --- | --- | --- |
| COPCOV | 1 | Chloroquine/Hydroxychloroquine | Rhinovirus/enterovirus* |
| COPCOV | 2 | Chloroquine/Hydroxychloroquine | Rhinovirus/enterovirus* |
| COPCOV | 3 | Chloroquine/Hydroxychloroquine | Rhinovirus/enterovirus* |
| COPCOV | 4 | Placebo | Human Coronavirus OC43 |
| COPCOV | 5 | Chloroquine/Hydroxychloroquine | Rhinovirus |
| COPCOV | 6 | Chloroquine/Hydroxychloroquine | Human Coronavirus NL63 |
| COPCOV | 7 | Chloroquine/Hydroxychloroquine | Adenovirus |
| COPCOV | 8 | Chloroquine/Hydroxychloroquine | Rhinovirus/enterovirus* |
| COPCOV | 9 | Placebo | Rhinovirus/enterovirus* |
| COPCOV | 10 | Placebo | Rhinovirus/enterovirus* |
| COPCOV | 11 | Chloroquine/Hydroxychloroquine | Influenza A Virus |
| COPCOV | 12 | Placebo | Metapneumovirus |
| COPCOV | 13 | Placebo | Influenza B Virus |
| COPCOV | 14 | Placebo | Rhinovirus/enterovirus* |
| COPCOV | 15 | Chloroquine/Hydroxychloroquine | Influenza A Virus |
| COPCOV | 16 | Placebo | Influenza B Virus |
| COPCOV | 17 | Placebo | Adenovirus |
| COPCOV | 18 | Placebo | Adenovirus |
| COPCOV | 19 | Placebo | Rhinovirus/enterovirus* |
| COPCOV | 20 | Placebo | Rhinovirus/enterovirus* |
| COPCOV | 21 | Chloroquine/Hydroxychloroquine | Influenza A Virus |
| COPCOV | 22 | Chloroquine/Hydroxychloroquine | Rhinovirus/enterovirus* |
| COPCOV | 23 | Chloroquine/Hydroxychloroquine | Rhinovirus/enterovirus* |
| COPCOV | 24 | Placebo | Rhinovirus/enterovirus* |
| COPCOV | 25 | Chloroquine/Hydroxychloroquine | Rhinovirus/enterovirus* |
| COPCOV | 26 | Chloroquine/Hydroxychloroquine | Respiratory Syncytial Virus, Influenza A Virus |
| COPCOV | 27 | Chloroquine/Hydroxychloroquine | Rhinovirus/enterovirus* |
| COPCOV | 28 | Chloroquine/Hydroxychloroquine | Influenza B Virus |
| COPCOV | 29 | Chloroquine/Hydroxychloroquine | Rhinovirus/enterovirus* |
| COPCOV | 30 | Placebo | Rhinovirus/enterovirus* |
| COPCOV | 31 | Placebo | Metapneumovirus |
| COPCOV | 32 | Chloroquine/Hydroxychloroquine | Parainfluenza Virus 2 |
| COPCOV | 33 | Chloroquine/Hydroxychloroquine | Respiratory Syncytial Virus, Rhinovirus/enterovirus* |
| COPCOV | 34 | Placebo | Rhinovirus/enterovirus* |
| COPCOV | 35 | Placebo | Adenovirus |
| COPCOV | 36 | Placebo | Rhinovirus/enterovirus* |
| COPCOV | 37 | Chloroquine/Hydroxychloroquine | Rhinovirus/enterovirus* |
| COPCOV | 38 | Chloroquine/Hydroxychloroquine | SARS-COV-2 |
| COPCOV | 39 | Chloroquine/Hydroxychloroquine | SARS-COV-2 |
| COPCOV | 40 | Chloroquine/Hydroxychloroquine | SARS-COV-2 |
| COPCOV | 41 | Placebo | SARS-COV-2 |
| COPCOV | 42 | Placebo | SARS-COV-2 |
| COPCOV | 43 | Placebo | SARS-COV-2 |
| COPCOV | 44 | Placebo | SARS-COV-2 |
| COPCOV | 45 | Chloroquine/Hydroxychloroquine | SARS-COV-2 |
| COPCOV | 46 | Placebo | SARS-COV-2 |
| COPCOV | 47 | Chloroquine/Hydroxychloroquine | SARS-COV-2 |
| COPCOV | 48 | Placebo | SARS-COV-2 |
| COPCOV | 49 | Placebo | SARS-COV-2 |
| COPCOV | 50 | Placebo | SARS-COV-2 |
| COPCOV | 51 | Placebo | SARS-COV-2 |
| COPCOV | 52 | Placebo | SARS-COV-2 |
| COPCOV | 53 | Chloroquine/Hydroxychloroquine | SARS-COV-2 |
| COPCOV | 54 | Chloroquine/Hydroxychloroquine | SARS-COV-2 |
| COPCOV | 55 | Placebo | SARS-COV-2 |
| COPCOV | 56 | Chloroquine/Hydroxychloroquine | SARS-COV-2 |
| COPCOV | 57 | Placebo | SARS-COV-2 |
| COPCOV | 58 | Placebo | SARS-COV-2, Adenovirus |
| COPCOV | 59 | Placebo | SARS-COV-2 |
| COPCOV | 60 | Placebo | SARS-COV-2 |
| COPCOV | 61 | Placebo | SARS-COV-2 |
| COPCOV | 62 | Placebo | SARS-COV-2 |
| COPCOV | 63 | Placebo | SARS-COV-2 |
| COPCOV | 64 | Placebo | SARS-COV-2 |
| COPCOV | 65 | Placebo | SARS-COV-2 |
| COPCOV | 66 | Placebo | SARS-COV-2 |
| COPCOV | 67 | Placebo | SARS-COV-2 |
| COPCOV | 68 | Placebo | SARS-COV-2 |
| COPCOV | 69 | Placebo | SARS-COV-2, Rhinovirus |
| COPCOV | 70 | Placebo | SARS-COV-2 |
| COPCOV | 71 | Placebo | SARS-COV-2 |
| COPCOV | 72 | Placebo | SARS-COV-2 |
| COPCOV | 73 | Placebo | SARS-COV-2 |
| COPCOV | 74 | Placebo | SARS-COV-2 |
| COPCOV | 75 | Chloroquine/Hydroxychloroquine | SARS-COV-2 |
| COPCOV | 76 | Placebo | SARS-COV-2 |
| COPCOV | 77 | Chloroquine/Hydroxychloroquine | SARS-COV-2 |
| COPCOV | 78 | Placebo | SARS-COV-2 |
| COPCOV | 79 | Placebo | SARS-COV-2 |
| COPCOV | 80 | Placebo | SARS-COV-2 |
| COPCOV | 81 | Placebo | SARS-COV-2, Rhinovirus |
| COPCOV | 82 | Placebo | SARS-COV-2 |
| COPCOV | 83 | Placebo | SARS-COV-2 |
| COPCOV | 84 | Chloroquine/Hydroxychloroquine | SARS-COV-2 |
| COPCOV | 85 | Placebo | SARS-COV-2 |
| COPCOV | 86 | Chloroquine/Hydroxychloroquine | SARS-COV-2 |
| COPCOV | 87 | Chloroquine/Hydroxychloroquine | SARS-COV-2 |
| COPCOV | 88 | Chloroquine/Hydroxychloroquine | SARS-COV-2 |
| COPCOV | 89 | Placebo | SARS-COV-2 |
| COPCOV | 90 | Chloroquine/Hydroxychloroquine | SARS-COV-2 |
| COPCOV | 91 | Placebo | SARS-COV-2 |
| COPCOV | 92 | Chloroquine/Hydroxychloroquine | SARS-COV-2 |
| COPCOV | 93 | Placebo | SARS-COV-2 |
| COPCOV | 94 | Placebo | SARS-COV-2 |
| COPCOV | 95 | Placebo | SARS-COV-2 |
| COPCOV | 96 | Chloroquine/Hydroxychloroquine | SARS-COV-2 |
| COPCOV | 97 | Chloroquine/Hydroxychloroquine | SARS-COV-2 |
| COPCOV | 98 | Placebo | SARS-COV-2 |
| COPCOV | 99 | Chloroquine/Hydroxychloroquine | SARS-COV-2 |
| COPCOV | 100 | Placebo | SARS-COV-2 |
| COPCOV | 101 | Chloroquine/Hydroxychloroquine | SARS-COV-2 |
| COPCOV | 102 | Placebo | SARS-COV-2 |
| COPCOV | 103 | Placebo | SARS-COV-2 |
| COPCOV | 104 | Placebo | SARS-COV-2 |
| COPCOV | 105 | Chloroquine/Hydroxychloroquine | SARS-COV-2 |
| COPCOV | 106 | Placebo | SARS-COV-2 |
| COPCOV | 107 | Placebo | SARS-COV-2 |
| COPCOV | 108 | Chloroquine/Hydroxychloroquine | SARS-COV-2 |
| COPCOV | 109 | Placebo | SARS-COV-2 |
| COPCOV | 110 | Placebo | SARS-COV-2 |
| COPCOV | 111 | Placebo | SARS-COV-2 |
| COPCOV | 112 | Placebo | SARS-COV-2 |
| COPCOV | 113 | Placebo | SARS-COV-2 |
| COPCOV | 114 | Chloroquine/Hydroxychloroquine | SARS-COV-2 |
| COPCOV | 115 | Placebo | SARS-COV-2 |
| COPCOV | 116 | Chloroquine/Hydroxychloroquine | SARS-COV-2 |
| COPCOV | 117 | Placebo | SARS-COV-2 |

*One of the two respiratory virus panel assays used was unable to distinguish between rhinovirus and enterovirus, hence the determination of Rhinovirus/enterovirus.

# References for COPCOV study supplementary materials

1. Corman VM, Landt O, Kaiser M, Molenkamp R, Meijer A, Chu DK et al. Detection of 2019 novel coronavirus (2019-nCoV) by real-time RT-PCR. Euro Surveill. 2020 Jan;25(3):2000045. doi: 10.2807/1560-7917.ES.2020.25.3.2000045. Erratum in: Euro Surveill. 2020 Apr;25(14): Erratum in: Euro Surveill. 2020 Jul;25(30): Erratum in: Euro Surveill. 2021 Feb;26(5): PMID: 31992387; PMCID: PMC6988269.
2. Shu B, Kirby MK, Davis WG, Warnes C, Liddell J, Liu J et al. Multiplex Real-Time Reverse Transcription PCR for Influenza A Virus, Influenza B Virus, and Severe Acute Respiratory Syndrome Coronavirus 2. Emerg Infect Dis. 2021;27(7):1821-1830. doi: 10.3201/eid2707.210462. PMID: 34152951; PMCID: PMC8237866.
3. Stoddard RA, Gee JE, Wilkins PP, McCaustland K, Hoffmaster AR. Detection of pathogenic Leptospira spp. through TaqMan polymerase chain reaction targeting the LipL32 gene. Diagn Microbiol Infect Dis. 2009 Jul;64(3):247-55. doi: 10.1016/j.diagmicrobio.2009.03.014. Epub 2009 Apr 22. PMID: 19395218.
4. Zhang K, Misra A, Kim PJ, Moghadas SM, Langley JM, Smieja M. Rapid disappearance of influenza following the implementation of COVID-19 mitigation measures in Hamilton, Ontario. Can Commun Dis Rep. 2021 May 7;47(4):202-209. doi: 10.14745/ccdr.v47i04a04. PMID: 34035666; PMCID: PMC8127684.
5. Garcia-Albeniz X, Del Amo J, Polo R, Morales-Asencio JM, Hernán MA. Systematic review and meta-analysis of randomized trials of hydroxychloroquine for the prevention of COVID-19. Eur J Epidemiol 2022;37:789-96.
6. Sterne JAC, Savović J, Page MJ, Elbers RG, Blencowe NS, Boutron I et al. RoB 2: a revised tool for assessing risk of bias in randomised trials. BMJ. 2019 Aug 28;366:l4898. doi: 10.1136/bmj.l4898. PMID: 31462531.
7. Syed F, Hassan M, Arif MA, Batool S, Niazi R, Laila UE, et al. Pre-exposure Prophylaxis With Various Doses of Hydroxychloroquine Among Healthcare Personnel With High-Risk Exposure to COVID-19: A Randomized Controlled Trial. Cureus 2021;13:e20572.
8. Abella BS, Jolkovsky EL, Biney BT, Uspal JE, Hyman MC, Frank I, et al. Efficacy and Safety of Hydroxychloroquine vs Placebo for Pre-exposure SARS-CoV-2 Prophylaxis Among Health Care Workers: A Randomized Clinical Trial. JAMA Intern Med 2021;181:195-202.
9. Rajasingham R, Bangdiwala AS, Nicol MR, Skipper CP, Pastick KA, Axelrod ML, et al. Hydroxychloroquine as Pre-exposure Prophylaxis for Coronavirus Disease 2019 (COVID-19) in Healthcare Workers: A Randomized Trial. Clin Infect Dis 2021;72:e835-e43.
10. Rojas-Serrano J, Portillo-Vasquez AM, Thirion-Romero I, Vázquez-Pérez J, Mejía-Nepomuceno F, Ramírez-Venegas A, et al. Hydroxychloroquine for prophylaxis of COVID-19 in health workers: A randomized clinical trial. PLoS ONE 2022;17:e0261980.
11. Polo R, Garcia-Albeniz X, Teran C, Morales M, Rial-Crestelo D, Garcinuño MA, et al. Daily tenofovir disoproxil fumarate/emtricitabine and hydroxychloroquine for pre-exposure prophylaxis of COVID-19: a double-blind placebo-controlled randomized trial in healthcare workers. Clin Microbiol Infect 2023 Jan;29(1):85-93. doi: 10.1016/j.cmi.2022.07.006. Epub 2022 Aug 5. PMID: 35940567; PMCID: PMC9352647.
12. Tirupakuzhi Vijayaraghavan BK, Jha V, Rajbhandari D, Myatra SN, Ghosh A, Bhattacharya A, et al. Hydroxychloroquine plus personal protective equipment versus personal protective equipment alone for the prevention of laboratory-confirmed COVID-19 infections among healthcare workers: a multicentre, parallel-group randomised controlled trial from India. BMJ Open 2022 Jun 1;12(6):e059540. doi: 10.1136/bmjopen-2021-059540. PMID: 35649613; PMCID: PMC9160584.
13. Seet RCS, Quek AML, Ooi DSQ, Sengupta S, Lakshminarasappa SR, Koo CY, et al. Positive impact of oral hydroxychloroquine and povidone-iodine throat spray for COVID-19 prophylaxis: An open-label randomized trial. Int J Infect Dis 2021;106:314-22.
14. McKinnon JE, Wang DD, Zervos M, Saval M, Marshall-Nightengale L, Kilgore P, et al. Safety and tolerability of hydroxychloroquine in health care workers and first responders for the prevention of COVID-19: WHIP COVID-19 Study. Int J Infect Dis 2022;116:167-73.
15. Grau-Pujol B, Camprubi-Ferrer D, Marti-Soler H, Fernández-Pardos M, Carreras-Abad C, Andrés MV, et al. Pre-exposure prophylaxis with hydroxychloroquine for COVID-19: a double-blind, placebo-controlled randomized clinical trial. Trials 2021;22:808.
16. Naggie S, Milstone A, Castro M, Collins SP, Lakshmi S, Anderson DJ, et al. Hydroxychloroquine for pre-exposure prophylaxis of COVID-19 in health care workers: a randomized, multicenter, placebo-controlled trial Healthcare Worker Exposure Response and Outcomes of Hydroxychloroquine (HERO-HCQ). Int J Infect Dis. 2023 Apr;129:40-48. doi: 10.1016/j.ijid.2023.01.019. Epub 2023 Jan 20. PMID: 36682681; PMCID: PMC9851717.
17. Nasri E, Fakhim H, Salahi M, Ghafel S, Pourajam S, Darakhshandeh A, et al. Efficacy of Hydroxychloroquine in Pre-exposure Severe Acute Respiratory Syndrome Coronavirus 2 Prophylaxis among High-Risk HealthCare Workers: A Multicenter Study. Adv Biomed Res 2023; 12: 3.
18. Llanos-Cuentas A, Schwalb A, Quintana JL, Delfin B, Alvarez F, Ugarte-Gil C, et al. Hydroxychloroquine to prevent SARS-CoV-2 infection among healthcare workers: early termination of a phase 3, randomised, open-label, controlled clinical trial. BMC Res Notes 2023; 16(1): 22.

# List of legends for S1 Appendix

Fig A1. – Atlas showing those countries in which investigators were contacted to enquire whether they would be interested in, and able to join the COPCOV study

Fig A2. – Atlas showing the location of the COPCOV trial sites, the 4-aminoquinoline tested (chloroquine or hydroxychloroquine), and the approximate numbers recruited

Fig A3. – Graph showing cumulative enrollment over time (per week) by country

Fig A4. – Funnel plot showing the relationship between estimated risk ratio and its standard error for the 4-aminoquinoline COVID-19 pre-exposure chemoprevention RCTs included in primary endpoint efficacy meta-analysis

Fig A5. – meta-analysis of 4-aminoquinoline COVID-19 pre-exposure chemoprevention RCTs based on individual study primary end-points using Risk Of Bias tool (RoB 2)

Fig A6. – Meta-analysis of the 4-aminoquinoline COVID-19 pre-exposure chemoprevention RCTs for the endpoint of PCR-confirmed symptomatic COVID-19

Fig A7. – Meta-analysis of adverse events leading to treatment discontinuation reported in double-blind, placebo-controlled, 4-aminoquinoline COVID-19 pre-exposure chemoprevention RCTs

Fig A8. – Graph showing cumulative loss to follow-up (LTFU) for Hydroxychloroquine/ Chloroquine arms and Placebo arm

Table A1. - List of study sites

Table A2. – Baseline characteristics in the COPCOV trial (Per Protocol Analysis)

Table A3. – Outcomes of Chloroquine/Hydroxychloroquine and Placebo Pre-exposure Prophylaxis against COVID-19 in the COPCOV study (Per Protocol Analysis)

Table A4. – Summary of Serious Adverse Events in the COPCOV study

Table A5. – Primary and secondary outcomes of Chloroquine/Hydroxychloroquine Therapy for Pre-exposure Prophylaxis against Covid-19 (missing outcomes treated as not having had covid-19 during the study period) ITT – Results presented as “Risk differences”

Table A6. – Outcomes of Chloroquine/Hydroxychloroquine and Placebo Pre-exposure Prophylaxis against COVID-19 in the COPCOV study (removing cases for which the SEAC judged that a study endpoint could not be determined)

Table A7. – Summary characteristics of previously published pre-exposure prophylaxis studies considered for meta-analysis^7-18^

Table A8. – Listing of causes of PCR-confirmed respiratory illness
