# Supplementary material for: Evaluation of hydroxychloroquine or chloroquine for the prevention of COVID-19 (COPCOV): A double-blind, randomised, placebo-controlled trial
Source: PLoS Med. 2024 Sep 12;21(9):e1004428. doi: 10.1371/journal.pmed.1004428 (PMC11392261; doi:10.1371/journal.pmed.1004428)
Supplement: Attachment — Submitted filename: PLOS Med reviews_Final.docx [file pmed.1004428.s008.docx]

Dear Dr. Schilling,

Thank you very much for submitting your manuscript "Hydroxychloroquine or chloroquine prevention of COVID-19 (COPCOV) a double-blind, randomised, placebo-controlled trial" (PMEDICINE-D-23-02452R1) for consideration at PLOS Medicine. 

Your paper was discussed among the editors and sent to independent reviewers, including a statistical reviewer. The reviews are appended at the bottom of this email and any accompanying reviewer attachments can be seen via the link below:

In light of these reviews, we will not be able to accept the manuscript for publication in the journal in its current form, but we would like to invite you to submit a revised version that addresses the reviewers' and editors' comments fully. You will recognize that we cannot make a decision about publication until we have seen the revised manuscript and your response, and we expect to seek re-review by one or more of the reviewers. 

In revising the manuscript for further consideration, your revisions should address the specific points made by each reviewer and the editors. Please also check the guidelines for revised papers at [http://journals.plos.org/plosmedicine/s/revising-your-manuscript](https://protect-eu.mimecast.com/s/L8foCgxYvHR51JC68xdY?domain=journals.plos.org) for any that apply to your paper. In your rebuttal letter you should indicate your response to the reviewers' and editors' comments, the changes you have made in the manuscript, and include either an excerpt of the revised text or the location (eg: page and line number) where each change can be found. Please submit a clean version of the paper as the main article file; a version with changes marked should be uploaded as a marked up manuscript.

In addition, we request that you upload any figures associated with your paper as individual TIF or EPS files with 300dpi resolution at resubmission; please read our figure guidelines for more information on our requirements: [http://journals.plos.org/plosmedicine/s/figures](https://protect-eu.mimecast.com/s/8LcMCjvQyfX140sg-Z1J?domain=journals.plos.org). While revising your submission, please upload your figure files to the PACE digital diagnostic tool, [https://pacev2.apexcovantage.com/](https://protect-eu.mimecast.com/s/WrcMCkwQzCj3yvhGU4hg?domain=pacev2.apexcovantage.com). PACE helps ensure that figures meet PLOS requirements. To use PACE, you must first register as a user. Then, login and navigate to the UPLOAD tab, where you will find detailed instructions on how to use the tool. If you encounter any issues or have any questions when using PACE, please email us at [PLOSMedicine@plos.org](mailto:PLOSMedicine@plos.org).

We hope to receive your revised manuscript by Oct 30 2023 11:59PM. Please email us ([plosmedicine@plos.org](mailto:plosmedicine@plos.org)) if you have any questions or concerns.

***Please note while forming your response, if your article is accepted, you may have the opportunity to make the peer review history publicly available. The record will include editor decision letters (with reviews) and your responses to reviewer comments. If eligible, we will contact you to opt in or out.***

We ask every co-author listed on the manuscript to fill in a contributing author statement, making sure to declare all competing interests. If any of the co-authors have not filled in the statement, we will remind them to do so when the paper is revised. If all statements are not completed in a timely fashion this could hold up the re-review process. If new competing interests are declared later in the revision process, this may also hold up the submission. Should there be a problem getting one of your co-authors to fill in a statement we will be in contact. YOU MUST NOT ADD OR REMOVE AUTHORS UNLESS YOU HAVE ALERTED THE EDITOR HANDLING THE MANUSCRIPT TO THE CHANGE AND THEY SPECIFICALLY HAVE AGREED TO IT. You can see our competing interests policy here: [http://journals.plos.org/plosmedicine/s/competing-interests](https://protect-eu.mimecast.com/s/fLcUClvQAf8qwlUl8rsH?domain=journals.plos.org).

**RESPONSE:** Thank you. All Authors have submitted their conflict of interest statements.

Please use the following link to submit the revised manuscript: 

[https://www.editorialmanager.com/pmedicine/](https://protect-eu.mimecast.com/s/6SxhCm2RBf3LoqsMGn0U?domain=editorialmanager.com)

Your article can be found in the "Submissions Needing Revision" folder. 

To enhance the reproducibility of your results, we recommend that you deposit your laboratory protocols in protocols.io, where a protocol can be assigned its own identifier (DOI) such that it can be cited independently in the future. Additionally, PLOS ONE offers an option to publish peer-reviewed clinical study protocols. Read more information on sharing protocols at [https://plos.org/protocols?utm_medium=editorial-email&utm_source=authorletters&utm_campaign=protocols](https://protect-eu.mimecast.com/s/iwLyCnYQDC2pZVCMmewv?domain=plos.org)

Please ensure that the paper adheres to the PLOS Data Availability Policy (see [http://journals.plos.org/plosmedicine/s/data-availability](https://protect-eu.mimecast.com/s/KgzuCovREfRpVmCBqOMl?domain=journals.plos.org)), which requires that all data underlying the study's findings be provided in a repository or as Supporting Information. For data residing with a third party, authors are required to provide instructions with contact information for obtaining the data. PLOS journals do not allow statements supported by "data not shown" or "unpublished results." For such statements, authors must provide supporting data or cite public sources that include it. 

Please let me know if you have any questions, and we look forward to receiving your revised manuscript. 

Sincerely,

Richard Turner PhD
Consulting editor, PLOS Medicine

[plosmedicine@plos.org](mailto:plosmedicine@plos.org)

-----------------------------------------------------------

Requests from the editors:

In the text and supplementary material, several organizations are mentioned in the context of provision or packaging of study drugs (e.g., "Accord Healthcare" and "Piramal"). We feel that all such contributions should mentioned in the financial disclosures (submission form).

**RESPONSE:** Thank you. The aforementioned relationships were not financial as Accord donated the Investigational Medicinal Product. We will mention this.

We ask you to adapt the competing interest statement (submission form) to name relevant members of PLOS Medicine's editorial board among the authors.

**RESPONSE:** Thank you. The two authors have amended their ICMJE forms accordingly.

We suggest adapting the title as follows: "Evaluation of Hydroxychloroquine or chloroquine for prevention of COVID-19 (COPCOV): A double-blind, randomised, placebo-controlled trial

**RESPONSE:** Thank you we have amended the title and added a short title. Lines 1-4.

Please adapt the short title to contain brief details of the study. 

Please ensure that the revised abstract follows CONSORT; if secondary endpoint outcomes are to be reported, all should be included to avoid an appearance of cherry-picking.

**RESPONSE:** Thank you we have added the missing reported secondary outcomes. Lines 93-94.

Please combine the "Methods" and "Findings" subsections of the abstract.

**RESPONSE:** Thank you - we have done this.

Please add a new final sentence to the combined subsection, which should begin "Study imitations include ..." or similar and should quote 2-3 of the study's main limitations.

**RESPONSE:** We have added this. Lines 100-104.

Please quote aggregate demographic details for study participants in the abstract.

**RESPONSE:** These have now been added. Lines 88-89.

In place of the "Research in Context" section, please substitute a new "Author Summary" section in PLOS Medicine style. You may find it helpful to consult one or two recent research papers in the journal to get a sense of the preferred style.

**RESPONSE:** This has now been changed. Lines 115-142.

Noting comments from the referees on the statement "Published studies suggest clinical benefit", please adapt the abstract and main text to reflect the implications of the published individual studies.

**RESPONSE:** We have made the following change to the abstract in lines 79-80:

*Meta-analysis of published studies is in the direction of clinical benefit, but guidelines recommend against its use.*

We have reworked the introduction in response and we have added more detail in the response to reviewers.

The widely publicised controversies, and negative regulatory responses adversely affected recruitment and study conduct in chemoprevention clinical trials attempting to provide definitive evidence. Despite this some investigators did successfully complete their RCTs.^9-22^ Taken together these studies point towards moderate preventive efficacy,^23^ but the evidence is far from conclusive.

Please adapt the final sentence of the Introduction (main text; "We present the results ...") to state the study's aim.

**RESPONSE:** Thank you - we have made this change lines 166-167.

Please restructure the Discussion section (main text) so that the first paragraph summarizes the study's main findings, with these being discussed in subsequent paragraphs.

**RESPONSE:** This has now been done. Line 321 onwards.

Please remove all trademarks from the paper.

**RESPONSE:** Thank you - we have removed these.

Please remove the information on funding from the title page and from the end of the main text. In the event of publication, this information will appear in the article metadata, via entries in the submission form. 

In the reference list, please convert italics to plain text.

**RESPONSE:** All italics have now been converted to plain text.

Please add accessed dates to online references such as reference 1.

**RESPONSE:** We have added the last accessed dates.

Noting reference 2, please list 6 author names followed by "et al.", where appropriate.

**RESPONSE:** We have listed 6 authors.

Please use the journal name abbreviation "PLoS ONE".

**RESPONSE:** Now PLoS ONE.

Please adapt the title for Fig 1 to "Participant flowchart" or similar.

**RESPONSE:** Thank you we have made this change.

Please include a completed CONSORT checklist as a supplementary file, labelled "S1_CONSORT_Checklist" or similar and referred to as such in the Methods section (main text). 
**RESPONSE:** Thank you we have made this change.

In the checklist, please refer to individual items by section (e.g., "Methods") and paragraph number, not by line or page numbers as these generally change in the event of publication. 

Is there a formal study protocol that you can attach as "S2_Protocol"?

**RESPONSE:** Yes we have attached this as S3_Protocol

Please rename the attached statistical analysis plan "S3_Statistical Analysis Plan" or similar.

**RESPONSE:** This has now been renamed S4_Statistical Analysis Plan.

Comments from the reviewers:

*** Reviewer #1: 

Statistical review

This paper reports a RCT comparing pooled Hydroxychloroquine + chloroquine vs placebo for prevention of covid-19 infection. Although the sample size enrolled was much lower than target, the trial is still very large and provides useful evidence about the positive effect of the drugs. The trial was reported well and I had only some minor comments:

**RESPONSE:** Thank you very much for your positive comments.

1. Abstract "The primary endpoint was symptomatic COVID-19": perhaps add 'during the follow-up period.' to clarify that this wasn't time-to-event.

**RESPONSE:** We agree with the reviewer and have revised the sentence accordingly. Line 86.

2. Abstract: The secondary outcomes reported in the abstract do not align perfectly with the secondary outcomes in the clinicaltrials.gov registration page (or later in the paper). If those outcomes were collected it would be good to report them in the abstract.

**RESPONSE:** For those that we have we have analysed, but were not in abstract, they have now been included. Some of the secondary outcomes in the clinicaltrials.gov have not been reported in this manuscript. We have included only those secondary outcomes that are directly relevant to the primary outcome. Analysis of other secondary outcomes will go into separate papers so that detailed information about the secondary outcomes can be discussed in those papers.

3. Abstract: "Clinical trials need to be supported and protected, particularly in pandemic emergencies." - Although I see the rationale for this statement after reading the paper's discussion, I didn't see how this followed from the rest of the abstract so would leave it out.
**RESPONSE:** Thank you very much for this comment. We agree with the comment on continuity, but it is such an important point (exemplified by this study) and the second reviewer also felt strongly that this was an important message, so we have retained it.

4. Page 9: were any covariates adjusted for in the log-binomial models?
**RESPONSE:** We did not adjust for any baseline covariates. As shown in the baseline characteristics table, the covariates were evenly balanced between arms. There were no covariates that were highly predictive of the outcome, adjustment would not be expected to reduce the variance in the treatment effect estimate.

5. Page 9: was there non-negligible loss-to-follow-up (the sample size calculation planned for 20%) and if so how was this handled in the ITT analysis?

**RESPONSE:** The loss to follow up was 14% but the primary outcome data was also missing due to other reasons as outlined in the CONSORT chart (primarily because of missing serology data). In terms of the ITT analyses, missing outcomes in the primary end point ITT analysis were treated as not having had COVID-19 during the study period. We note that participants for whom seroconversion could not be assessed could still have in theory met the primary endpoint by having a positive PCR test.

6. Page 10 "In symptomatic patients there were no differences in symptom severity scores." - I would add no significant difference (and preferably would include the median score in each arm, similarly to other outcomes have summary by arm).

**RESPONSE:** Thank you for this comment. We have added “significant” and also included the summaries in the text, i.e. median etc. Lines 297-298

7. Page 10 "a mean difference of 104 days (95% CI, 12 to 199)." - is there a reason the authors did not provide a p-value for this outcome? Other secondary outcomes have them.

**RESPONSE:** The number of workdays lost was analysed under a Bayesian zero-inflated Poisson regression model, so there is a 95% credible interval but no corresponding p-value.

James Wason

*** Reviewer #2: 

In this double-blind, placebo controlled, multi-national, randomised trial the authors test whether a three month chemoprophylaxis with hydroxychloroquine or chloroquine can reduce the incidence of clinical COVID-19. Several secondary outcomes were also reported
The trial was planned for 40,000 participants, of whom 4,652 were recruited, after which point the trial had to close due to lack of recruitment. 
The hypothesis is clearly stated, and, despite the premature closure, I believe, within reasonable clinical certainty, can conclude on the hypothesis. 
The results seem clear, and the wording in the interpretation, are well-balanced by the authors. They also manage to balance the limitations. 
In all aspects, apart from the premature closure, this is a very high quality RCT. 
I strongly agree with the authors on their point that especially during crises like the COVID-19 pandemic, stakeholders should stand up for the completion of RCT´s since, at the end of the day, this design is the only that can inform us solidly, when conducted rigorously.

**RESPONSE:** We thank the reviewer for these positive comments.

I have few comments: 

The authors state:
"This allowed for approximately 20% loss to follow up, withdrawals, protocol deviations and non-adherence, and provided 80% power to detect a 23% reduction in symptomatic COVID-19 incidence (from an estimated 3%) for each drug individually with 95% confidence. There were protracted delays and difficulties with recruitment as the study was starting in 2020"
This seems extremely ambitious, however, sometime the "perfect becomes enemy of the good", insofar as the chance of completing extremely ambitious projects decreases with the increasing target sample size. I speak of bitter personal experience…. As far as I can see, the sample size calculation wanted to detect a difference between approx. 3% to approx. 2.4%. In light of the pandemic threat and the situation when the trial was planned, I am a little puzzled: would a protective effect as small as this actually have been clinically relevant in stopping the pandemic? Could the authors elaborate on this in the discussion

**RESPONSE:** Thank you for this important comment. At the study outset we had no idea how bad the pandemic would become, whether the trial could achieve its objectives, or what the minimum benefit that would change policy would be. A 20% reduction in risk could have been worthwhile for an intervention which is very safe (such as HCQ), and in particular when there were no viable alternatives (e.g. no vaccinations, no treatments or other chemoprophylaxis agents).

We have reordered the discussion as advised and acknowledge these uncertainties in the revised discussion.

In the discussion, first part: "Few drugs have excited such controversy as hydroxychloroquine in COVID-19. Bureaucracy, politicisation, unfounded opinions, false claims and outright fraud conspired against the conduct of the clinical trials needed to provide objective evidence early in the pandemic" 
I agree, this was outrageous. However, I feel, the results of the trial should be respected in the first lines of the discussion. This is an opinion, I´m aware, however, maybe the authors should "kill this darling", at least regarding the prioritizing of messages in the discussion. Maybe such a statement (that may for some readers be controversial) should be placed less prominently. This is said by a trialist who had a very public controversy with my national medicines agency (for closing down a HC trial during the first wave) so I do feel much the same as in this statement, nevertheless, maybe only a minority of the readers think this is key ? Just a comment, I guess the authors have had some relevant discussions on this…

**RESPONSE:** We have rearranged the discussion to focus on the study findings initially, and have now placed this sentence in a less prominent position.

Further: 
"This trial provides no support for these claims, and it supports the generally good short-term safety profile of these drugs.30 It challenges the World Health Organization "living" guidelines on COVID-19 chemoprevention which emphasised toxicity in COPCOV Lancet V2 Page 12 of 26 recommending against hydroxychloroquine. The same guideline also stated that prophylactic use "probably has a small or no effect on laboratory-confirmed COVID-19" with "moderate certainty" , and has no effect on mortality with "high certainty".26 Yet no deaths were reported in any of the pre-exposure prophylaxis studies, while the drugs have been well-tolerated with very low rates of treatment discontinuation (Figures S5, S6).31"
This is extremely important, which I strongly agree on, and actually it raised serious concern on the WHO handling of the pandemic on a very key point. I do not think the aftermath in WHO has handled this erroneous and very harmful reaction at a crucial point in the pandemic in a relevant way.

**RESPONSE:** Thank you very much for a very positive comment.

Later: "Now, in early 2023, with increasing coverage of effective vaccines," - possibly change to "Now, with increasing…" to strengthen generalizability of the statement
**RESPONSE:** We have changed accordingly. Line 356.

Another point. I think the discussion lacks a discussion of the interesting and very relevant finding of a reduction in the incidence of respiratory infections. Focus on this would make the results relevant in a future context, it indicates a general effect in respiratory infections.

**RESPONSE:** We agree that this was an important aspect but we documented too few other respiratory infections to make firm conclusions. This should be studied further.

Limitations are very well discussed.

**RESPONSE:** Thank you very much.

Finally, I want the congratulate the authors with a very well-planned, well conducted RCT, despite of the unreasonable resistance and irrelevant political decisions that made life very hard for people who really wanted to inform the world with precise data, on important issues during the COVID-19 pandemic. 
**RESPONSE:** Thank you very much for a very positive comment. Much appreciated!

*** Reviewer #3: 

The authors present the results of the long-awaited COPCOV study, which evaluated chloroquines as chemoprophylaxis for COVID-19. This may sound outdated now that we fight the pandemic with vaccines, but there were times when vaccines did not exist and the repurpose of existing drug was the best option to tackle the pandemic. Its results will not have a direct impact on the management of the current pandemic, but will help addressing challenges that caught the scientific community off-guard when the pandemic hit in 2020. The design and implementation of the study are sound
**RESPONSE:** Thank you very much for your very positive comments.

Major comments
- Figure 2 should contain the primary outcome.

**RESPONSE:** Figure 2 shows the Kaplan-Meier survival curves for the time to symptomatic COVID-19 as defined by a positive PCR test. This is the only component of the composite outcome which has an associated time-to-event. The two other components are defined by seroconversion between start and end dates so there is no time-to-event (only an interval). Given that a PCR positivity was clearly prespecified as the top of the hierarchy in the composite endpoint definition, we do feel it is appropriate to show these data individually.

- Please refer to the primary outcome as "symptomatic, laboratory-confirmed COVID-19" throughout the manuscript. That terminology is used in methods when the primary outcome is described, but not in the results section, which is confusing.

**RESPONSE:** We have made the suggested change throughout when discussing the primary outcome.

- I find the "primary outcome" section in results confusing:
o It starts reporting SARS-CoV-2 infection, which is not a primary outcome (it is actually an outcome that is not described in methods). Given that PCR tests were not done systematically, how is SARS-CoV-2 infection different from "symptomatic covid-19"? This needs to be clarified in methods.

**RESPONSE:** Sorry for the confusion. We have rewritten this section for clarity. Lines 279-292.

o Then it reports symptomatic [laboratory-confirmed] covid-19 which is the primary outcome

**RESPONSE:** This has also been rewritten for clarity. Lines 279-292.

o And then it reports comparisons that of non-primary outcomes: pcr-confirmed symptomatic covid-19, symptomatic covid-19 diagnosed by seroconversion, DBS symptomatic COVID-19.

o So, if you read this section fast (as most of us do), it is hard to identify the main result of the study. I think some rearrangement can make this section clearer.

**RESPONSE:** These have also been rewritten for clarity. Lines 279-292.

* Describe time to vaccination (KM curve) by arm. How were these patients handled?

**RESPONSE:** Thank you. We did not specify this objective in the protocol or in the analysis plan. We prefer to avoid this post-hoc analysis, as there is no reason to expect that provision of vaccines depended on treatment allocation. The timing of vaccination was multifactorial, depending on availability of vaccines in the countries, and the populations prioritised as well as individuals beliefs and personal choice. We encouraged participants to report when vaccinated. However, in the analysis of time to all-cause respiratory illness, we censored at the time of their vaccination. For the primary analysis, these participants were treated in the same way as those who were lost to follow-up i.e. in the ITT analysis those vaccinated were considered as not having had COVID-19 during the study period if they did not have COVID-19 before vaccination. In the methods we state the following which may be beneficial to readers who wondered about inclusion of vaccinated participants and what happened if they were subsequently vaccinated:

*The study recruited unvaccinated healthy, non-pregnant, adults aged between 17 and 70 years who were at risk of COVID-19...*

*Vaccines were deployed increasingly during the trial, and particpants were censored from the trial on receiving the first dose of vaccine. At that time they were reviewed and an “end of study” serum sample was taken before, or within three days, of their vaccination.*

* Make sure that the KM estimations are truly an ITT analysis, i.e., that patients who discontinue the drug are not censored (which is what the SAP says in page 14, but that is not an ITT analysis).

**RESPONSE:** Apologies if there has been any confusion on this point. Patients who discontinue the drug are not censored in the primary ITT analysis.

* It is odd that the main analysis is not a time-to-event analysis (e.g. by using a Kaplan Meier estimator). This means that the authors assume there is not right-censoring, when in fact there is according to Figure S7. I don't think that the results will change substantially, but I think a time-to-event analysis should be provided too (maybe as supplemental).

**RESPONSE:** The primary endpoint in the study is not a time-to-event variable. Only the PCR component has an associated time-to-event. For the seroconversion we do not know when this occurred (we only know the time interval). The statistical reviewer also noted this point and advised that we should make this clearer in the Results by stating: “The primary endpoint was symptomatic COVID-19 during the follow-up period” so that readers are clear that the primary outcome was not time-to-event. We have followed this advice.

* I think a KM curve for the main outcome with the following three arms should be added (maybe as supplemental): control, chloroquine, hydroxychloroquine.

**RESPONSE:** As discussed above, the KM approach is not applicable to this study.

* Effect estimates in an absolute scale (i.e., risk differences) should be provided in this study

**RESPONSE:** The absolute scale estimates are provided in the supplementary table S5.

Regarding the meta-analysis:
* Addressing the risk of bias of the component studies is standard practice in meta-analyses. See, for example Supp Table 1 of Garcia-Albeniz et al. I think it would be good to use the Cochrane risk of bias 2.0 tool to address the risk of bias of those studies not already evaluated for bias in the study by Garcia-Albeniz et al.

**RESPONSE:** Thank you**.** We have done this as suggested. S1_Supplemental Appendix; Figure S5.

The referees have correctly identified some discrepancies between our calculated risk ratios and those reported. Our meta-analysis uses risk ratios calculated for each study based on the reported data. We have added details to S1_Supplemental Appendix; Methods S2.

* Figure 3:
o The confidence interval by Seet et al does not match the original publication (0.44-0.97)

**RESPONSE:** The reviewer is correct to spot this inconsistency. The explanation is as follows. The CI provided by Seet et al is asymmetric on the log scale (the log differences are 0.46 and 0.33). The meta-analysis function we used to generate Figure 3 estimates the standard error (and hence study weight) from the width of the CI on the log scale and then re-calculates the CI relative to the point estimate provided. Because this interval is not centered around the point estimate, this results in the interval 0.47 to 1.04. We took a conservative approach using the estimate from the meta package which gives slightly wider CI than reported by Seet et al, but this makes very little difference to the overall meta-analysis estimate.

o The estimate by Polo et al should be 0.49 (0.00-2.29)

**RESPONSE:** This trial had 4 arms, 2 of which included hydroxychloroquine. This is identical to a factorially randomised trial, so we combined the two hydroxychloroquine arms and compared against the two arms not containing hydroxychloroquine. The consequent estimate is more precise.

o I would not include the article by McKinnon et al in this meta-analysis because of the same reason that Garcia-Albeniz (reference 23) used: although the authors do not label the study as post-exposure prophylaxis, they report that "60% [of study participants] reported contact with a COVID-19 positive patient before study entry

**RESPONSE:** We agree with the reviewer and the reasons given by Garcia-Albeniz for excluding this paper. Although we used the Garcia-Albeniz method, we had separately pre-defined the criteria for inclusion of papers into the meta-analysis (see S4_Statistical Analysis Plan and S1_Supplementary Appendix; Methods S2). We discussed at the time of analysis and felt that the pre-defined criteria warranted inclusion of McKinnon et al., despite the issues described. The weighting of this study 0.3% does not affect the overall treatment estimates.

o The estimate by Vijayaraghavan should be 0.85, 95% CI 0.35 to 2.07

**RESPONSE:** Thank you for spotting the small mistake. From the raw numbers provided in the paper there were 11/211 events in the HCQ arm and 12/203 events in the control arm. This gives a risk ratio of 0.88 (95% CI 0.40 to 1.95).

o The upper limit by Llanos-Cuentas should be 7.11

**RESPONSE:** We thank the reviewer for spotting this inconsistency. Checking the Llanos-Cuentas paper, the reported risk ratio is 1.69 (95% CI 0.41-7.11). This CI is also not symmetric on the log scale (which is why it had been changed in Figure 3), and it does not correspond to the reported number of events (5/36 in the HCQ arm and 3/32 in the control arm). We are uncertain how this risk ratio was calculated in the Llanos-Cuentas paper so we have now used the raw numbers provided in the paper to calculate a risk ratio and 95%CI: 1.48 with 95%CI 0.38 to 5.71.

o For consistency with Garcia-Albeniz et al, I think the authors should estimate the confidence interval of the meta-analyzed risk ratio using both a fixed (common) effect model and a random effects model.

**RESPONSE:** In our pre-defined analysis plan for the meta-analysis we specified using a common effects model only. Random effects models are not recommended when there are many small studies and few large studies (they overestimate the uncertainty).

o Figure 3 should have a note explaining the meaning of the grey squares around the point estimates

**RESPONSE:** Thank you we have added this. Lines 526-527.

o I think it would be good to complement the meta-analysis with the risk difference estimates, similar to Supp Figure 1 of Garcia-Albeniz et al.
* Figure S5

**RESPONSE:** We agree that the risk difference provides a more useful statistic for decision making. However, there is considerable variation in event rates across the included studies (which were done at different times in the COVID-19 pandemic) and the risk difference is therefore not expected to generalise well when event rates vary considerably. The risk ratio on the other hand should generalise well and so we have focused the meta-analysis on the relative scale.

Minor comments:
- If the sample size allows for an informative analysis, it would be nice to see a subgroup analysis of only healthcare workers

**RESPONSE:** Unfortunately the sample size does not allow for an informative analysis, as the majority of recruited participants were not healthcare workers (delays in recruitment meant that this group had often contracted COVID-19, or were subsequently the first to be offered vaccines).

- Results from the drug measurement described in the sixth line of "Procedures" section are not reported

**RESPONSE:** We have not measured drug levels as yet. We have clarified this in the text. Lines 206-207.

- I don't see the need to report p-values or concluding that "there were/were not statistically significant differences" because I consider this an outdated practice. [https://www.nature.com/articles/d41586-019-00857-9](https://protect-eu.mimecast.com/s/0TNvCpRwGSWE0ZI5FMby?domain=nature.com)

**RESPONSE:** Thank you. We are very sympathetic to this sentiment, although we stated in the pre-defined analysis plan that we would do statistical significance testing.

- There is a section "Interpretation of results" in the supplementary materials, that is inside the meta-analysis section and it reads like it does not belong there. 
**RESPONSE:** Thank you again for spotting this mistake. This should have been a separate section. We have now made this change.

*** Reviewer #4: 

This randomized controlled trial investigated the effect of using hydroxychloroquine (HCQ) as a preventive tool against symptomatic covid-19 (main endpoint). This study found that there is a borderline difference in the incidence of covid-19 RR=0.85 (0.72-1.00), p=0.05 and there was no effect against asymptomatic COVID-19 (RR: 0.96 (95% CI, 0.82 to 1.12), p=0.6) nor for all SARS-COV-2 infections in ITT analyses. In per protocol analysis, the primary outcome showed no effect.
The strengths of this study include the larger number of participants included (4600) compared to other clinical trials on hydroxychloroquine as a preventive medication. The trial took place in multiple countries.
My main concern is that the preventive effect is only observed among confirmed SAS-CoV-2 PCR (with a small number of cases confirmed by PCR) but there is no effect when the diagnosis is based on seroconversion or based on all SARS-CoV-2 cases or only asymptomatic cases. These drugs may have a small or no effect on COVID-19. 29% (13-14% loss to follow-up) of the initial randomized population have been excluded from per protocol analyses. Additionally, timing of this study may no longer be as important now that vaccines are available and the variants of SARS-CoV-2 are different.

**RESPONSE:** We thank the reviewer for their comments. We feel it is important to emphasise the prespecified hierarchy; that the PCR result is highly robust, the serological endpoints are less robust and, in particular, the dry blood spot analysis is least robust, and was not previously validated.

The description and the methods used in the meta-analysis raise some questions.
Main analysis:
1) "The study took place in multiple countries, including the United Kingdom, Asia, and Africa." Is there a geographical effect? Standard of cares, exposure to the SARS-CoV-2 (according to preventive policies), population are variable. It could be interesting to have analyses per continent or broad geographic areas.

**RESPONSE:** The proposed subgroup analyses would unfortunately be highly under-powered. In addition, this was not a pre-specified subgroup analysis. We do not have sufficient numbers for the primary outcome to perform the suggested subgroup analyses.

2) "The recruitment for the study took place between April 2020 and March 2022. Various variants, such as Alpha and Delta of the SARS-CoV-2, emerged during this period. Are these differences being considered? Regarding the vaccines, their effectiveness varied depending on the variants."

**RESPONSE:** There is no *a priori* reason that the different viral variants would have different susceptibility to antivirals, in particular those such as chloroquine and hydroxychloroquine, where the putative mechanism of action is host-directed. As such we feel that a subgroup analysis based on variants would be inadvisable and potentially misleading.

3) "The analyses combine two different molecules (CQ and HCQ). Is there a difference in their effects?"

**RESPONSE:** Thank you. Again *a priori* there is no reason to expect a difference in effect between these two drugs. These two compounds have very similar pharmacological activities, and we pre-specified their combined analysis in the SAP, based on earlier advice by the study’s independent DSMB.

4) "How was adherence to the intervention assessed? Plasma concentrations of HCQ or CQ were not measured."

**RESPONSE:** Adherence was assessed by a count and reconciliation of medication conducted at the in-person reviews at D30, D60 and D90. Please see S3_Protocol.

5) "The power calculation was originally presented for a total of 40,000 participants. However, only 4,652 people were ultimately randomized. What is the expected detectable effect size?

**RESPONSE:** Thank you. Although we are cautious conducting anything which could be interpreted as a post-hoc power calculation, given the smaller numbers recruited, and the much higher event rate than initially expected we have added the following text, which is careful to use only the event rate:

*The primary outcome was changed to include seroconversion. This resulted in a much greater event rate than initially forecasted. A total sample size of 4600 would have >80% power to detect the same treatment effect (23% reduction) assuming a 12% event rate in the control arm.*

The event rate in the control arm was approximately 12%.

6) The authors have longitudinal data with almost 2 years of follow-up. Why is the primary analysis based on a Fisher exact test? I wonder if the results would be different with a survival model and Kaplan-Meier analysis (Kaplan-Meier was performed for respiratory infections but not for COVID-19). The main result for the primary outcome is borderline significant (RR=0.85 [0.72-1.00], p=0.05), it is difficult to interpret this result. Results for all SARS-CoV-2 infection suggest no effect of CQ and CHQ.

**RESPONSE:** As explained above in the response to reviewer 3, the primary endpoint in the study is not a time-to-event variable. Only the PCR component has an associated time-to-event. For the seroconversion we do not know when this event occurred (we only know the time interval). The statistical reviewer also noted this point and advised that we should make this clearer in the Results by stating: “The primary endpoint was symptomatic COVID-19 during the follow-up period” so that readers are clear that the primary outcome was not time-to-event. We have followed this advice.

7) Table 2 mentions missing data for the primary outcome. What was the number of missing data? This should be specified.

**RESPONSE:** Thank you for this comment. We have added this detail in the text.

*Primary outcome data were missing for 1114 participants (24%) for the serology component, mainly because of loss to follow-up.*

8) In the discussion, statement is not correct "This protective effect is consistent with previous studies10,11,14-22" I looked at the first references mentioned: ref 10, Syed et al. 2021 writes "It is concluded that the PrEP HCQ does not significantly prevent COVID-19 among high-risk HCPs." Abella et al. 2021 wrote "there was no clinical benefit of hydroxychloroquine administered daily for 8 weeks as pre-exposure prophylaxis in hospital-based HCWs exposed to patients with COVID-19." Same for Rajasingham et al. 2021: Pre-exposure prophylaxis with hydroxychloroquine once or twice weekly did not significantly reduce laboratory-confirmed COVID-19 or COVID-19-compatible illness among healthcare workers.

**RESPONSE:** Thank you. We agree these studies did not meet statistical significance, but the quoted statements from the papers assume that failure to meet statistical significance means that there was no effect, when the studies were only powered to detect large effects (i.e. they could not have detected the moderate benefits shown in the COPCOV trial). As a result, all these studies had wide confidence intervals and p-values >0.05, but of course that does not mean that the null is true. This is the value of meta-analysis. By combining data moderate effects become detectable. The results of the COPCOV trial are consistent with the results of the Garca-Albeniz et al. meta-analysis of the previous studies, which was based on the reported primary endpoints. In summary the individual studies did not provide conclusive results because the differences are not large, whereas the combined data does provide clear evidence of benefit.

9) In the Abstract, « Published studies suggest clinical benefit" is not right. Several randomized trials have shown no effect of HCQ on covid19 as treatment or preventive tool… These results could be compared with negative results from previous meta-analysis studying HCQ as preventive tool (Hong et al. 2023). Previous meta-analyses of RCTs on HCQ as treatment also reported no effect on COVID-19.
Hong H, Friedland A, Hu M, Anstrom KJ, Halabi S, McKinnon JE, Amaravadi R, Rojas-Serrano J, Abella BS, Portillo-Vázquez AM, Woods CW, Hernandez AF, Boulware DR, Naggie S, Rajasingham R. Safety and efficacy of hydroxychloroquine as prophylactic against COVID-19 in healthcare workers: a meta-analysis of randomised clinical trials. BMJ Open. 2023 Jun 16;13(6):e065305. doi: 10.1136/bmjopen-2022-065305. PMID: 37328184; PMCID: PMC10276967.

**RESPONSE:** Thank you but again we repeat that a p-value > 0.05 does not prove the null. This is unfortunately the standard interpretation in many medical journals, but it is incorrect. The previous meta-analysis by Garcia-Albeniz et al. showed that the hydroxychloroquine randomised trial data were compatible with a moderate preventive effect in pre-exposure prophylaxis. Our results substantially strengthen this assertion.

10) I wonder whether the protocol was pre-registered on an open-platform.

**RESPONSE:** An earlier version of the protocol was published in 2020 in Wellcome Open Research. <https://wellcomeopenresearch.org/articles/5-241>, and the final version of the protocol has been on the COPCOV webpage since the 5^th^ of May 2022 <https://www.tropmedres.ac/covid-19/copcov>.

Meta-analysis: The meta-analysis suggests a protective effect. It is important to underline that this study has a weight of 61%.
it is unclear what RR (or Odds Ratio or Hazard Ratios; adjusted or not) was used.

**RESPONSE:** Our meta-analysis uses risk ratios for each study. The primary estimate in the Rajasingham et al. paper was a hazard ratio so we calculated a risk ratio and corresponding 95% CI from the reported data (58/989 events in the HCQ arms versus 39/494 in the control arm).

We have clarified this further with the following sentence in the caption of the figure:

*Risk ratios were determined for all studies based on the reported data, apart from Seet et al., which was cluster randomised and a recalculated adjusted risk ratio was used (Methods S2).*

As well as this Figure 3 says “Risk ratio” on the x-axis.

A table describing the individual included studies could be useful to understand what was the intervention, the dose, the placebo, the duration, and the endpoint, study population (general population vs health workers).

Thank you we have added a table with the summary characteristics of the trials considered in the meta-analysis. S1_Supplemental Appendix; Table S7.

1) Heterogeneity could be added in the main text

**RESPONSE:** We have added a line pointing out these differences.

*Although the studies reported slightly different outcomes, used different doses and for different durations...*

2) In the supplementary material, it is not clear what meta-analysis model has been used: fixed or random effect model?

**RESPONSE:** We used fixed effects (also known as common effects) meta-analysis. This is written in Figure 3 and predefined in the SAP. Random effects meta-analysis can result in inappropriate confidence intervals when there are many small studies and few large studies. In this context where there is very little evidence of heterogeneity the two methods give identical results.

3) This meta-analysis pooled different interventions: I looked at the studies with the most important weight in the meta-analysis. The meta-analysis pooled different endpoints across the studies (PCR positive or probable covid-19, symptomatic covid only, infections…). I have not checked all the included studies but some of them included a different placebo (Vitamin C).

**RESPONSE:** We agree the studies are heterogenous in this regard. Importantly, determining seroconversion early on in the pandemic was not possible. For simplicity we have taken each study’s primary endpoint.

4) In Rajasingham 2020, the Hazard ratio is .74 (95% CI, 0.45-1.20) for COVID-19 compatible with symptoms and the forest plot in this manuscript reported slightly different values 0.74 (95% CI, 0.50-1.10). Placebo was Vitamin C.

**RESPONSE:** The primary estimate in the Rajasingham et al. paper was a hazard ratio so we calculated a risk ratio and corresponding 95% CI from the reported data (58/989 events in the HCQ arms versus 39/494 in the control arm).

We have clarified this further with the following sentence in the caption of the figure:

*Risk ratios were determined for all studies based on the reported data, apart from Seet et al., which was cluster randomised and a recalculated adjusted risk ratio was used (Methods S2).*

-19? In the original study of Seet, Odds Ratio are provided and not Relative Risk. And the aOR is different: 0.70 [0.44 ; 0.97] for SARS-CoV2-infection.

**RESPONSE:** Table 3 in Seet et al. does provide an adjusted risk ratio estimate of 0.70 (0.44, 0.97), note this is a 98.75 CI. However, this CI is asymmetric on the log scale (log differences are 0.46 and 0.33). The meta-analysis function used to generate Figure 3 estimates the standard error (and hence to study weight) from the width of the CI on the log scale and then re-calculates the CI relative to the point estimate provided. Because this interval is not centered around the point estimate, this results in the interval 0.47 to 1.04. We use the conservative estimate from the meta package.

6) I also wonder whether there is a dose-effect of HCQ/CQ (using meta-regression)

**RESPONSE:** This is an interesting question. An individual patient data meta-analysis might be more appropriate to answer this question. As this was not pre-specified in the SAP we have left out of this analysis.

I suggest to add line numbering in the .pdf

**RESPONSE:** We have done this thanks.

To conclude, I wonder to what extent a prevention policy for SARS-CoV-2 using hydroxychloroquine can be implemented and useful now that vaccines are available (as it is mentioned in the discussion). The recommendation would be to advise the general population to take hydroxychloroquine regularly every day. Is this sustainable and feasible? Unlike vaccines, which have shown a low preventive effect, where we only need one injection and boosters.

**RESPONSE:** We agree. We have added to the summary:

*Although hydroxychloroquine or chloroquine are unlikely to be used in COVID-19 prevention at this stage of the pandemic, they could have been deployed with benefit earlier, and they could have value in future pandemics. As in other areas of medicine, randomised controlled trial evidence is essential in evaluating therapeutics in the context of a pandemic. Trials should be facilitated and protected so that evidence is generated rapidly and evidence -based policies can be implemented without delay to allow timely interventions.*

***

Any attachments provided with reviews can be seen via the following link:

__________________________________________________
In compliance with data protection regulations, you may request that we remove your personal registration details at any time. (Use the following URL: [https://www.editorialmanager.com/pmedicine/login.asp?a=r](https://protect-eu.mimecast.com/s/V3JmCqVRJC36yvsj5jVm?domain=editorialmanager.com)). Please contact the publication office if you have any questions.
