# Supplementary material for: Evaluation of hydroxychloroquine or chloroquine for the prevention of COVID-19 (COPCOV): A double-blind, randomised, placebo-controlled trial
Source: PLoS Med. 2024 Sep 12;21(9):e1004428. doi: 10.1371/journal.pmed.1004428 (PMC11392261; doi:10.1371/journal.pmed.1004428)
Supplement: Attachment — Submitted filename: COPCOV PLOS MED response to editors and reviewers 06_12_23.docx [file pmed.1004428.s009.docx]

Please find the responses below. In addition to the responses to the Editors and Reviewers we have standardised the reporting of significance tests in the tables to 3 significant figures, and uploaded a newer version of the supplementary materials.

Requests from the editors:

Please remove the information on funding and the publishing licence from the abstract page. In the event of publication, information on funding will appear in the article metadata, via entries in the submission form.

Thank you we have now removed this information. 

Around line 87 (abstract), please add a sentence to mention the intended trial recruitment and explain briefly why it could not be achieved.

Thank you we have added the intended trial recruitment and the reasons why this was not achieved.

“Recruitment of 40,000 (20,000 HCQ arm, 20,000 CQ arm) participants was planned but was not possible because of protracted delays. These resulted from controversies surrounding HCQ, regulatory reactions to false claims of frequent cardiotoxicity, clinical trial bureaucracy, rapid vaccine rollout in some countries, and the limited shelf-life of the trial medicines.”

At lines 94, 95, 96 & 99 (abstract), we suggest substituting colons for the four semicolons.

Thank you: we have added these. 

Please remove the sentence at line 107 ("Difficulties conducting ...", abstract), as this issue can be fully dealt with in the Discussion (main text).

This sentence has been removed.

Please reformat the 'Author summary' so that the three subsections each comprise 3-4 short points of 1-2 sentences each. Please use the active voice (e.g., "We found ...") in one or two points.

Thank you we have made these changes.

Regarding the summary of outcomes at line 218, please include a full list of all outcomes. We note that there are 'other outcomes' specified, including impact on healthcare costs. We ask you to report all outcomes for which data are available in the present paper.

Thank you. We have reported all outcomes for which there are data available. We have not conducted the genetic and biochemical analyses nor the health economic analyses. We have added the following sentence to clarify this:

“Other pre-specified tertiary outcomes: genetic and baseline biochemical markers associated with symptomatic COVID-19, respiratory illness and disease severity; and health economic analyses of HCQ and CQ prophylaxis on costs and quality of life measures have not been conducted, and are not reported here.”

At line 325, noting 'preventitive', we suggest using 'preventive' throughout.

This change has been made throughout.

Please adapt the reference call-outs to journal style throughout, e.g., "... equivalent [25,26]." (noting the absence of spaces within the square brackets).

Thank you we have made these changes.

Despite the retraction, please complete the citation for reference 4 with full access details. Please revisit reference 24, which may be missing some information.

This is now completed.

Comments from the reviewers:

We would like to thank all reviewers again for their high-quality and thorough reviews, as well as supportive comments.

*** Reviewer #1: 

Thank you to the authors for addressing my previous comments well, I have no further issues to raise.

*** Reviewer #2: 

I have no further comments, the authors have responded very adequately. It´s a fine study

*** Reviewer #3: 

All my comments have been addressed
