# Supplementary material for: Evaluation of hydroxychloroquine or chloroquine for the prevention of COVID-19 (COPCOV): A double-blind, randomised, placebo-controlled trial
Source: PLoS Med. 2024 Sep 12;21(9):e1004428. doi: 10.1371/journal.pmed.1004428 (PMC11392261; doi:10.1371/journal.pmed.1004428)
Supplement: Attachment — Submitted filename: Response to editors comments 08_01_24.docx [file pmed.1004428.s010.docx]

Thank you for your comments. Please find our responses below.

Requests from the editors:

In the 'Financial disclosure' section (submission form), the information about the CC-BY licence is superfluous and can be removed, as this licence is standard at PLOS Medicine.

Thank you we have removed this from the submission form. 

Please remove the quoted secondary outcome findings from the abstract, retaining the primary outcome findings, results of the prespecified meta-analysis and information about safety.

Thank you. We respectfully request retaining the prespecified endpoints in the abstract as they provide a valuable context to the primary outcome. This is particularly important for the workdays lost as, in a double-blind trial with a well-tolerated medication, it substantially reduces the uncertainty around the primary result.

At line 152 (Introduction), please adapt the wording to "a false claim".

We have made this change.

At line 163 (Introduction) please adapt the text to state that the trials did not individually provide evidence of benefit.

Thank you we have adapted this sentence as below. As Seet et al. reported benefit, we changed the wording slightly.

*Taken together these studies point towards moderate preventive efficacy even though individually most were underpowered to demonstrate benefit [23].*

Noting the information around line 288 (Results) regarding confirmed COVID-19 infection, we ask you to add a supplementary table listing causes of respiratory illness.

This has been added- table S8 in the supplementary appendix.

At line 330 (Discussion) we feel that "... evidence of moderate preventive efficacy" is an overstatement and ask you to amend the wording to "... some evidence of preventive efficacy" or similar.

We have removed “moderate” and substituted “evidence indicating”.

At line 331, we ask you to adapt the text to "... an apparent 15% reduction", noting the equivocal p value.

Thank you. The value was 15% but we accept that this is a small amount with some uncertainty and we must be careful not to overstate. We therefore added “modest” to the next sentence.

*This modest protective efficacy is consistent with the aggregated results of previous smaller studies [10,11,14-22].*

At line 332 we suggest "efficacy" rather than "effect", as elsewhere in the text.

We agree and have made this change.

Again at line 332, we ask you to revisit "protective effect is consistent with previous smaller studies". We believe that the previous studies did not provide evidence of significant benefit, and we ask you to adapt the wording to indicate this clearly.

We agree. We have rephrased this to indicate that it is the combined rather than individual effect that was significant.

*This modest protective efficacy is consistent with the aggregated results of previous smaller studies [10,11,14-22].*

At line 360, we suggest removing "outright fraud", although we are aware that this is not an unfounded statement.

We would like to change to “fabricated data” as there was unequivocal evidence of this in the Surgisphere debacle.

At line 374, we ask you to remove the sentence "This must not be allowed ...".

We have done this, thank you.

At line 389, we ask you to remove the word "strong", which seems an overstatement based on the numbers quoted.

We have replaced this with “indicates consistent evidence” (as the studies were all in the same direction).

At line 397, please remove the phrase "and not impeded".

We have done this.

At line 403, please remove the information about study funding, which is present in the financial disclosures which will appear in the article metadata in the event of publication.

We have done this.

Please edit reference 4: we believe that this can be truncated prior to "Epub ahead of print" so as to retain the essential information.

Thank you we have truncated prior to “Epub ahead of print” but do please feel free to edit references as you see fit.
