# Supplementary material for: Evaluation of hydroxychloroquine or chloroquine for the prevention of COVID-19 (COPCOV): A double-blind, randomised, placebo-controlled trial
Source: PLoS Med. 2024 Sep 12;21(9):e1004428. doi: 10.1371/journal.pmed.1004428 (PMC11392261; doi:10.1371/journal.pmed.1004428)
Supplement: Attachment — Submitted filename: Response to requests from Editors.docx [file pmed.1004428.s012.docx]

Many thanks. We agree with all the Editorial suggestions to abbreviate chloroquine (to CQ) and hydroxychloroquine (HCQ) in the text, and will agree with any further recommendations regarding abbreviations. We have made the changes suggested below. There are some instances elsewhere where the unabbreviated form, in our opinion reads better, and have left these, although if you prefer these abbreviated later, we will accept these suggestions at the proofing stage. Also when referring to the drug substance, we have left them unabbreviated.

Requests from Editors:

Should that be "HCQ" at line 85 (abstract)?

Thanks- have made the change.

At line 107, please make that "... provided a moderate protective benefit ...".

Change made.

At line 121 (author summary), please indicate the abbreviations for CQ and HCQ and use these abbreviations thereafter in the author summary.

We have done this.

At line 164, alongside "to indicate a lack of efficacy", do you mean for CQ/HCQ or for the various agents mentioned earlier in the sentence more generally? You may wish to add a few words to clarify.

Clarified by adding the words “for HCQ”

Again in the introduction (main text) HCQ can be used from line 161 onwards.

Thanks made these changes.

Please include a completed CONSERVE checklist as a supplementary file, if relevant.

The changes to the study are well-documented in the text, but we have completed this checklist to help readers identify these.
